# CDK-mediated activation of the SCF^FBXO28^ ubiquitin ligase promotes MYC-driven transcription and tumourigenesis and predicts poor survival in breast cancer

**DOI:** 10.1002/emmm.201202341

**Published:** 2013-06-14

**Authors:** Diana Cepeda, Hwee-Fang Ng, Hamid Reza Sharifi, Salah Mahmoudi, Vanessa Soto Cerrato, Erik Fredlund, Kristina Magnusson, Helén Nilsson, Alena Malyukova, Juha Rantala, Daniel Klevebring, Francesc Viñals, Nimesh Bhaskaran, Siti Mariam Zakaria, Aldwin Suryo Rahmanto, Stefan Grotegut, Michael Lund Nielsen, Cristina Al-Khalili Szigyarto, Dahui Sun, Mikael Lerner, Sanjay Navani, Martin Widschwendter, Mathias Uhlén, Karin Jirström, Fredrik Pontén, James Wohlschlegel, Dan Grandér, Charles Spruck, Lars-Gunnar Larsson, Olle Sangfelt

**Affiliations:** 1 Department of Cell and Molecular Biology, Karolinska InstitutetStockholm, Sweden; 2 Department of Oncology/Pathology, Cancercentrum Karolinska, Karolinska InstitutetStockholm, Sweden; 3 Department of Microbiology, Tumor and Cell Biology, Karolinska InstitutetStockholm, Sweden; 4 Cancer Cell Biology Research Group, Department of Pathology and Experimental Therapeutics, Faculty of Medicine, University of BarcelonaBarcelona, Spain; 5 Department of Immunology, Genetics and Pathology, Rudbeck Laboratory, Uppsala UniversityUppsala, Sweden; 6 Oregon Health and Science University, Knight Cancer InstitutePortland, OR, USA; 7 Department of Medical Epidemiology and Biostatistics, Karolinska InstitutetStockholm, Sweden; 8 Translational Research Laboratory, Catalan Institute of Oncology, IDIBELL, L'Hospitalet de Llobregat; and Physiological Sciences II Department, Bellvitge Campus, University of BarcelonaSpain; 9 Sanford-Burnham Medical Research InstituteLa Jolla, CA, USA; 10 NNF Center for Protein Research, Faculty of Health SciencesCopenhagen, Denmark; 11 Department of Biotechnology, KTH/Alba Nova University CenterStockholm, Sweden; 12 Department of Women's Cancer, UCL Elizabeth Garrett Anderson Institute for Women's Health, University College LondonUnited Kingdom; 13 Department of Laboratory Medicine, Center for Molecular Pathology, Lund University, Skåne University HospitalLund, Sweden; 14 Department of Biological Chemistry, David Geffen School of Medicine, University of CaliforniaLos Angeles, CA, USA

**Keywords:** Breast cancer, CDK, F-box protein, FBXO28, MYC

## Abstract

SCF (Skp1/Cul1/F-box) ubiquitin ligases act as master regulators of cellular homeostasis by targeting key proteins for ubiquitylation. Here, we identified a hitherto uncharacterized F-box protein, FBXO28 that controls MYC-dependent transcription by non-proteolytic ubiquitylation. SCF^FBXO28^ activity and stability are regulated during the cell cycle by CDK1/2-mediated phosphorylation of FBXO28, which is required for its efficient ubiquitylation of MYC and downsteam enhancement of the MYC pathway. Depletion of FBXO28 or overexpression of an F-box mutant unable to support MYC ubiquitylation results in an impairment of MYC-driven transcription, transformation and tumourigenesis. Finally, in human breast cancer, high FBXO28 expression and phosphorylation are strong and independent predictors of poor outcome. In conclusion, our data suggest that SCF^FBXO28^ plays an important role in transmitting CDK activity to MYC function during the cell cycle, emphasizing the CDK-FBXO28-MYC axis as a potential molecular drug target in MYC-driven cancers, including breast cancer.

## INTRODUCTION

The ubiquitin–proteasome-system (UPS) plays critical roles in a vast number of cellular processes (Bashir & Pagano, 2003; Hershko & Ciechanover, 1998; Pickart, 2004) and deregulation of the UPS is implicated in the pathogenesis of various diseases including cancer. The substrate specificity of the ubiquitin system is achieved through the use of a large number of E3 ubiquitin ligases that selectively bind to and mediate the covalent attachment of a single or chains of ubiquitin to substrates (Weissman, 2001). Poly-ubiquitylated substrates are recognized and degraded by the proteasome. However, ubiquitin conjugation also has non-proteolytic roles in processes such as transcription (Chen & Sun, 2009). The SCF (Skp1/Cul1/F-box) multi-subunit E3s generally consist of the core subunits Skp1, Cul1 and the ring-finger protein Roc1 bound to a variable F-box protein that dictates substrate specificity (Bai et al, 1996; Cenciarelli et al, 1999; Skowyra et al, 1997). Up to 70 different genes encoding F-box proteins have been identified in the human genome, but only a handful have been characterized (Skaar et al, 2009), some with potent functions as either tumour suppressors or tumour promoters (Frescas & Pagano, 2008; Nakayama & Nakayama, 2006).

The MYC oncoprotein/transcription factor controls the expression of up to 15% of the cellular transcriptome, and serves as a master regulator of various biological functions (Adhikary & Eilers, 2005; Larsson & Henriksson, 2010; Meyer & Penn, 2008). Together with MAX, MYC binds to specific E-box DNA recognition sequences (CACGTG or similar sequences) at target genes and activates transcription by recruiting multiple co-activator complexes (Adhikary & Eilers, 2005; Larsson & Henriksson, 2010; Meyer & Penn, 2008). Recent findings suggest that MYC functions as an amplifier of expression from active or poised promoters by stimulation of transcription elongation (Bouchard et al, 2004; Eberhardy & Farnham, 2001; Guccione et al, 2006; Lin et al, 2012; Nie et al, 2012; Rahl et al, 2010). Overexpression of MYC induces oncogenic transformation, and its deregulation in multiple human malignancies contributes to a large fraction of human cancers (Adhikary & Eilers, 2005; Larsson & Henriksson, 2010; Meyer & Penn, 2008).

Here, we identify a hitherto uncharacterized F-box gene, FBXO28, encoding a cell cycle regulated protein with a critical function in tumour cell proliferation. FBXO28 assembles an SCF^FBXO28^ ubiquitin ligase whose activity is regulated during progression through the cell cycle by cyclin-dependent kinase 1/2 (CDK1/2)-mediated phosphorylation. Phosphorylation of FBXO28 at serine 344 enables the SCF^FBXO28^ ubiquitin ligase to target the oncoprotein MYC for non-proteolytic ubiquitylation, thereby fueling MYC-driven transcription, proliferation and tumourigenesis. Importantly, we find that FBXO28 expression and phosphorylation to be strongly associated with poor prognosis and worse overall survival (OS) in human breast cancer.

## RESULTS

### The F-box protein FBXO28 regulates tumour cell proliferation

**Figure 1:**
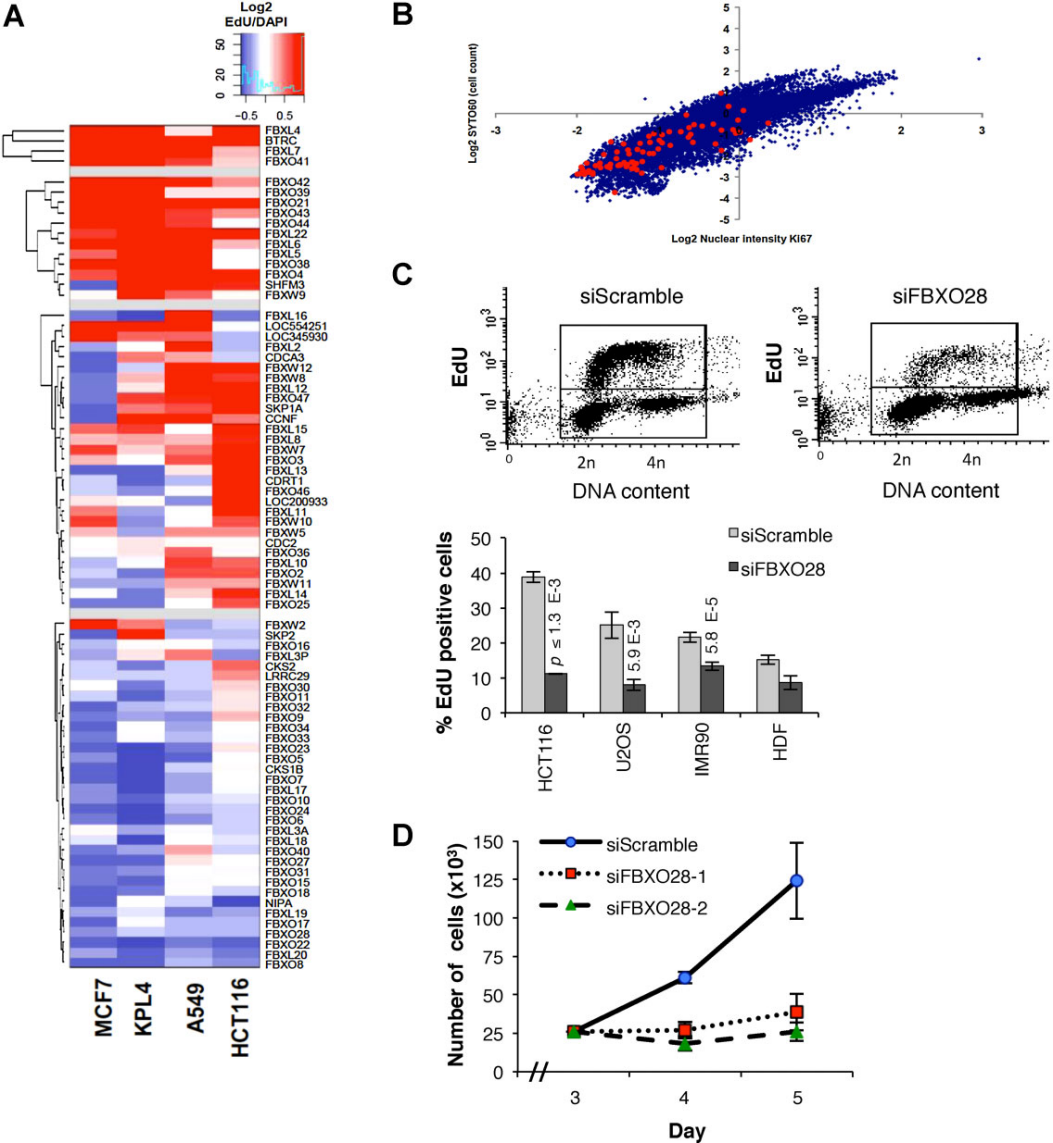
FBXO28 regulates tumour cell proliferation Hierarchical partitioning around medoids (PAM) clustering of the *z*-score results of the F-box RNAi screen in the indicated cell lines. The genes are ranked according to EdU incorporation, with more EdU (red) at the top and less EdU (blue) at the bottom. The *p*-values (Student's *t*-test) for siFBXO28 compared to control siRNA were *p* = 0.00729 in MCF7, *p* = 0.00366 in KPL4, *p* = 0.0207 in A549 and *p* = 0.0200 in HCT116 (*n* = 4 replica per group and cell line).Global siRNA screen in KPL4 breast cancer cells, using Ki-67 as a proliferation marker. Scatter plot distribution of *z*-score results, where red dots represent siRNAs targeting F-box genes.EdU incorporation monitored by flow cytometry following siRNA depletion of FBXO28 for 48 h. Upper panels: representative FACS analysis in U2OS osteosarcoma cells. Lower panel: Percentage of EdU positive cells upon FBXO28 silencing in the indicated cell lines. HCT116 and U2OS were pulsed with EdU for 20 min, while IMR90 and HDFs were pulsed for 4 h before analysis. Bars indicate standard error of the mean (SEM) from three independent experiments in HCT116, U2OS and IMR90 and two experiments in HDF. *p*-values (shown above the bars) were determined by the Student's *t* test compared to siScramble for each cell line. *p*-values not calculated for HDF.Growth curves of U2OS cells transfected with two different FBXO28 siRNAs compared with scrambled control siRNAs. The data represent the mean ± SEM of three cell counts for each time point and condition. Note that cells were exposed to siRNAs for 48 h before re-plating in equal numbers, followed by cell counting at days 3, 4 and 5. No significant difference in cell number was evident after 48 h siRNA transfection (unpublished data).

To identify novel F-box proteins required for tumour cell proliferation, we performed a high-throughput short interfering RNA (siRNA)-based screen using an F-box siRNA library that targets the complete set of human F-box genes. Cells from different types of tumour cell lines were reverse transfected with pools of F-box specific siRNAs and the effects on proliferation were assessed by EdU incorporation using the Cell Spot MicroArray method (CSMA) (Rantala et al, 2011). This screen identified several previously uncharacterized F-box genes, the depletion of which caused clear and consistent inhibitory effects on proliferation (Fig 1A).

To validate these results, we applied the CSMA method using a library of siRNAs (Qiagen Druggable genome v1.0) comprising a total of 6135 genes, including 53 F-box genes, in KPL4 breast cancer cells using Ki-67 as a proliferation marker (Fig 1B). Interestingly, of the top 200 siRNAs that most potently reduced Ki-67 expression, nine were F-box genes (Supporting Information Table S1). Enrichment analysis of functional categories using DAVID (Dennis et al, 2003) confirmed the striking enrichment of the F-box gene family (*p* = 3.0E−7). In particular, FBXO28 depletion led to a potent inhibitory effect on proliferation in both siRNA screens (Fig 1A and Supporting Information Table S1) and was therefore selected for further in depth analysis.

Flow cytometry and time-lapse microscopy analyses confirmed that depletion of FBXO28 resulted in a progressive loss of proliferation, evident from around 36–48 h of siRNA silencing in several different tumour cell lines (Fig 1C and D; Supporting Information Fig S1A–C). Knockdown of FBXO28 also decreased the proliferation of normal human IMR90 cells and human diploid fibroblasts (HDFs), although to a lesser extent than the tumour cell lines (Fig 1C). This was not due to induced cell death (unpublished data), suggesting a cytostatic mode of action. Together these results support an important function for FBXO28 in the regulation of cellular proliferation.

### FBXO28 is phosphorylated by CDK1/2 and is part of an SCF ubiquitin ligase complex

**Figure 2:**
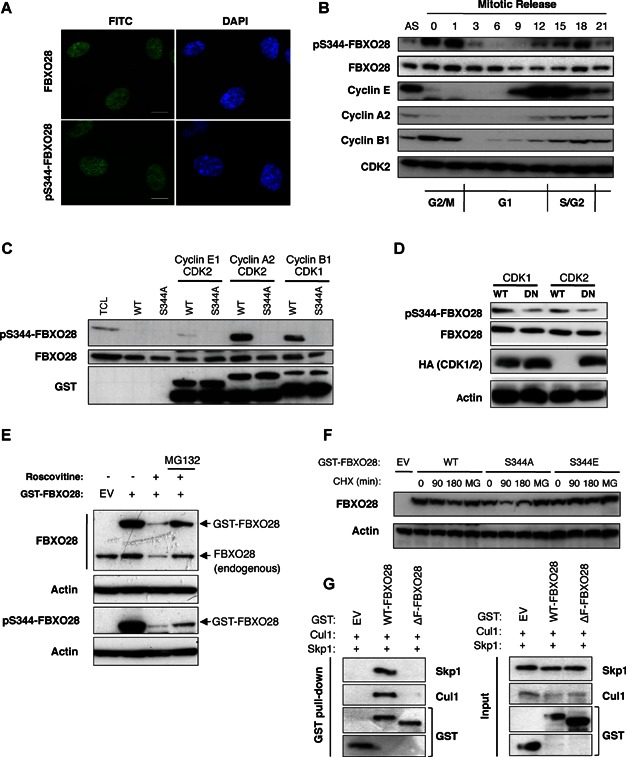
FBXO28 is a nuclear protein phosphorylated by CDK1/2 that assembles an SCF ubiquitin ligase complex Immunostaining for endogenous FBXO28 protein in IME cells with FBXO28 (upper panel) and pS344-FBXO28 (lower panel) antibodies. Nuclei were counterstained with Hoechst (blue). Scale bars, 10 µm.Cell cycle profile of FBXO28 expression. HeLa cells were released by mitotic shake-off, re-plated and harvested at the depicted time-points. Protein extracts from whole cell lysates were used for immunoblotting (IB) analysis with the indicated antibodies. The presence of cells in the G2/M-, G1- and S-phases of the cell cycle was documented by flow cytometry (unpublished data). AS, asynchronous cells.*In vitro* translated FBXO28 (WT or S344A) protein was subjected to *in vitro* kinase assays using recombinant GST-Cyclin/CDK complexes as depicted. IB analysis was performed with the indicated antibodies. Total cell lysate (TCL) from U2OS cells was included as a control.Protein extracts from HEK293 cells transfected with the indicated HA-tagged expression constructs, or untagged CDK2 construct, were analysed by IB analysis with the indicated antibodies. WT, wild-type; DN, dominant-negative.Lysates of Cos7 cells transfected with GST-FBXO28 or empty vector (EV) and treated for 4 h with the CDK inhibitor Roscovitine, with or without MG-132, were analysed by IB analysis. Upper panel: Exogenous (GST-FBXO28) and endogenous FBXO28 protein levels were detected using FBXO28 antibodies. Lower panel: Phosphorylated FBXO28 level was analysed by IB using pS344-FBXO28 antibodies on a separate filter. Actin was used as input control.Cycloheximide (CHX) chase analysis of WT and mutant forms of FBXO28 (S344A and S344E) in Cos7 cells. Chased cells were treated for 4 h with MG-132 (MG), as depicted.FBXO28 is part of an SCF complex. HEK293 cells were cotransfected with expression constructs encoding Cul1 and Skp1 together with GST-tagged WT-FBXO28, ΔF-FBXO28 or EV and FBXO28 was purified on GST-beads. Bound proteins were eluted and detected by IB using the indicated antibodies.

The human *FBXO28* gene encodes a highly conserved nuclear protein (Fig 2A; Supporting Information Fig S2B) of 368 amino acids (aa) that contains an F-box motif in its N-terminus (aa 67–94, Supporting Information Fig S2A), but lacks other discernable domains. Tryptic phosphopeptide analysis of immunopurified FBXO28 revealed a peptide with a high confidence score for phosphoserine modification at amino acid 344 (*LREVMESAVGNSSGSGQNEE****pS****PR*) that conforms to a conserved CDK consensus phosphorylation motif (S/T)P*X*(K/R) at the FBXO28 C-terminus (Supporting Information Fig S2C). Using antibodies specifically recognizing FBXO28 phosphorylated on S344 (Supporting Information Fig S2D–F) we found that pS344-FBXO28 is also mainly localized to the nucleus (Fig 2A; Supporting Information Fig S2D and F). Analysis of FBXO28 levels during progression through the cell cycle in HeLa cells showed that FBXO28 phosphorylation is minimal in G1 phase and high during S-G2/M phase (Fig 2B). The total FBXO28 level also declined in G1 with a slight delay and subsequently increased later in the cell cycle, coinciding with FBXO28 phosphorylation (Fig 2B). Notably, the FBXO28 phosphorylation pattern resembled the expression of the S and G2 cyclins, cyclin A2 and cyclin B1 (Fig 2B), raising the question whether FBXO28 could be phosphorylated by the associated CDKs. Indeed, *in vitro* kinase assays using purified recombinant cyclin/CDK complexes, revealed that both cyclin A-CDK2 and cyclin B-CDK1, but not cyclin E-CDK2, efficiently phosphorylated *in vitro*-translated wild-type (WT) FBXO28, but not a mutant protein where S344 was replaced with alanine (S344A) (Fig 2C). Consistently, overexpression of dominant-negative (DN) CDK1 or CDK2 mutants reduced phosphorylation at S344 (Fig 2D). Interestingly, treatment of cells with the CDK1/2 inhibitor roscovitine not only reduced FBXO28 phosphorylation, but also had a profound effect on the total abundance of FBXO28 protein (Fig 2E). Coincubation with the proteasome inhibitor MG-132 almost fully restored FBXO28 protein levels (Fig 2E), suggesting that CDK-mediated phosphorylation influenced the turnover of FBXO28. This was verified by cycloheximide (CHX) chase analysis, demonstrating more rapid degradation of the S344A-FBXO28 mutant as compared to WT-FBXO28 or a phosphomimetic S344E mutant (Fig 2F). Deletion of the F-box domain (which connects F-box proteins to the E3 ligase complex via Skp1) in FBXO28 (ΔF-FBXO28) did not impede phosphorylation at S344 but stabilized the protein (Supporting Information Fig S2G and H), indicating that FBXO28 might regulate its own turnover through auto-ubiquitylation. In order to test whether FBXO28 forms an SCF^FBXO28^ complex in cells, we performed coimmunoprecipitation experiments with WT-FBXO28 or ΔF-FBXO28 and epitope-tagged SCF subunits. As shown in Fig 2G, WT-FBXO28 efficiently copurified with the core ligase, whereas ΔF-FBXO28 failed to do so, demonstrating that FBXO28 is part of an SCF ubiquitin ligase capable of interacting with the ubiquitin machinery.

### FBXO28 regulates expression of MYC target genes

**Figure 3:**
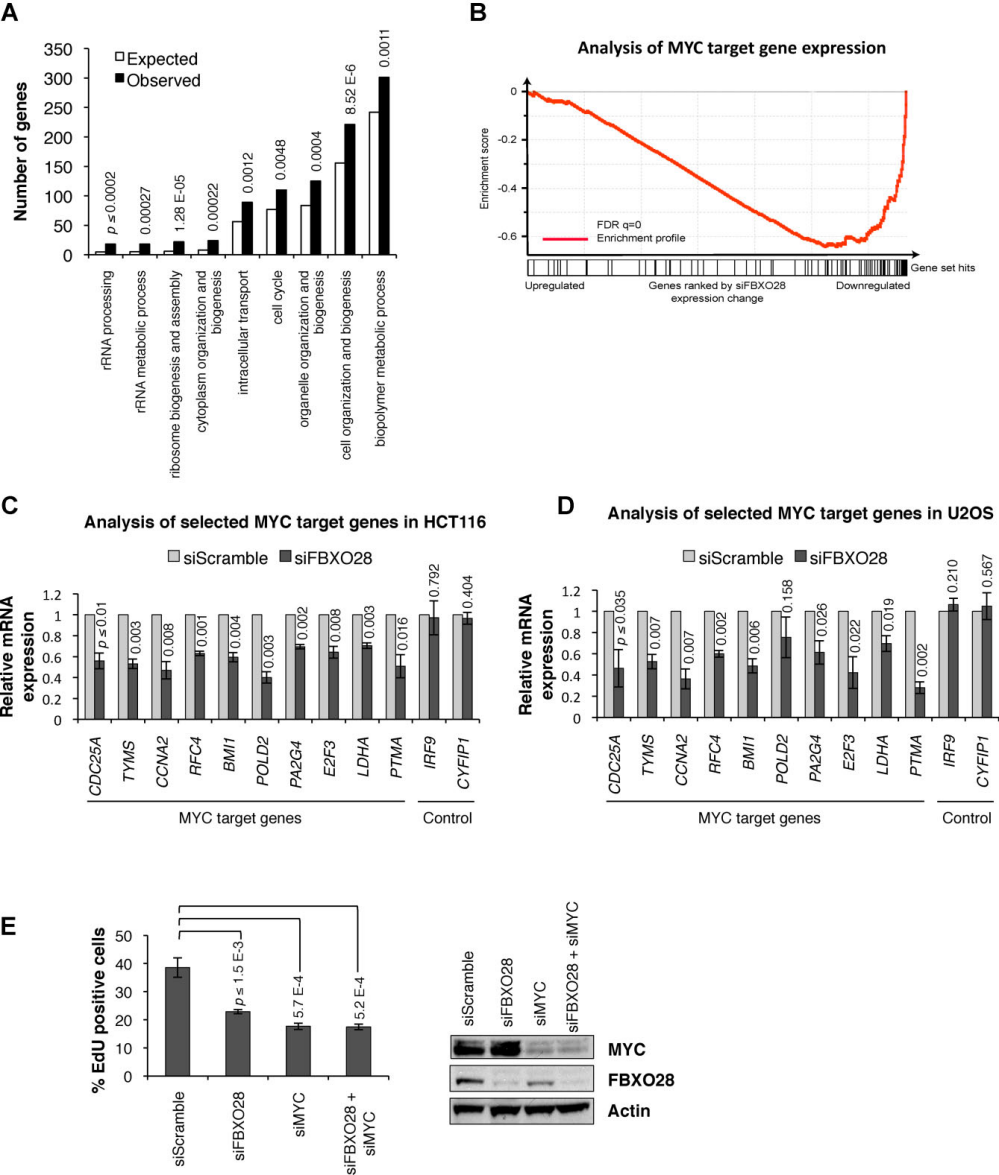
FBXO28 regulates expression of MYC target genes Analysis of microarray experiment documenting significantly enriched GO categories associated with knockdown of FBXO28 (36 h) in HCT116 cells. 71% (1200/1690) of the genes were downregulated at 36 h. Selected significantly enriched GO categories are represented by black bars and the number of expected occurrences are shown as white bars. *p*-values (shown above black bars) were adjusted for multiple hypothesis testing and calculated using Fisher's exact test.GSEA analysis of the effects of FBXO28 knockdown on MYC target gene expression in the microarray data set described in (A) shows that MYC target genes are significantly downregulated in response to FBXO28 depletion. The enrichment score (*y*-axis) reflects the degree to which the gene set ‘MYCUP PNAS VIA DAVID’ is overrepresented of the entire ranked list. Each solid bar at the *x*-axis represents one gene within the MYC target gene set and its corresponding distribution on the *x*-axis reflects the change in expression after 16 h FBXO28 depletion. Genes analysed = 80; *p* < 0.001 (permutation-based).qRT-PCR analysis documenting expression changes of selected MYC target genes (*n* = 10), and two selected non-MYC target genes as controls, in HCT116 cells andin U2OS cells transfected with scrambled or FBXO28 siRNA oligos. Expression values are relative to *β-ACTIN* gene expression. Columns represent the means of three independent experiments and bars are SEM. *p*-values (shown above bars) were calculated using that Student's *t*-test compared to siScramble for each gene.(Left) Flow cytometry experiments showing the percentage of EdU incorporation in HCT116 cells upon siRNA knockdown of FBXO28 or MYC alone, or together, for 48 h. (Right) Silencing efficiency for the siRNAs determined by WB using MYC and FBXO28 antibodies, and Actin as loading control. The graphs in (C–E) show means of three independent experiments and error bars represent SEM. The *p*-values depicted above the bars were determined by the Student's *t* test compared to siScramble control for each condition.

Since proliferation and transcriptional control are tightly linked processes regulated by ubiquitin ligases, we investigated the consequences of FBXO28 depletion on global gene expression by microarray analysis (Operon Biotechnologies). Strikingly, depletion of FBXO28 in HCT116 cells resulted in considerable changes in gene expression already after 16 h of siRNA transfection, and the majority of the differentially expressed genes were downregulated (88 and 71% at 16 and 36 h, respectively (GEO Accession no; GSE36112). The gene expression changes occured well before any signs of loss of proliferation (Supporting Information Fig S1A) and are therefore not likely a result of the proliferation arrest *per se*. Gene ontology (GO) analysis demonstrated that the most differentially expressed transcripts were genes involved in rRNA processing, ribosome biogenesis, cell cycle and metabolism (Fig 3A). This expression profile is highly reminiscent of transcriptional processes regulated by the oncoprotein/transcription factor MYC (Adhikary & Eilers, 2005; Eilers & Eisenman, 2008; Larsson & Henriksson, 2010; Meyer & Penn, 2008). Gene Set Enrichment Analysis (GSEA) (Subramanian et al, 2005) also suggested that MYC-activated genes were downregulated in response to FBXO28 depletion. Analyses using a defined gene set of known MYC targets (Fredlund et al, 2008) revealed their significant enrichment among the down-regulated genes at both 16 and 36 h (Fig 3B; Supporting Information Fig S3A). The reduction of a subset (*n* = 10) of the differentially expressed MYC-target genes was validated by qRT-PCR in FBXO28 depleted HCT116 and U2OS cells (Fig 3C and D; Supporting Information Fig S3B). In contrast, two control genes lacking MYC-specific E-boxes or association of MYC in their promoters according to publically available data (http://genome.ucsc.edu/ENCODE/analyses) did not exhibit reduced expression in response to FBXO28 depletion (Fig 3C and D). To address whether loss of proliferation in response to FBXO28 depletion depends on MYC, we silenced FBXO28 and MYC separately, or together in HCT116 cells. As shown in Fig 3E, MYC depletion reduced EdU incorporation somewhat stronger than FBXO28 knockdown. However, co-depletion of FBXO28 and MYC did not further reduce proliferation, suggesting that MYC and FBXO28 act in the same pathway. A similar interdependency was demonstrated when FBXO28 was depleted in MYC wild-type *versus* MYC-null rat cells (Supporting Information Fig S3C). In conclusion, knockdown of FBXO28 has profound effects on transcriptional processes regulated by MYC, including MYC target genes regulating macromolecular synthesis, metabolism and cell proliferation.

### Serine-344 phosphorylation-activated SCF^FBXO^^28^ promotes ubiquitylation but not degradation of MYC

**Figure 4:**
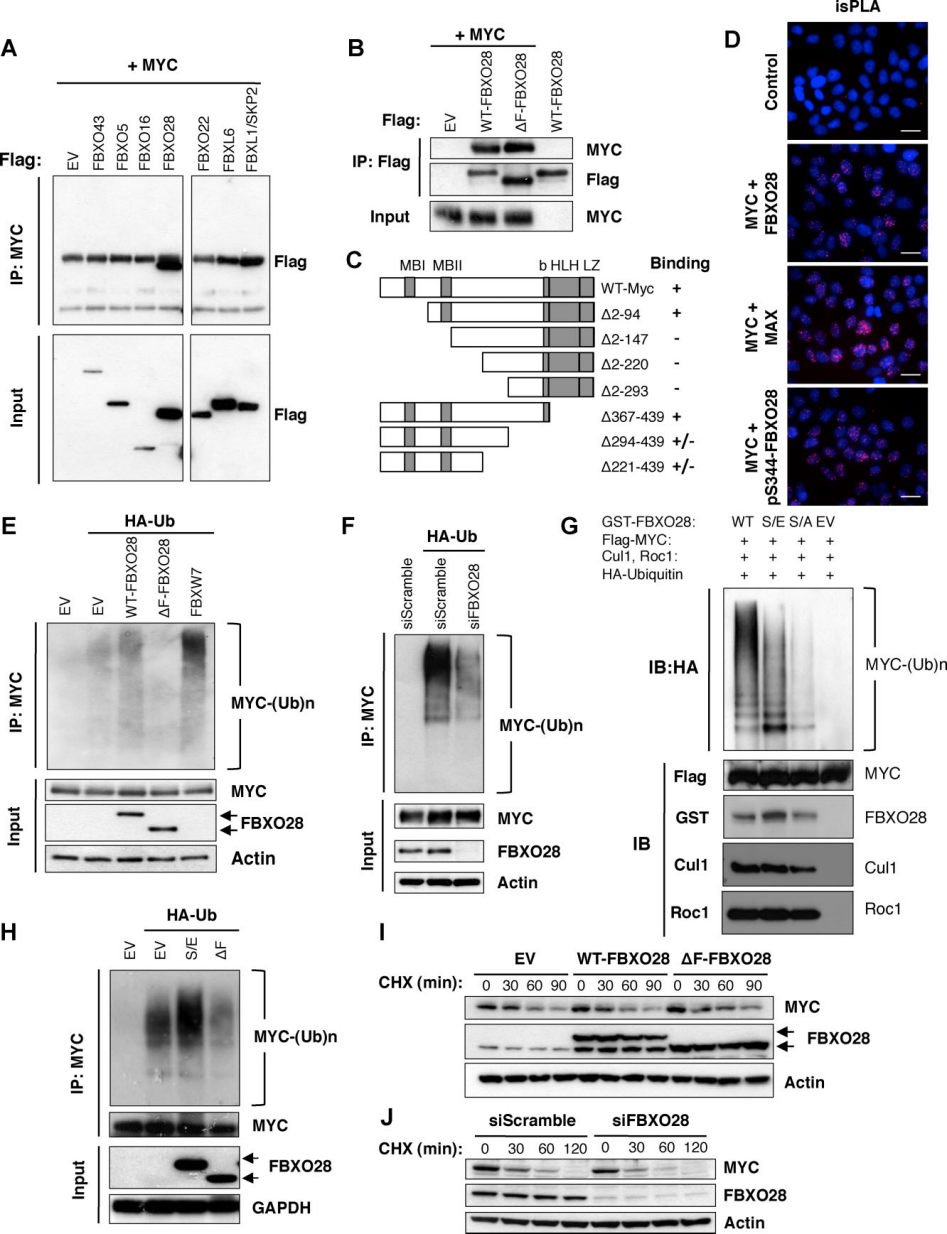
FBXO28 phosphorylation promotes MYC ubiquitylation but not degradation of MYC Extracts from HEK293 cells cotransfected with the indicated expression constructs were IP with MYC antibodies (N262) and IB with Flag antibody as indicated. Whole cell extracts were IB with Flag antibodies as input control. Blots were cropped vertically to exclude F-box proteins that are currently under study (unpublished data), with black lines delineating the boundaries between non-adjacent lanes.Extracts from HEK293 cells cotransfected with the indicated expression constructs were IP using Flag antibody and IB with MYC antibody (N262) as shown.Schematic diagram depicting the different *MYC* deletion constructs used to map the interaction with FBXO28 in Cos7 cells. The symbols to the right summarize the ability of each MYC mutant to interact with FBXO28. MBI/II, MYC Box I and II; b, basic region; HLH, Helix–loop–helix region; LZ, Leucine Zipper region.*In situ* proximity ligation assay (isPLA) to examine endogenous interactions between FBXO28 and MYC in HeLa cells. Top panel, negative control; next panels from top, MYC + FBXO28 antibodies, MYC + MAX antibodies, MYC + pS344-FBXO28 antibodies, respectively. Scale bars, 20 µm.*In vivo* ubiquitylation assay in HCT116 *FBXW7*^−/−^ cells cotransfected with the indicated expression constructs and treated with MG-132 for 4 h prior to harvesting followed by IP with MYC antibodies (N262). Ubiquitylation of endogenous MYC protein was detected by IB analysis with HA antibody. IB analysis of whole cell extracts with the indicated antibodies is shown.Ubiquitylation of exogenous MYC was analysed as in (E) in HCT116 *FBXW7*^−/−^ cells transfected with the indicated siRNAs and expression constructs.The indicated SCF^FBXO28^ complexes were used in an *in vitro* ubiquitylation reaction with FLAG-MYC in the presence of recombinant E1, E2 (UbcH5), HA-Ubiquitin and an energy regenerating system. MYC protein ubiquitylation was detected by IB analysis using anti-HA antibody. Formation of FBXO28-associated SCF complexes was assessed by Cul1 and Roc1 IB analysis, respectively. S/E: S344E-FBXO28, S/A: S344A-FBXO28.*In vivo* ubiquitylation assay in U2OS cells expressing doxycycline-inducible S344E-FBXO28 (S/E) and ΔF-FBXO28 (ΔF) constructs, transfected with HA-Ub and treated with MG-132 4 h prior to harvesting, followed by IP using MYC antibodies (N262). Ubiquitylation of endogenous MYC protein was detected by IB analysis with MYC (C33) antibody. IB analysis of whole cell extracts with the indicated antibodies is shown.HCT116 cells were transfected with the indicated expression constructs and MYC protein turnover was analysed by CHX-chase assay. Whole cell extracts were prepared at each time point following addition of CHX and MYC protein levels detected by IB.MYC protein turnover in HCT116 cells transfected with the indicated siRNAs was analysed as in (I). b-Actin levels were used as a general loading control in most experiments.

We next investigated whether FBXO28 interacts with MYC. As shown in Fig 4A and Supporting Information Fig S4A, MYC efficiently coprecipitated with FBXO28, and as expected also with SKP2 (FBXL1) (Kim et al, 2003; von der Lehr et al, 2003), but not with the other tested F-box proteins, including FBXO22, another of the top-ranked F-box genes from the siRNA screen presented in Fig 4A. Reciprocal coimmunoprecipitation of MYC verified the interaction with both WT- and ΔF-FBXO28 (Fig 4B), as expected since the F-box is usually not part of the substrate-recognition motif of F-box proteins (Carrano et al, 1999; Hart et al, 1999; Strohmaier et al, 2001). Mapping studies showed that deletion of the highly conserved MYC Box II (MBII) region, but not the MBI region, clearly reduced the interaction with FBXO28 (Fig 4C; Supporting Information Fig S4B). In contrast, deletion of the helix–loop–helix leucine zipper (HLH-LZ) motif in MYC did not affect binding, while deletion of a larger region of the C-terminus (Δ294–439) including the basic DNA binding region, MYC box IV and the NLS resulted in reduced affinity (Fig 4C; Supporting Information Fig S4B). We concluded that the MBII region and possibly motifs upstream of the HLH-LZ domain of MYC are important for the interaction with FBXO28. Finally, the nuclear interaction between total as well as phosphorylated endogenous FBXO28 with endogenous MYC protein was verified by *in situ* proximity ligation assay (isPLA) (Soderberg et al, 2006) (Fig 4D; Supporting Information Fig S4C).

Given these findings, we next investigated whether SCF^FBXO28^ ubiquitylates MYC. Ubiquitylation assays were performed in HCT116 *FBXW7*^−/−^ cells to avoid ubiquitylation of MYC by FBXW7, which is a major ubiquitin ligase for this protein (Welcker et al, 2004; Yada et al, 2004). As shown in Fig 4E, expression of WT-FBXO28 indeed promoted formation of high-molecular-weight MYC-ubiquitin conjugates in cells, whereas expression of ΔF-FBXO28 abolished MYC poly-ubiquitylation, consistent with dominant negative properties of F-box-deleted alleles (Δ*F*) (Strohmaier et al, 2001) that can bind substrates but not the SCF core (Fig 2G). Supporting these results, depletion of FBXO28 by siRNA heavily impaired MYC poly-ubiquitylation (Fig 4F). Finally, we found that FBXO28 stimulates poly-ubiquitylation of MYC *in vitro* using recombinant E1, E2, HA-ubiquitin and FBXO28 purified from cells (Supporting Information Fig S4D).

Since FBXO28 is a CDK1/2 substrate (Fig 2C), we hypothesized that phosphorylation of FBXO28 at S344 might influence SCF^FBXO28^ activity towards MYC. We thus performed *in vitro* ubiquitylation assays using immunopurified wild-type (SCF^WT-FBXO28^) or mutant (SCF^S344A-FBXO28^ and SCF^S344E-FBXO28^) SCF complexes. Strikingly, both the WT (SCF^WT-FBXO28^) and phospho-mimetic (SCF^S344E-FBXO28^) SCF complexes stimulated ubiquitylation of MYC *in vitro* and *in vivo* (Fig 4E, G and H), compared to the phospho-deficient SCF^S344A-FBXO28^ complex which was much less efficient in catalysing MYC ubiquitylation (Fig 4G). The reason for the differential ubiquitylation activity of these mutants was not due to impaired SCF assembly (Fig 4G and unpublished data). Consistent with these results, *in vivo* ubiquitylation assays demonstrated significant inhibitory effects by ΔF-FBXO28 on MYC ubiquitylation in S-phase, whereas only a minor inhibitory effect was observed in a population of cells in G1 phase (Supporting Information Fig S4E). Together, the data suggest that the intrinsic ubiquitin ligase activity of SCF^FBXO28^ is switched on by CDK1/2-mediated phosphorylation of FBXO28 at S344, thereby directing ubiquitylation of MYC as cells progress through S phase.

To investigate whether the interaction between FBXO28 and MYC was regulated during the cell cycle, we compared the binding of MYC to ΔF-FBXO28 (which is stable also in its non-phosphorylated state) expressed in either unsynchronized or synchronized cells. Although binding was detected in all cell cycle phases, a slightly reduced interaction was observed in the G1 and early S phases (Supporting Information Fig S4F). However, coimmunoprecipitation experiments using the phospho-deficient S344A mutant showed that phosphorylation at S344 was not required for FBXO28 to interact with MYC (Supporting Information Fig S4G), suggesting that the MYC-FBXO28 interaction is regulated through an S344-independent mechanism, possibly involving phosphorylation or other modifications at additional sites. Thus, the reduced ubiquitin conjugation activity of the SCF^S344A-FBXO28^ complex (Fig 4G) is likely not due to insufficient binding to MYC. Instead, CDK-mediated phosphorylation of FBXO28 at S344 appears to be critical for SCF^FBXO28^ ubiquitin conjugation activity and poly-ubiquitylation of MYC.

To test whether ubiquitylation of MYC by FBXO28 stimulates its degradation, we measured the steady-state levels and stability of MYC protein by CHX chase and immunoblot analysis. As shown in Fig 4I and Supporting Information Fig S4H, expression of either WT- or ΔF-FBXO28, did not significantly affect MYC protein levels or stability in HCT116 cells. In addition, depletion of FBXO28 by siRNA did not change the half-life of endogenous MYC protein (Fig 4J and Supporting Information Fig S4H). Similar results were found in HCT116 *FBXW7*^−/−^ cells indicating that FBXO28 does not influence MYC turnover (Supporting Information Fig S4I and J). Taken together, we conclude that SCF^FBXO28^ is a *bona fide* ubiquitin ligase promoting MYC poly-ubiquitylation in response to CDK-mediated S344 phosphorylation without inducing proteolytic degradation of MYC.

### SCF^FBXO^^28^ ubiquitin ligase activity promotes MYC-driven transcription by stimulating MYC-p300 interactions at target promoters

**Figure 5:**
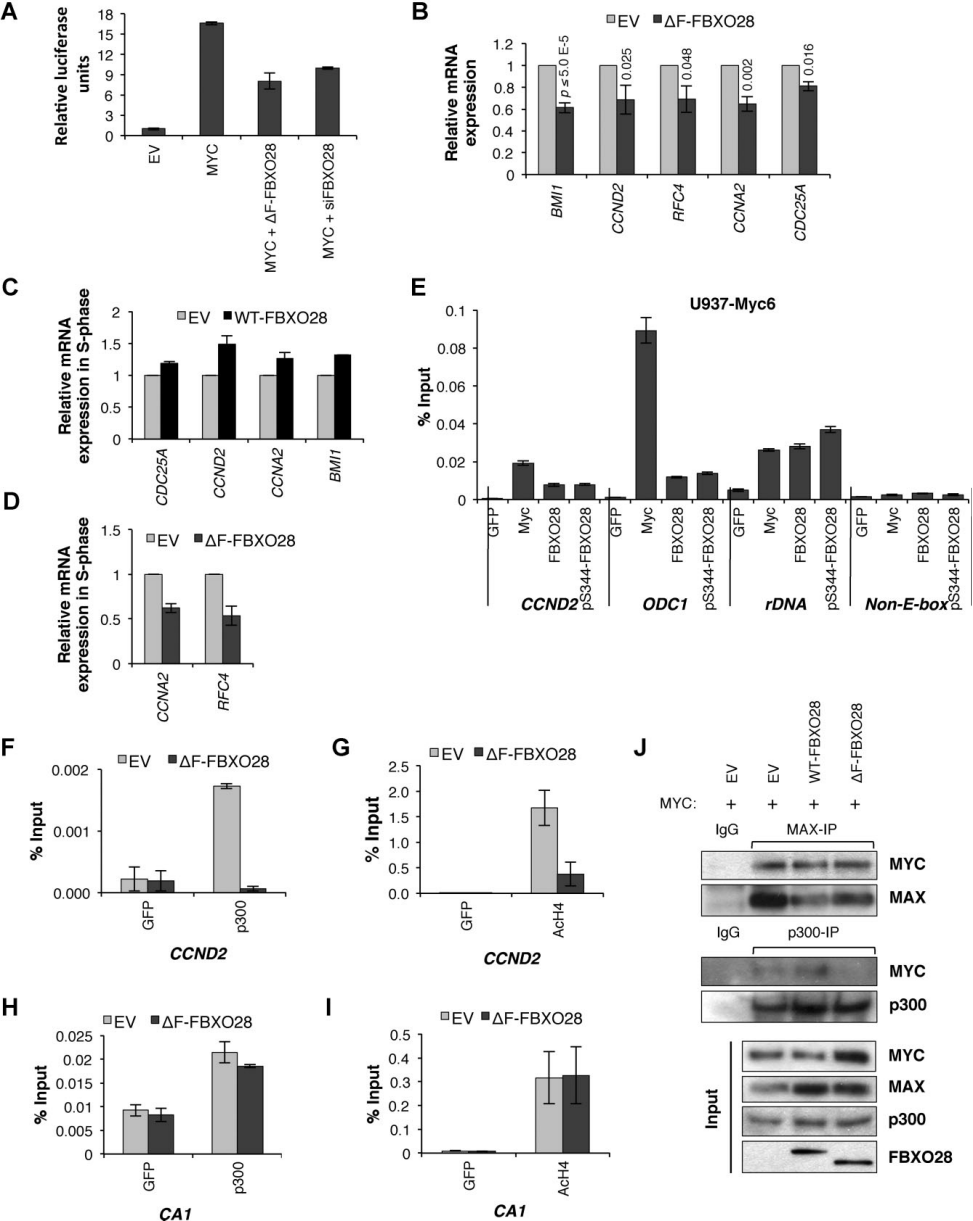
SCF^FBXO28^ ubiquitin ligase activity promotes MYC-driven transcription by stimulating MYC-p300 interactions at target promoters **A.**Promoter/luciferase reporter assay in HeLa cells transfected with the M4mintk-Luc reporter in combination with the indicated expression constructs. Luciferase activity was normalized to β-Galactosidase control. The data is representative of three experiments and bars represent the mean ± SEM for three measurements.**B.**qRT-PCR analysis documenting the expression levels of MYC target genes upon doxycycline-induced ΔF-FBXO28 expression in asynchronously proliferating U2OS cells. The graphs depict means of three independent experiments; bars represent SEM, with *p*-values compared to EV for each gene shown above the bars (Student's *t*-test).**C.**qRT-PCR expression analysis of the indicated MYC target genes in U2OS cells released into S-phase (4 h) following synchronization at the G1/S border by double thymidine treatment. WT-FBXO28 expression was induced by doxycycline treatment prior to synchronization. Values were normalized to *β-ACTIN* and data represent the average of two independent experiments**D.**qRT-PCR of selected MYC target genes in U2OS cells expressing doxycycline-inducible ΔF-FBXO28. Experiments and analyses were performed as in (C).**E.**Q-ChIP assay in U-937 cells with the indicated antibodies at the depicted gene promoters. GFP antibody was used as a negative control in all experiments. Enrichment was quantified by q-PCR, normalized to input DNA and presented as the average of three determinations. Error bars represent SEM. Data is representative of two or three independent experiments.**F–I.**Q-ChIP assays measuring the associating of p300 (F and H) or acetylated histone H4 (G and I) at the *CCND2* MYC target gene promoter (F and G) or the carbonic anhydrase I (*CA1*) non-MYC target gene promoter (H and I), upon doxycycline-induced ΔF-FBXO28 expression in U2OS cells. Error bars represent SEM of the average of two independent experiments, normalized to % input.**J.**Analysis of interaction between MYC-MAX (upper panel) and MYC-p300 (middle panel) upon doxycycline-induced expression of EV, WT- or ΔF-FBXO28 in U2OS cells transfected with a MYC expression plasmid. Cells were treated with doxycycline for 24 h and lysates were subjected to coimmunoprecipitation analysis with the indicated antibodies. Ten percent of each lysate was used as input controls (lower panel).

To assess whether SCF^FBXO28^ ligase activity is required for MYC-induced transcription, we first performed transient promoter/luciferase reporter assays using a construct containing a minimal promoter with multiple MYC binding sites. As shown in Fig 5A, overexpression of the dominant-negative ΔF-FBXO28 mutant or siRNA-mediated FBXO28 depletion in HeLa cells attenuated MYC-induced luciferase reporter activity. In contrast, the salt-induced kinase (SIK) gene promoter/luciferase reporter, which does not contain MYC binding sites, and a minimal promoter/reporter containing four SMAD-binding sites did not respond to either MYC or ΔF-FBXO28 overexpression nor to FBXO28 depletion, further supporting the preferential requirement of FBXO28 for specific transactivation of MYC target genes (Supporting inforation Fig S5A and B). Furthermore, doxycycline (Dox)-induced expression of ΔF-FBXO28 in asynchronously proliferating U2OS cells significantly reduced the expression of several endogenous MYC target genes (Fig 5B).

Considering that phosphorylation of FBXO28 at S344 occurs during progression through the S and G2/M phases of the cell cycle (Fig 2B) and is required for ubiquitylation of MYC (Fig 4G and H), we next analysed expression of MYC target genes upon expression of WT-FBXO28 and ΔF-FBXO28 in S-phase cells. Dox-regulated U2OS cells were synchronized at the G1/S border by double thymidine treatment followed by release into S-phase. As shown in Fig 5C and D, WT-FBXO28 enhanced expression of several endogenous MYC target genes and ΔF-FBXO28 clearly attenuated transactivation of those genes, compared to control cells. Similar results were observed in a synchronized population of HeLa cells released into S-phase following depletion of FBXO28 using siRNA (Supporting Information Fig S5C).

To exclude that FBXO28 depletion affected cell cycle genes in general, we cross-referenced our microarray data from siFBXO28 treated cells (Fig 3 and Supportive Information Fig S3) with publically available, cell-cycle-specific, gene expression and ChIP data (Whitfield et al, 2002) and http://genome.ucsc.edu/ENCODE/analyses). MYC was found to bind around 3/4 of the defined cell cycle-regulated genes (Supportive Information Fig S5D). Depletion of FBXO28 significantly downregulated a subpopulation of the MYC target genes (in particular in S, G2 and M-phases), while non-MYC target genes seemed less affected (2 non-MYC *versus* 33 MYC target genes, *p* = 0.03). This demonstrates that FBXO28 knockdown preferentially affects MYC target genes and does not lead to a general downregulation of gene expression in S-G2 phases of the cell cycle.

We next addressed whether FBXO28 plays a more direct role in MYC-mediated transcription and associates with MYC binding sequences in MYC target genes. As depicted in Fig 5E, quantitative chromatin IP (Q-ChIP) assays using U-937 cells demonstrated that FBXO28 (and phosphorylated FBXO28) was specifically enriched within the E-box regions of the *ODC1*, *CYCLIN D2* (*CCND2*) and *rDNA* gene promoters, to which MYC binds together with its obligatory partner MAX, but not at the *FAS* ligand gene promoter, which lacks E-box motifs. Significant enrichment of FBXO28 at these promoters as well as additional MYC targets, including the *PTMA*, *BMI1* and *RFC4* gene promoters, was also detected in U2OS and HCT116 cells (Supporting Information Fig S5E and F). As would be expected for a cofactor that binds DNA indirectly through transient interactions with DNA-binding transcription factors, FBXO28 enrichment at target promoters was generally weaker as compared to MYC (or MAX, Fig 5E; Supporting Information Fig S5E and F and unpublished data).

Since the MYC/MAX transcriptional complex regulates transcription, at least in part, by influencing the local chromatin structure at promoters (Adhikary & Eilers, 2005; Eilers & Eisenman, 2008; Larsson & Henriksson, 2010; Meyer & Penn, 2008), which is important both for initiation and elongation of transcription, we reasoned that ubiquitylation of MYC by SCF^FBXO28^ might be required to support specific MYC-coactivator interactions of relevance for chromatin regulation, such as the histone acetyltransferase (HAT) p300 which has previously been shown to interact with MYC (Adhikary et al, 2005; Faiola et al, 2005; Vervoorts et al, 2003). Indeed, the enrichment of HAT p300 and of acetylated histone H4 at the *CCND2* and *BMI1* gene promoters was severely impaired upon expression of ΔF-FBXO28 in U2OS cells (Fig 5F and G; Supporting Information Fig S5G and H), while MYC and MAX binding was not affected (Supporting Information Fig S5I). In contrast, neither association of p300 nor histone H4 acetylation was affected by expression of ΔF-FBXO28 at the *CA-1* promoter, which is not bound by MYC (Fig 5H and I). These data are consistent with a role of SCF^FBXO28^ ubiquitin ligase activity in recruitment of p300 to MYC at target gene promoters, but not to promoters in general. To test this hypothesis, we assessed p300-MYC complex formation by coimmunoprecipitation analysis in cells expressing WT-FBXO28 or ΔF-FBXO28. As shown in Fig 5J, neither WT-FBXO28 nor ΔF-FBXO28 affected the binding of MYC to MAX. However, ΔF-FBXO28 expression abrogated the interaction between MYC and p300, as compared to WT-FBXO28 expression (Fig 5J).

To investigate what lysines in MYC are targeted for ubiquitylation by FBXO28 and possibly of importance for regulating the MYC-p300 interaction, we examined ubiquitylation of specific C-terminal MYC deletion constructs upon expression of ΔF-FBXO28. We found that while ΔF-FBXO28 expression reduced ubiquitylation of WT-MYC and a C-terminal MYC deletion mutant (Δ367–439), it did not significantly affect the ubiquitylation status of MYC truncations lacking amino acids 294–439 (Supporting Information Fig S5J). Since ubiquitylation on one or more of six lysine residues within this region was previously reported to promote ubiquitin-mediated complex formation between MYC and p300 (Adhikary et al, 2005), we next tested if these particular lysines (K6R) are potential targets for the SCF^FBXO28^ ubiquitin ligase. Indeed, we found that while expression of ΔF-FBXO28 reduced ubiquitylation of full-length MYC and a MYC mutant where lysines at positions 51 and 52 (NK2R) had been replaced with arginines, it did not attenuate ubiquitylation of the K6R mutant (Supporting Information Fig S5K). Thus, SCF^FBXO28^ promotes ubiquitylation on specific lysine residues within the 294–367 region of MYC, which is known to be involved in ubiquitin-mediated MYC-p300 interaction. Therefore, these data indicate that SCF^FBXO28^-mediated MYC ubiquitylation promotes MYC-driven transcription by facilitating recruitment of p300 and possibly other cofactors to target promoters.

### SCF^FBXO^^28^ activity promotes MYC-induced tumourigenesis

**Figure 6:**
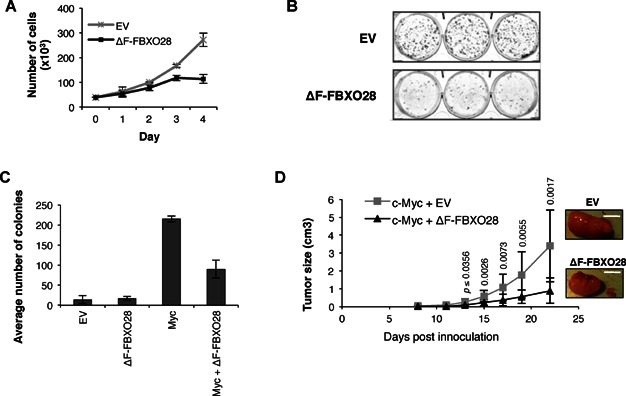
SCF^FBXO28^ Activity promotes MYC-Induced Tumourigenesis Cell proliferation analysis documenting loss of proliferation upon expression of ΔF-FBXO28 as compared to EV in U2OS cells. Cells were treated with doxycycline for 48 h, counted and replated at equal numbers in the presence of doxycycline, as indicated. The data represent the average ± SEM of three cell counts for each time point.Colony formation assays documenting the effect of overexpression of ΔF-FBXO28 in U2OS cells. Equal numbers of cells were transfected with the indicated expression constructs, plated in triplicates and selected with G418 for 10 days. Colonies were fixed and stained with Giemsa.Soft agar colony formation assays using *P53*^−/−^ MEFs transduced with the indicated retroviruses. Transformation was scored by counting colonies of relatively the same size across all conditions. Representative data of the mean from three counts ± SEM for one of three independent experiments.Tumour growth in immunodeficient mice injected with MYC-transformed *P53*^−/−^ MEFs stably transduced with either EV or ΔF-FBXO28. Tumour growth was measured using a caliper at the indicated times after injection as indicated. Right panel: Representative image of tumours. Scale bar, 10 mm. Graph presents the average tumour size of five mice per group, with two tumours (one on each flank); error bars indicate SEM. *p*-values between the groups, shown above error bars, were calculated for each timepoint using one-way ANOVA.

To explore the physiological importance of SCF^FBXO28^ E3 ligase activity, we measured the effect of ΔF-FBXO28 on tumour cell proliferation and MYC-induced tumourigenesis. As expected, Dox-induced expression of ΔF-FBXO28 in U2OS cells significantly reduced cell numbers after 2–3 days (Fig 6A) similar to FBXO28 depletion (Fig 1D). Ectopic expression of ΔF-FBXO28 also significantly reduced the ability of these cells to grow as colonies on plastic (Fig 6B). To directly test whether FBXO28 activity is required for MYC-induced transformation, we engineered *P53*^−/−^ immortalized mouse embryonic fibroblasts (MEFs) (which are known to be efficiently transformed by MYC (Ecker et al, 2009), with retroviruses encoding MYC and ΔF-FBXO28, or the individual genes, and performed transformation assays. Notably, ΔF-FBXO28 expression significantly reduced MYC-induced foci formation (unpublished data) and colony formation in soft agar (Fig 6C). A similar reduction in MYC-induced colonies was observed when FBXO28 was depleted in MYC-transduced *P53*^−/−^ MEFs (Supporting Information Fig S6A). ΔF-FBXO28 expression did not significantly affect the number of RAS-induced colonies in these cells (unpublished data), further supporting the notion that FBXO28 activity is specifically linked to MYC-driven transformation. To determine whether FBXO28 activity is important for MYC-driven tumour growth, MYC-transformed MEFs were transduced with ΔF-FBXO28 retroviruses, and pools of puromycin-resistant MEFs expressing ΔF-FBXO28 were subsequently injected into immunodeficient mice. As shown in Fig 6D, these tumours grew significantly slower, as compared to the control mice injected with MEFs expressing MYC alone. The ability of the tumours to retain expression of ΔF-FBXO28 was confirmed by immunoblotting (Supporting Information Fig S6B). Thus, expression of dominant-negative ΔF-FBXO28 was sufficient to suppress MYC-induced transformation *in vitro* and tumour growth *in vivo*. Together, these data strongly imply that SCF^FBXO28^ ubiquitin ligase activity is required to support MYC-dependent transcription and tumourigenicity.

### High expression of FBXO28 in human breast cancer correlates with activation of a gene subset targeted by the MYC/p300 pathway

**Figure 7:**
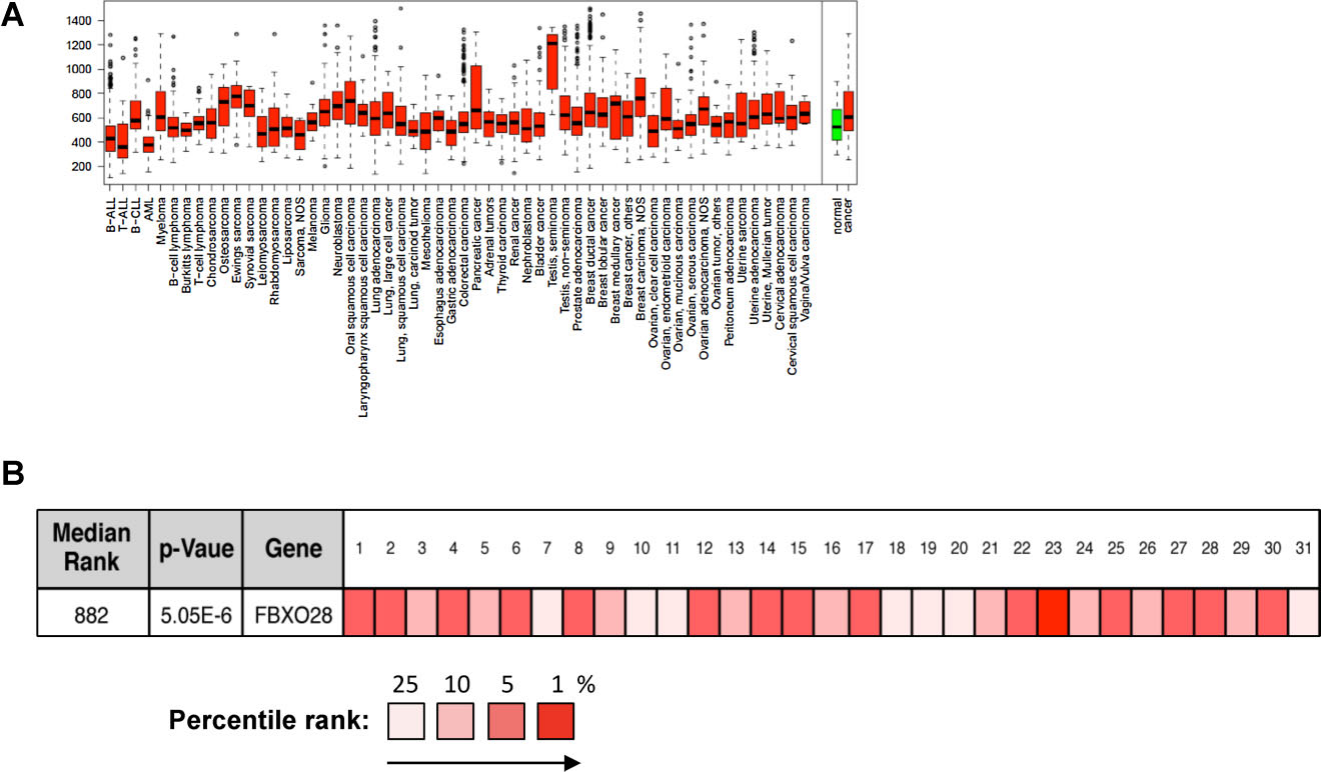
High Expression of FBXO28 in human breast cancer correlates with MYC pathway activity Box plot analysis of *FBXO28* expression levels across a variety of cancer tissues. The box refers to the quartile distribution (25–75%) range, with the median shown as a black horizontal line. In addition, the 95% range and individual outlier samples are shown.High expression of *FBXO28* in human breast cancer. Summary of 31 different microarray-based breast cancer studies retrieved from the Oncomine database (OncomineTM, Compendia Bioscience, Ann Arbor, MI, https://www.oncomine.org/) is shown. Studies include tumour tissue *versus* normal tissue, high grade *versus* low-grade tumour, as well as recurrence and survival rates (numbered and referenced in Table S2 of Supporting Information). The diagram displays the percentile rank for FBXO28 across each of the analyses by the degree of colour saturation of the box. The most highly saturated boxes denote that the gene rank is in the top 1 percentile for that analysis; medium saturation in the top 5 percentile, and pale in the top 10 percentile. Red denotes over-expression. The differential expression *p*-value for the median-ranked analysis was calculated using an independent, two-sample, one-tailed Welch's *t*-test (https://www.oncomine.org/).

The strong association between FBXO28, MYC and tumourigenesis in the above model systems prompted us to further evaluate if this relationship is important also in the context of primary human tumour cells. Analyses of *FBXO28* mRNA expression in various malignant tissues using the *in silico* transcriptomics database of the GeneSapiens System (http://www.genesapiens.org) demonstrated elevated levels in several different tumour types, including breast cancer, gynecological tumours, sarcomas and neuronal tumours (Fig 7A). Indeed, searching the Oncomine database (Rhodes et al, 2004) verified that *FBXO28* expression was significantly higher in primary breast cancers relative to normal breast tissue in multiple expression profiling studies (Fig 7B and Supporting Information Table S2). To directly evaluate the potential association between *FBXO28* expression and the expression of MYC target genes in breast cancer, we extracted gene expression data representing 327 clinical breast cancer specimens ((Loi et al, 2007); GSE6532) and identified 102 genes highly related to *FBXO28* expression. Of these genes, 35 were positively correlated and 67 were negatively correlated to *FBXO28* expression (Supporting information Tables S3 and S4). Detailed analyses of the gene expression network using publically-available ChIP-seq data (http://genome.ucsc.edu/ENCODE/analyses) revealed that there was a highly significant overrepresentation (*p* = 3.8E−15) of MYC binding at the promoter regions among the positively correlated genes (29 of the 35 positively correlated genes), in relation to the negatively correlated genes (6 of the 67 genes) (Supporting Information Tables S3 and S4). Finally, using available p300 ChIP-seq data we also found a strong trend (*p* = 0.055) towards coassociation of p300 with MYC at overlapping peaks at the promoters of positively correlated genes (66%), whereas MYC was less frequently associated with p300 at negatively correlated genes (25%) (Supporting Information Tables S3 and S4), in line with the presented molecular data (Fig 5; Supporting Information Fig S5). We conclude that FBXO28 expression is positively correlated with increased expression of a subset of genes that are preferentially targeted by MYC in association with p300 in human breast cancer.

### Expression and phosphorylation of FBXO28 is associated with poor prognosis and worse survival in human breast cancer

**Figure 8:**
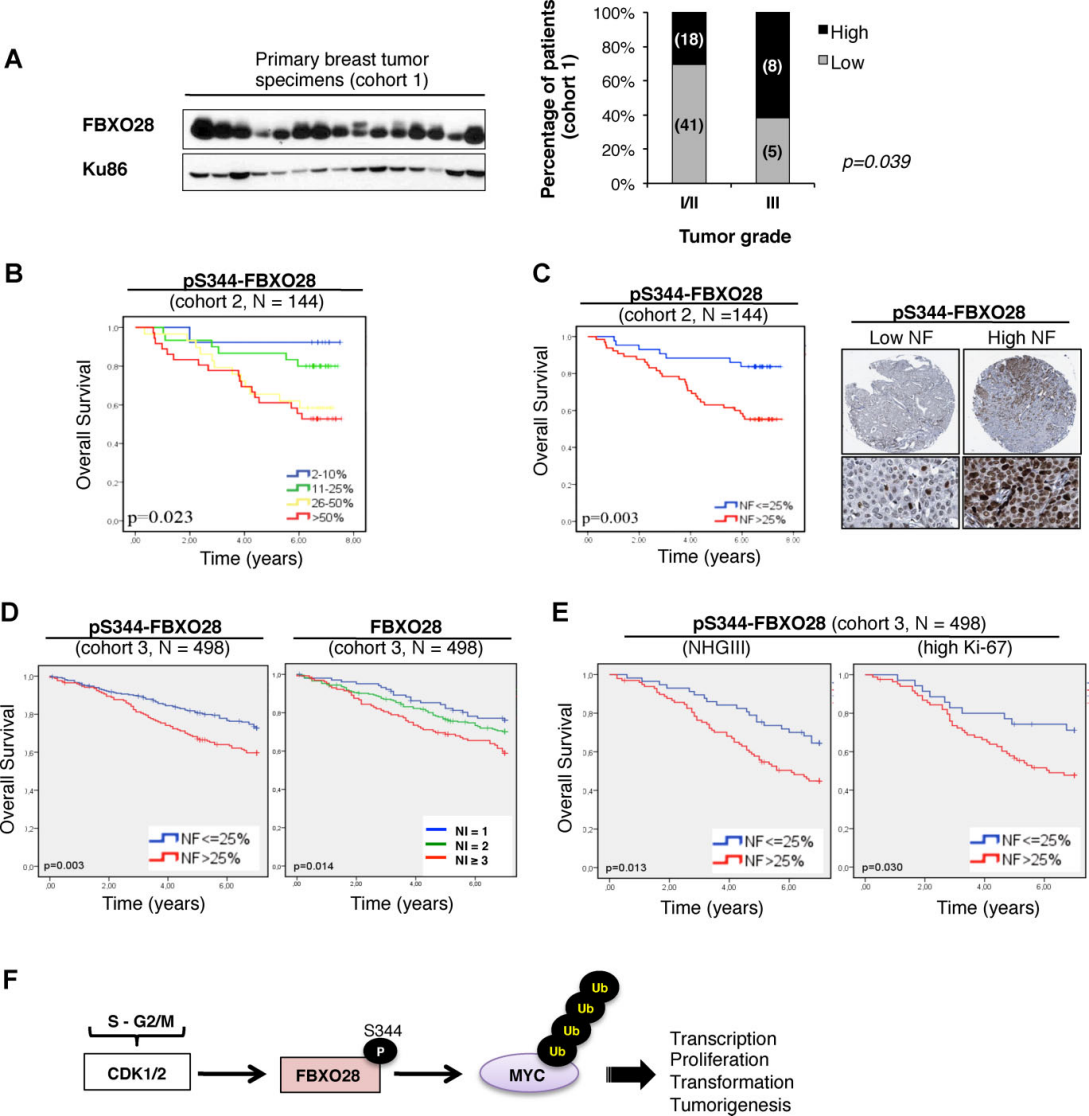
Expression and phosphorylation of FBXO28 are associated with poor outcome in human breast cancer Left panel: IB analysis of FBXO28 protein expression in a subset of primary breast tumour specimens from cohort 1. Right panel: Semi-quantitative analysis of FBXO28 protein levels and its relationship with tumour grade was calculated after normalization against an input control. Black bars represent the percentage of patients with high FBXO28 levels and grey bars denote the percentage of patients with low FBXO28 levels, in each respective patient group (NHGI/II *vs*. NHGIII). *p* = 0.039 calculated using one-tailed Fisher's exact test. (*n* = 72, cohort 1).Kaplan–Meier plot of OS in breast cancer patients (*n* = 144, cohort 2) stratified according to the fraction of cells staining positive for phosphorylated FBXO28 in the nucleus. A log rank test was used to show differences between patient groups with different nuclear fractions (NF) (*p* *=* 0.023). Immunohistochemistry was performed using anti-S344-FBXO28 antibody.Left panel: A log rank test was used to show differences between patient groups with high (>25%) and low (<25%) NF of phosphorylated FBXO28 and OS (cohort 2) (*p* *=* 0.003). Right panel: Representative images of breast carcinoma TMA cores with a low (<25%) and a high (>25%) NF. Analysis was performed as in (B).Kaplan–Meier plots of OS in breast cancer patients (*n* = 498, cohort 3) stratified according to the nuclear fraction (NF; 25% cut-off) of phosphorylated FBXO28 (left panel, *p* *=* 0.003) or the nuclear staining intensity (NI; 1 = low, 2 = moderate, ≥3 high) of FBXO28 protein (right panel, *p* *=* 0.014). Immunohistochemistry analysis was performed using the anti-pS344-FBXO28, and the FBXO28 antibody detecting total FBXO28 protein.Kaplan–Meier plots of OS in breast cancer patients (*n* = 498, cohort 3) classified as high-grade (NHGIII) (left panel, *p* *=* 0.013), and high-Ki67 (right panel, *p* *=* 0.030) tumours. Patients were stratified according to the NF of FBXO28 phosphorylation. A log-rank test was used to show differences between groups using 25% NF as a cut-off. NHGIII, Nottingham histological grade III.FBXO28 is phosphorylated by CDK1/2 during progression through the S- and G2-phases of the cell cycle. Phosphorylation of FBXO28 at serine 344 activates SCF^FBXO28^, promoting non-proteolytic ubiquitylation of MYC, interaction with the transcriptional cofactor p300 and transcription of MYC target genes resulting in increased transcription, proliferation, transformation and tumourigenesis.

In light of the findings described above, we further explored the potential involvement of FBXO28 in breast tumourigenesis. FBXO28 immunoblot analysis in a cohort of primary breast tumour specimens (cohort 1, *n* = 72), demonstrated a statistically significant correlation between high FBXO28 protein levels and poorly differentiated breast tumours (*p* = 0.039) (Fig 8A). We also observed substantial variation in the levels of phosphorylated FBXO28 in these breast tumours (Supporting Information Fig S7A). We next evaluated FBXO28 phosphorylation by immunohistochemistry (IHC) analysis performed on tissue microarrays (TMAs) the pS344-FBXO28 specific antibody in a second and independent cohort of 144 breast tumour specimens (cohort 2). Strikingly, a statistically significant correlation was found between a high nuclear fraction (NF) of pS344-FBXO28 and several adverse clinicopathological characteristics, including tumour size, poorly differentiated and ER-negative tumours (Supporting Information Table S5A). This suggests that expression and phosphorylation of FBXO28 may be linked to clinical outcome in breast cancer. Indeed, the NF of phosphorylated FBXO28 clearly separated breast tumours according to OS, as evaluated by log-rank analysis of Kaplan–Meier curves (Fig 8B). When the FBXO28 data were dichotomized into NF < 25% *versus* NF > 25%, a significant association between high NF of pS344-FBXO28 and OS (*p* = 0.003), breast cancer-specific survival (BCSS) (*p* = 0.011) and recurrence free survival (RFS) (*p* = 0.016) was demonstrated (Fig 8C; Supporting Information Fig S7B). Using Cox modeling, we found a strong association between a high NF of FBXO28 and decreased OS (*p* = 0.005), BCSS (*p* = 0.020) and RFS (*p* = 0.024) (Supporting Information Table S5B). Importantly, when analysed by multivariate analysis, the NF of FBXO28 retained its prognostic significance as an independent predictor of poor OS in breast cancer (HR 3.19 [1.22–8.33], *p* = 0.018) (Supporting Information Table S5C).

To validate and extend these results, we performed TMA analysis of FBXO28 expression and phosphorylation in an additional large and independent breast cancer cohort (cohort 3, *n* = 498). In this cohort, a high NF of phosphorylated FBXO28 was also associated with aggressive disease (Supporting Information Table S6A), worse OS (Fig 8D), BCSS and RFS (Supporting Information Fig S7C). In addition, when total FBXO28 protein expression levels were evaluated according to nuclear staining intensity (NI), Kaplan–Meier analysis showed that increased levels of FBXO28 protein were associated with decreased OS (Fig 8D), BCSS and RFS (Supporting Information Fig S7D). Importantly, both FBXO28 expression (NI) and phosphorylation (NF) were found to be independent predictors of worse outcome by multivariate analysis also in this cohort of breast cancer patients (Supporting Information Table S6B and C). Remarkably, we found that FBXO28 phosphorylation was associated with worse survival also in specific patient subgroups with particularly adverse prognosis, including the most highly proliferative (measured by Ki-67 analysis) and poorly differentiated (high-grade) tumours (Fig 8E) and interestingly, also in Luminal A tumours, which are generally characterized by a uniformly low proliferation status (Supporting Information Fig S7E). Notably, these data indicate that expression and phosphorylation of FBXO28 do not simply reflect the proliferative status of the tumours; however, we cannot exclude the possibility that FBXO28 might regulate other aspects of poor outcome (such as invasion), irrespective of its association with high CDK activity and poor prognosis. Nevertheless, these data underscore the clinical relevance and prognostic impact of FBXO28 in human breast cancer.

## DISCUSSION

In this study, we provide the first clues as to the function of the F-box protein, FBXO28. Initially, FBXO28 was identified in a high-throughput, unbiased loss-of-function screen for genes required for efficient tumour cell proliferation (Fig 1). In line with its role as a regulator of tumour cell proliferation, we discovered that cells depleted of FBXO28 downregulated genes activated by the transcription factor MYC (Fig 3). This work further establishes that FBXO28 is a *bona fide* ubiquitin ligase for MYC and also demonstrates a novel posttranslational regulatory network in which CDK-mediated phosphorylation of FBXO28 promotes non-proteolytic MYC ubiquitylation (Fig 4) and recruitment of the cofactor p300 to MYC target gene promoters (Fig 5). The function of FBXO28 in regulation of MYC activity is also supported by the findings that depletion or functional inactivation of FBXO28 attenuates MYC-induced transformation *in vitro* and MYC-driven tumour growth *in vivo* (Fig 6). In line with the molecular data, we found an association between *FBXO28* expression and the expression of MYC target genes in human breast cancer (Table S3) and importantly, that elevated levels of FBXO28 protein expression and phosphorylation are strong and independent predictors of poor outcome in breast cancer (Fig 8).

To date, apart from FBXO28, at least five E3 ubiquitin ligases, SKP2, FBXW7, HECTH9/HUWE1, TRUSS and bTRCP have been shown to regulate MYC (Adhikary et al, 2005; Choi et al, 2010; Kim et al, 2003; Popov et al, 2010; Thomas & Tansey, 2011; von der Lehr et al, 2003; Welcker et al, 2004; Yada et al, 2004; Zhao et al, 2008). The reason for this apparent redundancy is not fully understood, but a likely cause is that powerful oncoproteins like MYC need to be maintained under rigorous control, and it is conceivable that different ligases regulate MYC activity and/or degradation in response to various specific signals. The primary role of a subset of the aforementioned E3 ligases seems solely to act as negative (FBXW7 and TRUSS) or positive (bTRCP) regulators of MYC protein stability by conjugating different types of poly-ubiquitin chains (Choi et al, 2010; Popov et al, 2010; Welcker et al, 2004; Yada et al, 2004). In contrast, FBXO28 belongs to another category of ubiquitin ligases, which also includes SKP2 and HECTH9/HUWE1, that play a role in promoting MYC activity (Adhikary et al, 2005; Kim et al, 2003; Thomas & Tansey, 2011; von der Lehr et al, 2003), although HECTH9/HUWE1 has also been reported to stimulate the degradation of MYCN (Zhao et al, 2008). One of the prominent features of the SCF^FBXO28^ ubiquitin ligase is that it needs to be phosphorylated on serine 344 by cyclin A/CDK2 or cyclin B/CDK1, but not cyclin E/CDK2, in order to exert its ubiquitin ligase activity towards MYC, demonstrating a particular need for SCF^FBXO28^ in coordinating the activity of distinct CDKs with MYC function during the cell cycle. We suggest a model where CDK1/2-mediated phosphorylation of FBXO28 triggers the activation of the SCF^FBXO28^ ligase and subsequent ubiquitylation of MYC at the S- and G2/M phases of the cell cycle. This promotes the recruitment of the cofactor p300 thereby stimulating transcriptional activation of a subset of MYC target genes critical for the continued progression through the cell cycle (Fig 8F). In comparison, SKP2- and HECTH9-mediated ubiquitylation of MYC has been reported to play a more prominent role in the G1 or the G1/S phase transition (Adhikary et al, 2005; Kim et al, 2003; von der Lehr et al, 2003). FBXO28 and HECTH9/HUWE1 seem to operate in a similar manner by targeting specific lysines in the C-terminal part of MYC, in the case of HECTH9 through lysine 63-linked ubiquitin chains, which enhance recruitment of p300 to MYC (Adhikary et al, 2005). Since FBXO28 promotes non-proteolytic ubiquitylation of MYC, the conjugated ubiquitin chains are likely to be non-lysine 48-linked as in the case for HECTH9, or possibly heterotypic ubiquitin chains as reported for bTRCP (Popov et al, 2010). It is therefore conceivable that SKP2, HECTH9 and FBXO28 promote MYC-driven transcription of overlapping or distinct subsets of MYC target genes in different phases of the cell cycle and/or in response to different signals. These are important issues that need to be addressed in future studies.

Recent findings suggest that MYC enhances global expression from E-box-containing promoters in an active or poised state (Lin et al, 2012; Nie et al, 2012). However, it is also clear that MYC has different effects on different genes, for instance due to the concentration of E-boxes, or as a result of enhancer invasion at high enough MYC levels, and collaboration with other transcription factors or cofactors. Our present finding of cooperativity between MYC and FBXO28 exemplifies an additional level of complexity to MYC-regulated transcription by demonstrating that cofactors like FBXO28 seem to regulate a subset of MYC target genes (Fig 3B, Supporting Information Figs S3A and S5D).

We found that CDK-mediated phosphorylation at serine 344 stimulates SCF^FBXO28^ activity towards MYC. This type of regulation, phosphorylation of the ubiquitin ligase rather than the substrate, resembles the regulation of the multisubunit E3 ligase APC (anaphase-promoting complex) by CDKs (Rahal & Amon, 2008). The regulation of SCF activity is poorly understood, but mechanism(s) involving dimerization and auto-ubiquitylation or interaction with specific cofactors have been proposed (Cope & Deshaies, 2006). For instance, CDK-mediated phosphorylation affects SKP2 stability, and allows its expression in mid-G1 phase by protecting it from degradation (Rodier et al, 2008). Interestingly, we found that phosphorylation of FBXO28 at S344 influences its stability, with a reduced turnover rate when FBXO28 is phosphorylated (Fig 2). Thus, CDKs not only control SCF^FBXO28^ activity, but also impact on FBXO28 protein levels and stability (Fig 2), presumably through a mechanism involving auto-ubiquitylation and self-destruction of FBXO28 at low S-phase CDK activity (Supporting Information Fig S2G and H). Exactly how phosphorylation at S344 affects SCF activity towards MYC remains to be determined, but S344 phosphorylation does not appear to be essential for FBXO28 to interact with MYC (Supporting Information Fig S4G). It is noteworthy that the S344 is positioned in a loop between two predicted helices in the FBXO28 C-terminus, potentially influencing FBXO28 dimerization and/or SCF activity through a conformational change triggered by CDK-dependent phosphorylation. However, we cannot exclude that binding between FBXO28 and MYC is regulated by CDK-mediated phosphorylation at other sites or by other modifications, since somewhat reduced binding of the stable ΔF-FBXO28 mutant to MYC was observed in G1 and early S phase (Supporting Information Fig S4F).

Intriguingly, CDK1 and CDK2 also target MYC for phosphorylation (Hydbring et al, 2010; Sjostrom et al, 2005), thus regulating the transcriptional activity of MYC (Hydbring et al, 2010). Thus, distinct CDK activities may operate and coordinate FBXO28 and MYC function during the cell cycle, and the discovery of FBXO28 therefore introduces a mechanistic link between activation of CDKs and downstream activation of the MYC pathway. In fact, many of the features of FBXO28, such as effects on proliferation, and its connection to tumourigenesis, might be directly linked to the MYC pathway. Our observation that knockdown of FBXO28 in MYC-depleted cells does not result in any further reduction in proliferation supports this notion. The findings that FBXO28 depletion or expression of dominant-negative FBXO28 (ΔF-FBXO28) impairs MYC-induced oncogenic transformation and tumour growth indicate that FBXO28 is a rate-limiting factor in MYC-driven tumourigenesis, although we cannot rule out the possibility that FBXO28 ubiquitylates additional substrates and has MYC- and/or CDK-independent functions as well.

One of the major discoveries in this study is the result that high expression and phosphorylation of FBXO28 positively correlate with several adverse clinicopathological characteristics and poor outcome in breast cancer (7 and 8). Importantly, we found a strong association between poor overall-, breast-cancer specific- and relapse-free-survival and high FBXO28 protein levels in two independent cohorts, and in multivariate analysis, expression and phosphorylation of FBXO28 were independent predictors of poor survival (Supporting Information Tables S5 and S6). Since FBXO28 phosphorylation and overall expression levels are coupled to CDK activity, it is possible that the correlation between high FBXO28 levels and poor outcome partly reflects an increased proliferation in the tumours with high CDK activity. However, high FBXO28 levels were also found to be associated with poor prognosis in particular patient subgroups, including low proliferative luminal A tumours and in highly proliferative (high Ki-67 levels), poorly differentiated breast tumours (Fig 8E and Supporting Information Fig S7E), presumably tumours with hyperactivation of MYC (Xu et al, 2010).

Collectively, our data suggest that the E3 ubiquitin ligase SCF^FBXO28^ is a downstream effector of CDK1/2 that activates MYC's transcriptional function by non-proteolytic ubiquitylation. The findings in this study of a mechanistic link between MYC activity and FBXO28, and the positive association between high *FBXO28* expression and the expression of MYC target genes in human breast cancer suggest a potential role for FBXO28 in MYC-driven breast cancers and underscores the importance of FBXO28 as a new potential biomarker and candidate for future drug development, for instance via CDK inhibition, for the treatment of breast tumours with overexpression of FBXO28 and/or MYC.

## MATERIALS AND METHODS

### Cell Culture, Transfections and Treatments

Tumour-derived cell lines were cultured according to the guidelines at the American Type Culture Collection (ATCC) and Clontech. Tet-On U2OS cells for inducible FBXO28 expression were generated by stable transfection with a pTRE2-PUR-myc3-FBXO28 WT or ΔF-box constructs and selection with puromycin. FBXO28 expression was induced by addition of doxycycline for 24 h. Transient plasmid transfections were performed using TransIT-LT1 (MIRUS, Madison, WI) or Lipofectamine 2000 (Invitrogen, Carlsbad, CA) reagents, according to the manufacturer's protocols. siRNA transfections were carried out using HiPerFect transfection reagent, as recommended by the manufacturer's (Qiagen). For the generation of retroviruses, the Phoenix-Eco packaging cell line was transfected with the indicated pBabe-puro/zeo expression plasmids using the calcium phosphate method. Viral supernatants were harvested 48 h after transfection and used for transduction of *p53*^−/−^ MEFs. For colony assays, cells were fixed with 70% ethanol and stained with crystal violet. Treatment with doxycycline (5 µg/ml), puromycin (1 µg/ml), cycloheximide (50 mg/ml), MG-132 (5–25 µM), thymidine (2 mM) nocodazole (125 ng/ml), or roscovitine (10 µM) was carried out as indicated. Chemicals were purchased from Sigma.

### siRNA functional library screen and proliferation analysis

The proliferative potential of siRNA loss-of-function was examined using the cell spot microarray (CSMA) technology as previously described (Rantala et al, 2011). Cells were stained with anti-EdU Alexa 647 and Propidium Iodide dyes according to manufacturer's instructions (Invitrogen) and analysed on a FACS LSRII (BD Biosciences). FACS data analysis was carried out using the CELLQuest software (BD Biosciences).

### Microarray, qRT-PCR and reporter assays

HCT116 cells were transfected with siRNA oligonucleotides targeting *FBXO28* or a scrambled control sequence for the times indicated. RNA was extracted using TRIzol reagent (Invitrogen) according to manufacturer's instructions. Gene expression analysis was carried out as previously described (Klevebring et al, 2009) using Operon microarray slides (Operon Biotechnologies; 34,000 probes). The analysis was based on equal amount of cells and equal amount of RNA per cell. Manufacturer-provided microarray spike-in hybridization controls were used at a 1:1 ratio for RNA normalization. For quantitive real-time PCR (qRT-PCR), total RNA was transcribed into cDNA using random hexamer primers (Fermentas) and SuperScript II Reverse transcriptase (Invitrogen). qRT-PCR was performed in triplicates using SYBR Green Mix (Applied Biosystems) and analysed on an ABI 7300 Real Time PCR System with SDS software version 1.3.1. PCR primer sequences are available from authors upon request. TaqMan probes were used for quantification of FBXO28 mRNA (Applied Biosystems). mRNA expression was calculated according to the ΔΔ*C*_t_ quantification method and the relative expression level of individual genes was obtained by normalizing the expression to *b-ACTIN* mRNA expression. Error bars represent standard deviation of triplicates. Luciferase reporter assays were performed in HeLa cells transfected with the mintkM4-Luc promoter construct containing four E-boxes. The RSV-β-Galactosidase or Renilla vectors were used for normalization. Luciferase activities were quantified in a luminometer (VICTOR3, Perkin–Elmer, Massachusetts, USA) using the Dual-Luciferase Reporter Assay System according to the manufacturer's protocol (Promega). Fold change was averaged from at least two separate experiments performed in triplicates.

### Immunofluorescence microscopy and immunoprecipitation

Cells were grown on cover slips and fixed in 4% paraformaldehyde (PFA), permeabilized with 0.1% Triton X-100 and incubated with primary antibodies as indicated. Proteins were detected by staining using appropriate secondary antibodies. DNA was counterstained with Hoechst (Sigma) or DAPI. Slides were mounted using Vectashield (Vector Laboratories), and images were acquired on a Zeiss Axioplan II imaging microscope equipped with an AxioCam CCD camera (Carl Zeiss) with Axiovision software (Thornwood, NY).

Cells were lysed on plates in M-RIPA buffer (50 mM Tris-HCl pH 8, 250 mM NaCl, 1% Nonidet P-40, 0.5% deoxycholic acid, 0.05% SDS) supplemented with Complete protease inhibitor cocktail (Roche) and Halt Phosphatase inhibitor cocktail (Pierce, Rockford, IL), followed by brief sonication and centrifugation (10,000 × *g* for 15 min) to clarify the lysates. Precleared protein extracts (200–500 μg) were incubated using the indicated antibodies or control IgG antibody at 4°C overnight and protein complexes were subsequently collected by incubation with Gammabind G Sepharose beads (GE Healthcare) for 1 h at 4°C. The resin was washed five times with lysis buffer and associated proteins eluted by boiling in SDS sample buffer under reducing conditions, after which proteins were resolved on SDS–PAGE gels and transferred to PVDF membranes. Proteins were detected by Western blotting procedures under standard conditions using an enhanced chemiluminescence system (Perkin–Elmer).

**The paper explained**

PROBLEM:

Among the approximately 70 F-box genes, which encode substrate-recognition subunits of SCF ubiquitin ligases, the biological functions for only a handful have been unraveled so far. Given the critical function of SCF ubiquitin ligases in targeting regulatory and cancer-associated proteins for ubiquitylation, a major goal in ubiquitin research is to identify key oncogenic SCF ubiquitin ligases and their specific target substrates.

RESULTS:

We identified the F-box protein FBXO28 as part of a SCF complex that acts as a critical regulator of tumour cell proliferation and an important modifier of MYC function. SCF^FBXO28^ ubiquitylates MYC during the cell cycle after being phosphorylated/activated by cyclin-dependent kinases (CDKs), but does not destabilize MYC. Rather, SCF^FBXO28^ promotes MYC-driven transcription, proliferation and tumour development, by mediating recruitment of the cofactor p300 to MYC target genes. Finally, high FBXO28 phosphorylation and expression are very strong and independent predictors of poor outcome in unrelated cohorts of breast cancer.

IMPACT:

We identify FBXO28 as a new prognostic factor in breast cancer and rate-limiting factor in MYC-driven tumourigenesis. Our work shows that SCF^FBXO28^ is a downstream effector of CDK1/2 and suggests that FBXO28 acts as a link between CDKs and activation of MYC during the cell cycle. Thus, FBXO28 may be a novel biomarker of poor outcome in breast cancer and a new potential drug target in MYC-driven tumours.

### Patients and tumour analysis

Breast cancer specimens were obtained from resection of the breast or lumpectomy at the Department of Obstetrics and Gynecology, Innsbruck Medical University, Austria and from the Department of Pathology, Malmö University Hospital, Sweden. All samples were collected in compliance with and approved by the Institutional Review Board and with informed consent from the patients. Ethical approvals were obtained by the relevant Ethics Committees at the Innsbruck Medical University, Austria and by the Ethics Committee at Lund University, Sweden. All experiments involving human specimens conformed to the principles set out in the WMA Declaration of Helsinki and the NIH Belmont Report.

All animal studies were carried out in accordance with approved protocols from the Institutional Animal Care and Use Committee. For additional details on mouse and human tumours, tissue microarray (TMA) construction, immunohistochemistry (IHC) and image evaluation, see Supporting Information.

### Statistical analysis

Chi-square and Spearman's rho correlation tests were used for comparison of phosphorylated FBXO28 in the nucleus and relevant clinicopathological characteristics. Survival curves were plotted using the Kaplan–Meier analysis and the log-rank test was used to illustrate differences between OS, breast cancer specific survival (BCSS) and RFS according to the nuclear fraction (NF) of phosphorylated FBXO28. Cox proportional univariate and multivariate hazard models were used to estimate the impact of phosphorylated FBXO28 on OS, BSS and RFS adjusting for patient age, tumour size, ER status, nodal status, HER2 and Nottingham histological grade (NHG). Repeated analyses were performed on specific patient subgroups, including luminal A and high-grade tumours, separately. All calculations were performed using the SPSS version 19.0 (SPSS Inc, Chicago, USA) and statistical tests (Student's *t*-test and Fishers' exact test) with a *p*-value < 0.05 were considered statistically significant.

Additional methods are presented in the Supporting Information.

## Supplementary material

As a service to our authors and readers, this journal provides supporting information supplied by the authors. Such materials are peer reviewed and may be re-organized for online delivery, but are not copy-edited or typeset. Technical support issues arising from supporting information (other than missing files) should be addressed to the authors.

**Figure S1. (A)** Inhibition of cell proliferation upon FBXO28 depletion.

**Figure S2. (A)** FBXO28 protein is evolutionary conserved.

**Figure S3. (A)** Downregulation of MYC target genes upon depletion of FBXO28 by siRNA transfection in HCT116 cells.

**Figure S4. (A)** MCF-7 cell extracts cotransfected with the indicated expression constructs were immunoprecipitated with FLAG antibodies followed by immunoblotting with MYC (N262) antibody as indicated.

**Figure S5. (A)** Luciferase assay in HeLa cells transfected with the salt-induced kinase (SIK) gene luciferase reporter for 48 h, in combination with the indicated expression constructs or siRNAs.

**Figure S6. (A)** *P53*−/− MEF cells were embedded in soft-agar medium after co-transduction with shFBXO28 vectors or a shRNA-GFP control vector and MYC, as indicated.

**Figure S7. (A)** IB analysis was performed in a panel of primary breast tumor specimens (cohort 1).

**Table S1.** Top ranked F-box genes that reduced cell proliferation in KPL4 cells upon RNAi in the functional screen shown in Fig 1B.

**Table S2.** FBXO28 is overexpressed in primary breast cancer.

**Table S3.** FBXO28 expression in human breast cancer correlates with a gene set associated with MYC and p300 activity as determined by ChIP-seq.

**Table S4.** Excel spread sheet.

**Table S5A.** Correlation between clinicopathological characteristics of breast cancer patients with low (<25%) and high (>25%) nuclear fraction (NF) of phosphorylated FBXO28 (Cohort 2, *N* = 144).

**Table S5B.** Cox univariate analysis for overall survival (OS) and breast-cancer specific survival (BCSS) according to the nuclear fraction (NF) of phosphorylated FBXO28 and other established clinocpathological parameters.

**Table S5B.** Cox univariate analysis for overall survival (OS) and breast-cancer specific survival (BCSS) according to the nuclear fraction (NF) of phosphorylated FBXO28 and other established clinocpathological parameters.

**Table S5C.** Cox multivariate analysis for overall survival (OS) and breast cancer specific survival (BCSS) according to the nuclear fraction (NF) of phosphorylated FBXO28 (Cohort 2, *N* = 144).

**Table S6A.** Correlation between clinicopathological characteristics and FBXO28 protein phosphorylation and expression in the nucleus (Cohort 3, *N* = 498).

**Table S6B.** Cox multivariate analysis for overall survival (OS) and breast cancer specific survival (BCSS) according to FBXO28 phosphorylation and expression level (Cohort 3, *N* = 498).

**Table S6C.** Cox univariate analysis for overall survival (OS) and breast-cancer specific survival (BCSS) according to FBXO28 phosphorylation and expression (Cohort 3, *N* = 498).

## References

[b1] Adhikary S, Eilers M (2005). Transcriptional regulation and transformation by Myc proteins. Nat Rev Mol Cell Biol.

[b2] Adhikary S, Marinoni F, Hock A, Hulleman E, Popov N, Beier R, Bernard S, Quarto M, Capra M, Goettig S (2005). The ubiquitin ligase HectH9 regulates transcriptional activation by Myc and is essential for tumor cell proliferation. Cell.

[b3] Bai C, Sen P, Hofmann K, Ma L, Goebl M, Harper JW, Elledge SJ (1996). SKP1 connects cell cycle regulators to the ubiquitin proteolysis machinery through a novel motif, the F-box. Cell.

[b4] Bashir T, Pagano M (2003). Aberrant ubiquitin-mediated proteolysis of cell cycle regulatory proteins and oncogenesis. Adv Cancer Res.

[b5] Bouchard C, Marquardt J, Bras A, Medema RH, Eilers M (2004). Myc-induced proliferation and transformation require Akt-mediated phosphorylation of FoxO proteins. EMBO J.

[b6] Carrano AC, Eytan E, Hershko A, Pagano M (1999). SKP2 is required for ubiquitin-mediated degradation of the CDK inhibitor p27. Nat Cell Biol.

[b7] Cenciarelli C, Chiaur DS, Guardavaccaro D, Parks W, Vidal M, Pagano M (1999). Identification of a family of human F-box proteins. Curr Biol.

[b8] Chen ZJ, Sun LJ (2009). Nonproteolytic functions of ubiquitin in cell signaling. Mol Cell.

[b9] Choi SH, Wright JB, Gerber SA, Cole MD (2010). Myc protein is stabilized by suppression of a novel E3 ligase complex in cancer cells. Genes Dev.

[b10] Cope GA, Deshaies RJ (2006). Targeted silencing of Jab1/Csn5 in human cells downregulates SCF activity through reduction of F-box protein levels. BMC Biochem.

[b11] Dennis G, Sherman BT, Hosack DA, Yang J, Gao W, Lane HC, Lempicki RA (2003). DAVID: database for annotation, visualization, and integrated discovery. Genome Biol.

[b12] Eberhardy SR, Farnham PJ (2001). c-Myc mediates activation of the cad promoter via a post-RNA polymerase II recruitment mechanism. J Biol Chem.

[b13] Ecker A, Simma O, Hoelbl A, Kenner L, Beug H, Moriggl R, Sexl V (2009). The dark and the bright side of Stat3: proto-oncogene and tumor-suppressor. Front Biosci.

[b14] Eilers M, Eisenman RN (2008). Myc's broad reach. Genes Dev.

[b15] Faiola F, Liu X, Lo S, Pan S, Zhang K, Lymar E, Farina A, Martinez E (2005). Dual regulation of c-Myc by p300 via acetylation-dependent control of Myc protein turnover and coactivation of Myc-induced transcription. Mol Cell Biol.

[b16] Fredlund E, Ringner M, Maris JM, Pahlman S (2008). High Myc pathway activity and low stage of neuronal differentiation associate with poor outcome in neuroblastoma. Proc Natl Acad Sci USA.

[b17] Frescas D, Pagano M (2008). Deregulated proteolysis by the F-box proteins SKP2 and beta-TrCP: tipping the scales of cancer. Nat Rev Cancer.

[b18] Guccione E, Martinato F, Finocchiaro G, Luzi L, Tizzoni L, Dall'Olio V, Zardo G, Nervi C, Bernard L, Amati B (2006). Myc-binding-site recognition in the human genome is determined by chromatin context. Nat Cell Biol.

[b19] Hart M, Concordet JP, Lassot I, Albert I, del los Santos R, Durand H, Perret C, Rubinfeld B, Margottin F, Benarous R (1999). The F-box protein beta-TrCP associates with phosphorylated beta-catenin and regulates its activity in the cell. Curr Biol.

[b20] Hershko A, Ciechanover A (1998). The ubiquitin system. Annu Rev Biochem.

[b21] Hydbring P, Bahram F, Su Y, Tronnersjo S, Hogstrand K, von der Lehr N, Sharifi HR, Lilischkis R, Hein N, Wu S (2010). Phosphorylation by Cdk2 is required for Myc to repress Ras-induced senescence in cotransformation. Proc Natl Acad Sci USA.

[b22] Kim SY, Herbst A, Tworkowski KA, Salghetti SE, Tansey WP (2003). Skp2 regulates Myc protein stability and activity. Mol Cell.

[b23] Klevebring D, Gry M, Lindberg J, Eidefors A, Lundeberg J (2009). Automation of cDNA synthesis and labelling improves reproducibility. J Biomed Biotechnol.

[b24] Larsson LG, Henriksson MA (2010). The Yin and Yang functions of the Myc oncoprotein in cancer development and as targets for therapy. Exp Cell Res.

[b25] Lin CY, Loven J, Rahl PB, Paranal RM, Burge CB, Bradner JE, Lee TI, Young RA (2012). Transcriptional amplification in tumor cells with elevated c-Myc. Cell.

[b26] Loi S, Haibe-Kains B, Desmedt C, Lallemand F, Tutt AM, Gillet C, Ellis P, Harris A, Bergh J, Foekens JA (2007). Definition of clinically distinct molecular subtypes in estrogen receptor-positive breast carcinomas through genomic grade. J Clin Oncol.

[b27] Meyer N, Penn LZ (2008). Reflecting on 25 years with MYC. Nat Rev Cancer.

[b28] Nakayama KI, Nakayama K (2006). Ubiquitin ligases: cell-cycle control and cancer. Nat Rev Cancer.

[b29] Nie ZQ, Hu GQ, Wei G, Cui KR, Yamane A, Resch W, Wang RN, Green DR, Tessarollo L, Casellas R (2012). c-Myc is a universal amplifier of expressed genes in lymphocytes and embryonic stem cells. Cell.

[b30] Pickart CM (2004). Back to the future with ubiquitin. Cell.

[b31] Popov N, Schulein C, Jaenicke LA, Eilers M (2010). Ubiquitylation of the amino terminus of Myc by SCF(beta-TrCP) antagonizes SCF(Fbw7)-mediated turnover. Nat Cell Biol.

[b32] Rahal R, Amon A (2008). Mitotic CDKs control the metaphase-anaphase transition and trigger spindle elongation. Genes Dev.

[b33] Rahl PB, Lin CY, Seila AC, Flynn RA, McCuine S, Burge CB, Sharp PA, Young RA (2010). c-Myc regulates transcriptional pause release. Cell.

[b34] Rantala JK, Makela R, Aaltola AR, Laasola P, Mpindi JP, Nees M, Saviranta P, Kallioniemi O (2011). A cell spot microarray method for production of high density siRNA transfection microarrays. BMC Genomics.

[b35] Rhodes DR, Yu J, Shanker K, Deshpande N, Varambally R, Ghosh D, Barrette T, Pandey A, Chinnaiyan AM (2004). ONCOMINE: a cancer microarray database and integrated data-mining platform. Neoplasia.

[b36] Rodier G, Coulombe P, Tanguay PL, Boutonnet C, Meloche S (2008). Phosphorylation of Skp2 regulated by CDK2 and Cdc14B protects it from degradation by APC(Cdh1) in G1 phase. EMBO J.

[b37] Sjostrom SK, Finn G, Hahn WC, Rowitch DH, Kenney AM (2005). The Cdk1 complex plays a prime role in regulating N-myc phosphorylation and turnover in neural precursors. Dev Cell.

[b38] Skaar JR, D'Angiolella V, Pagan JK, Pagano M (2009). SnapShot: F box proteins II. Cell.

[b39] Skowyra D, Craig KL, Tyers M, Elledge SJ, Harper JW (1997). F-box proteins are receptors that recruit phosphorylated substrates to the SCF ubiquitin-ligase complex. Cell.

[b40] Soderberg O, Gullberg M, Jarvius M, Ridderstrale K, Leuchowius KJ, Jarvius J, Wester K, Hydbring P, Bahram F, Larsson LG (2006). Direct observation of individual endogenous protein complexes in situ by proximity ligation. Nat Methods.

[b41] Strohmaier H, Spruck CH, Kaiser P, Won KA, Sangfelt O, Reed SI (2001). Human F-box protein hCdc4 targets cyclin E for proteolysis and is mutated in a breast cancer cell line. Nature.

[b42] Subramanian A, Tamayo P, Mootha VK, Mukherjee S, Ebert BL, Gillette MA, Paulovich A, Pomeroy SL, Golub TR, Lander ES (2005). Gene set enrichment analysis: a knowledge-based approach for interpreting genome-wide expression profiles. Proc Natl Acad Sci USA.

[b43] Thomas LR, Tansey WP (2011). Proteolytic control of the oncoprotein transcription factor Myc. Adv Cancer Res.

[b44] Vervoorts J, Luscher-Firzlaff JM, Rottmann S, Lilischkis R, Walsemann G, Dohmann K, Austen M, Luscher B (2003). Stimulation of c-MYC transcriptional activity and acetylation by recruitment of the cofactor CBP. EMBO Rep.

[b45] von der Lehr N, Johansson S, Wu S, Bahram F, Castell A, Cetinkaya C, Hydbring P, Weidung I, Nakayama K, Nakayama KI (2003). The F-box protein Skp2 participates in c-Myc proteosomal degradation and acts as a cofactor for c-Myc-regulated transcription. Mol Cell.

[b46] Weissman AM (2001). Themes and variations on ubiquitylation. Nat Rev Mol Cell Biol.

[b47] Welcker M, Orian A, Jin J, Grim JE, Harper JW, Eisenman RN, Clurman BE (2004). The Fbw7 tumor suppressor regulates glycogen synthase kinase 3 phosphorylation-dependent c-Myc protein degradation. Proc Natl Acad Sci USA.

[b48] Whitfield ML, Sherlock G, Saldanha AJ, Murray JI, Ball CA, Alexander KE, Matese JC, Perou CM, Hurt MM, Brown PO (2002). Identification of genes periodically expressed in the human cell cycle and their expression in tumors. Mol Biol Cell.

[b49] Xu J, Chen Y, Olopade OI (2010). MYC and Breast Cancer. Genes Cancer.

[b50] Yada M, Hatakeyama S, Kamura T, Nishiyama M, Tsunematsu R, Imaki H, Ishida N, Okumura F, Nakayama K, Nakayama KI (2004). Phosphorylation-dependent degradation of c-Myc is mediated by the F-box protein Fbw7. EMBO J.

[b51] Zhao X, Heng JI, Guardavaccaro D, Jiang R, Pagano M, Guillemot F, Iavarone A, Lasorella A (2008). The HECT-domain ubiquitin ligase Huwe1 controls neural differentiation and proliferation by destabilizing the N-Myc oncoprotein. Nat Cell Biol.

