# Supplementary material for: CDK-mediated activation of the SCFFBXO28 ubiquitin ligase promotes MYC-driven transcription and tumourigenesis and predicts poor survival in breast cancer
Source: EMBO Mol Med. 2013 Jun 14;5(7):999–1018. doi: 10.1002/emmm.201202341 (PMC3721474; doi:10.1002/emmm.201202341)
Supplement: Supplementary file 1 [file emmm0005-0999-SD1.pdf]

EMBO Molecular Medicine

CDK-mediated activation of the SCF^{FBXO28} ubiquitin ligase promotes MYC-driven transcription and tumorigenesis and predicts poor survival in breast cancer

Diana Cepeda, Hwee-Fang Ng, Hamid Reza Sharifi, Salah Mahmoudi, Vanessa Soto Cerrato, Erik Fredlund, Kristina Magnusson, Helén Nilsson, Alena Malyukova, Juha Rantala, Daniel Klevebring, Francesc Viñals, Nimesh Bhaskaran, Siti Mariam Zakaria, Suryo Rahmanto, Stefan Grotegut, Michael Lund Nielsen, Cristina Al-Khalili Szigyarto, Dahui Sun, Mikael Lerner, Sanjay Navani, Martin Widschwendter, Mathias Uhlén, Karin Jirström, Fredrik Pontén, James Wohlschlegel, Dan Grandér, Charles Spruck, Lars-Gunnar Larsson, and Olle Sangfelt

Corresponding authors: Olle Sangfelt and Lars-Gunnar Larsson, Karolinska Institutet

Review timeline:

Submission date:	06 December 2012
Editorial Decision:	29 December 2012
Revision received:	12 April 2013
Editorial Decision:	29 April 2013
Revision received:	09 May 2013
Accepted:	10 May 2013

Transaction Report:

(Note: With the exception of the correction of typographical or spelling errors that could be a source of ambiguity, letters and reports are not edited. The original formatting of letters and referee reports may not be reflected in this compilation.)

Editor: Roberto Buccione

1st Editorial Decision 29 December 2012

Thank you for the submission of your manuscript to EMBO Molecular Medicine.

In this case we experienced unusual difficulties in securing three appropriate reviewers in a timely manner. Since we have received consistent evaluations from 2 Reviewers and cannot justify a further delay, I have decided to proceed based on these evaluations.

You will see that while both Reviewers are generally supportive of your work and underline its considerable potential interest, they also both raise a number of specific concerns that prevent us from considering publication at this time.

I agree with Reviewer 1's concern that to distinguish specific from more general effects on Myc activity is an interpretation issue that needs to be solved. S/he suggests avenues and appropriate critical controls to address this. Reviewer 1 also notes that the Myc/FBXO28 association experiment illustrated in Fig 4A is potentially flawed.

Reviewer 2 points to two general issues. On one hand, s/he notes that any agent affecting cell proliferation is bound to affect colony formation; hence the specificity of the effects of FBX028 in

this respect needs to be established. The other is his/her impression that the correlation between high nuclear FBX028 and survival is indirect and not causal, and should be accordingly discussed. Reviewer 2 also indicates other items that need to be acted upon experimentally, including a more rigorous analysis of the role of FBX028 phosphorylation and an inconsistency regarding the effects of deltaF-FXB028 on Myc stability.

While publication of the paper cannot be considered at this stage, we would be pleased to consider a suitably revised submission in the future, provided, however, that the Reviewers' concerns are fully addressed with additional experimental data where appropriate.

Please note that it is EMBO Molecular Medicine policy to allow a single round of revision only and that, therefore, acceptance or rejection of the manuscript will depend on the completeness of your responses included in the next, final version of the manuscript.

As you know, EMBO Molecular Medicine has a "scooping protection" policy, whereby similar findings that are published by others during review or revision are not a criterion for rejection. However, I do ask you to get in touch with us after three months if you have not completed your revision, to update us on the status. Please also contact us as soon as possible if similar work is published elsewhere.

I look forward to seeing a revised form of your manuscript as soon as possible.

***** Reviewer's comments *****

Referee #1 (General Remarks):

This report adds an interesting new layer to the many known levels of Myc regulation. The authors report an siRNA screen that uncovered the F-box protein FBXO28 as being important in tumor cell proliferation. They go on to show that this ubiquitin ligase is phosphorylated by CDK1/2 and that phospho-FBXO28 is activated to carry out ubiquitylation of Myc which in turn promotes Myc interaction with the P300 histone acetyltransferase. The authors demonstrate, using a dominant-negative (F) as well as siFBXO28 that FBXO28 is required for Myc transcriptional activity, that Myc and FBXO28 are associated with a subset of Myc target genes, and that P300 binding and H4 acetylation at these sites is dependent on the presence of FBXO28. They also show that Myc-driven tumorigenesis requires FBXO28 and that levels of this ligase are predictive of outcome in breast cancers. The authors conclude that FBXO28 defines a pathway that links CDK activity to Myc's molecular and physiological functions.

Overall this is an interesting and well executed study that reveals another player in the regulation of Myc activity. However one problem in interpretation is distinguishing specific and direct effects on Myc activity from more general effects on cellular processes. To some extent this problem can be addressed with additional controls as suggested below.

1. Fig. 1- The effects of FBXO28 depletion are clearly shown to impair tumor cell growth, however its effects on normal, non-transformed cell growth are not described. Does inhibition or loss of FBXO28 block cell cycle progression of normal fibroblasts? This would have some relevance for the use of inhibitors of FBXO28 as a cancer therapeutic. The authors could consider examining the sensitivity to FBXO28 depletion in myc-null rat fibroblasts with and without Myc.

2. Fig. 3 - As the authors are likely aware, there has been recent discussion in the literature about whether specific Myc target genes actually exist and whether the expression array approaches used in many studies of Myc accurately reflect Myc-induced changes in gene expression. This is not mentioned in the present paper, however because the concept of a Myc signature which is affected by FBXO28 is important in this work, the authors should indicate how they have normalized their RNA samples and whether they applied the "spiking" procedure for attaining normalization. They should also discuss how the recent studies impact on their interpretation of how FBXO28 affects Myc transcription.

3. Fig. 4A - In the experiment showing specific association of Myc with FBXO28 it is somewhat disturbing that the the level of input FBXO28 is probably five fold greater than the other FBXOs

being tested. If all the FBXOs were input at the same level would the specificity of the interaction be the same? In addition the FBXOs tested should definitely include those that were among the top hits in the tumor inhibition screen as shown in Table S1. Is it possible that all these ligases regulate Myc?

4. Fig. 4D - the isPLA assay is not particularly well described. I may have missed it but I don't think there was a literature citation either.

5. Fig. 4E - In this experiment the WT FBXO28 ubiquitination is quite weak (compare to FBXW7) and not strikingly different from the EV control. Fig 4F is much more convincing. Better data is needed for 4E.

6. Fig, 4G - the FBXO28 S344A mutant is "largely deficient in catalyzing Myc ubiquitylation", but not completely dead. There is some concern here that the amounts of Cul1 and possibly FBXO28 itself are lower for the mutant than for the WT and phospho-mimetic forms. This raises the possibility that there may be other sites in Myc whose FBXO28-mediated ubiquitylation are not dependent on S344 phosphorylation. This should be mentioned.

7. Fig. 5A - the effects of the F-FBXO28 and FBXO28 KD on Myc reporter gene expression are consistent with a requirement for FBXO28 modification of Myc for its transcriptional activity. However it would also would be consistent with a role for FBXO28 more generally for transcription. The authors should include a control reporter driven by another transcription factor (e.g. E2F) to rule out (or rule in) a general effect. Also, does overexpression of WT FBXO28 increase the level of reporter gene expression?

8. Fig 5C - the authors conclude that FBXO28 is required for efficient Myc-driven transcription during S phase. How drastically is S phase progression and onset of G2/M affected by FBXO28 KD or dominant negative form? Fig 1C indicates that there is an effect. If so then it will be difficult to assess whether there is a specific effect on Myc or on S phase transcription in general upon FBXO28 inhibition. One approach would be to compare global gene expression during S phase in FBXO28 KD and control cells.

9. Fig 5E, F, G- the extent of P300 binding and H4 acetylation should be determined at promoters that are not bound by Myc. This control would help determine how general the effects of FBXO28 depletion are. In addition, the authors should determine whether recruitment of GCN5, the other critical acetyltransferase bound by Myc, is also affected.

10. Fig. 5- For this and other figures the authors should provide p-values and indicate the number of biological replicates used.

11. Fig S5H - it appears that the FBXO28 inhibition results in increased ubiquitylation of the Myc K6R mutant. This needs to be explained.

Referee #2 (Comments on Novelty/Model System):

The manuscript by Cepeda and colleagues makes significant contributions to our understanding of ubiquitin ligases, the complex regulation of MYC, and suggests a role for FBXO28 in tumorigenesis. This is an extensive study of FBXO28 function and adds an important new layer to the regulation of MYC, which should be of great interest to the readers of EMBO Molecular Medicine.

Referee #2 (General Remarks):

Cepeda and colleagues define a potential function for the previously uncharacterized F-box protein FBXO28. The authors demonstrate that FBXO28 forms a functional SCF complex that ubiquitylates MYC, and suggest that SCF-FBXO28 activity is regulated by CDK-mediated phosphorylation in a cell cycle dependent manner. Cepeda and colleagues provide a series of data that collectively support a role of FBXO28 in proteolysis-independent, but ubiquitylation-dependent activation of

MYC. Overall the authors suggest that Cdk-dependent phosphorylation of FBXO28 promotes ubiquitylation of MYC, activating MYC-dependent transcription, transformation, and tumorigenesis. Additionally, data collected from multiple cohort studies and from online databases indicate a correlation between high levels of phosphorylated FBXO28 and poor patient outcome. The functional characterization of FBXO28 and its role in MYC activation is convincing and supported by mostly high quality data. The role of FBXO28 in transformation and tumorigenesis is suggestive, but I believe the authors should be more cautious in interpretation of these results and need to consider alternative explanations, because: (1) It is not entirely unexpected that expression of deltaFbox-FBXO28 blocks colony formation (Fig. 8B) and reduces tumor volume (8D), since the authors have demonstrated extensively, that expression of deltaFbox-FBXO28 (or knock down of FBXO28) blocks cell proliferation of tumor cells. Any agent that blocks cell proliferation can be expected to have a similar effect. Thus, the specificity of these effects is unclear. (2) The correlation between high nuclear fraction of pS344-FBXO28 and overall survival could be an indirect correlation, because pS344-FBXO28 is largely restricted to cells in S and G2/M phase (figure 2B) and a high nuclear fraction of pS344-FBXO28 may thus simply distinguish highly proliferative tumors from less proliferative ones. Although this doesn't diminish the utility of pS344-FBXO28 as a marker, by reading the manuscript I got the impression that the authors interpret these results as a causative relation. Careful rewriting of these sections and discussing these alternative interpretations could improve the manuscript.

Despite these concerns, I recommend publication in EMBO Molecular Medicine if the authors address the above in addition to the specific concerns listed below. The manuscript by Cepeda and colleagues makes significant contributions to our understanding of ubiquitin ligases, the complex regulation of MYC, and suggests a role for FBXO28 in tumorigenesis. This is an extensive study of FBXO28 function and adds an important new layer to the regulation of MYC, which should be of great interest to the readers of EMBO Molecular Medicine.

Data analysis in figures 7 and 8: Please note that analyses shown in figures 7 and 8 are outside my field of expertise, and I cannot evaluate the validity of statistical methods used to generate these data.

Major Concerns:

(1) The role of FBXO28 phosphorylation in its regulation should be addressed more rigorously, because it is mainly based on mutants of residue 344. The authors should immunopurify FBXO28 from synchronized cells in G1 (no phosphorylation) and S-phase or G2/M (high phosphorylation) and show MYC ubiquitylation in vitro similar to the experiment in Fig. 4G. If phosphorylation stimulates MYC ubiquitylation as suggested by the authors based on results with mutations, then the S or G2/M sample should be significantly more active.

(2) Related to point 1, I was somewhat surprised that the authors did not attempt to address whether FBXO28 phosphorylation affects recognition/binding of MYC. They show that SCF-FBXO28 formation is unaffected by phosphorylation, but to not attempt to demonstrate any effect on MYC binding. Cell lysates from synchronized cells as mentioned in point 1 could be used to address this question.

(3) Figure 5G: The authors demonstrate convincingly in figures 4H to J that FBXO28 does not affect MYC stability. However, in figure 5G (input panel) there seems to be a pretty significant increase in the steady state levels of MYC when F-FBOXO28 is expressed. This internal inconsistency needs to be addressed somehow.

Minor points:
1) Abstract and Introduction: "Here we identify a novel F-box....". "Novel" may not be correct as FBOXO28 has been identified previously, although not characterized.

2) Introduction: The introduction should mention the two recent landmark studies describing MYC as an amplifier of transcription programs (Cell, Volume 151, 2012: Nie et al. as well as Lin et al.)

3) page 9, last line: Reference for (Strohmaier et al. 2011) missing

© EMBO

4) page 10 top: (Fig. 2G)? I cannot find figure 2G

5) page 10, bottom: MYC turnover in the absence of FBXW7 (unpublished observations). This seems to be an important piece of data that may be worth showing in the supplementary figure section.

6) Figure 4A: based on this figure the authors cannot conclude that FBXO43, FBXO5, FBXO16 do not interact with MYC. The expression levels for these F-box proteins are very low compared to FBXO28 and one would not expect to detect a signal at the exposure level shown even if they interacted with MYC. The authors should either mention that in the text, show a longer exposure, or remove these 3 F-box proteins from this figure.

7) Figure 4G should explain S/E and S/A in the figure legend

8) page 17: OS has not been defined.

9) page 24, line 4: comma before cycloheximide

10) page 25, line 1: performed

11) The authors need to be consistent with their labeling of figures. They mostly use F-FBOXO28, but for example figures 5C and E use FBOXO28-dF or dF-FBOXO28.

12) Figure 7B: The figure legend should give information about what level of overexpression is considered high, etc. What was the criterion?

13) Figure 7C: The table could be moved to the supplementary information. It is not very easy to understand, and the data results are nicely summarized in the text already.

14) Figure 8 legend: panel C states "Analysis was performed as in C." Should probably read ...as in B?

1st Revision - authors' response 12 April 2013

Reviewer #1

We are glad that the reviewer finds this an interesting and well-executed study that reveals another player in the regulation of MYC activity. We fully agree with that reviewer that is important to try to distinguishing specific and direct effects of FBXO28 on MYC activity from more general effects on cellular processes. It should, however, be pointed out that due to the global effects of MYC on gene expression and on multiple cellular processes this task is not trivial. We are thankful for the reviewer's different suggestions that we have addressed to the best of our ability as specified point by point below.

1. *Fig. 1- The effects of FBXO28 depletion are clearly shown to impair tumour cell growth, however its effects on normal, non-transformed cell growth are not described. Does inhibition or loss of FBXO28 block cell cycle progression of normal fibroblasts? This would have some relevance for the use of inhibitors of FBXO28 as a cancer therapeutic. The authors could consider examining the sensitivity to FBXO28 depletion in myc-null rat fibroblasts with and without Myc.*

We have now examined the effects of FBXO28 depletion in two different human normal fibroblast cell cultures, and find that DNA replication is reduced also in normal cells, although not to the same extent as in the tumour cell lines. This is expected when MYC function is impaired. The results are presented in a new Fig. 1C.

To address the MYC-FBXO28 interdependence further we have now also knocked down FBXO28 and MYC separately or together in HCT116 cells. As shown in the new Fig. 3E, MYC knockdown reduced EdU incorporation somewhat stronger than FBXO28 knockdown in these cells, but depletion of FBXO28 together with MYC knockdown did not lead to any further reduction in EdU, suggesting that MYC and FBXO28 are connected to the same pathway. As suggested by the reviewer we also silenced FBXO28 in wt and MYC-null rat fibroblasts using dox-inducible lentiviral shRNA. Given the time constraints for the re-submission, effects on proliferation were assessed in pools of transduced cells by measuring EdU incorporation. Unfortunately the knockdown was rather poor in the population as a whole. Nevertheless, by single cell analysis, no significant alteration following silencing of FBXO28 was observed in MYC-null cells, whereas reduced FBXO28 expression in the wt rat cells did result in lowered EdU incorporation (new Fig. S3C), consistent with the FBXO28 depletion in HCT116 cells. This suggests that the effects of FBXO28 on proliferation are indeed connected to MYC, although we can not exclude that FBXO28 has additional substrates that may contribute to the effect. This is now discussed in the results and in the discussion (pages 8 and 24) in the new version of the manuscript.

2. *Fig. 3 - As the authors are likely aware, there has been recent discussion in the literature about whether specific Myc target genes actually exist and whether the expression array approaches used in many studies of Myc accurately reflect Myc-induced changes in gene expression. This is not mentioned in the present paper, however because the concept of a Myc signature which is affected by FBXO28 is important in this work, the authors should indicate how they have normalized their RNA samples and whether they applied the "spiking" procedure for attaining normalization. They should also discuss how the recent studies impact on their interpretation of how FBXO28 affects Myc transcription.*

We thank the reviewer for pointing out this issue, and we apologize that we had not referred to this important discussion. In the new version we have cited the papers from Lin *et al* and Nie *et al* and other relevant publications in the introduction section. We find it convincing that MYC enhances global expression from E-box-containing promoters in an active or poised state, at least in the model systems that were under study. However, it is also clear that MYC still has different effects on different genes, for instance due to differences in the number of E-boxes per locus, or as a result of invasion of lower affinity binding sites particularly in enhancers at high enough MYC levels, collaboration with other transcription factors or cofactors, etc., which are relevant for the present study of MYC-FBXO28 cooperativity. For this reason previously reported “MYC signatures of gene expression” are still relevant, although differences between cell types will be observed and global effects of MYC should be taken into account.

In the present study the microarray analysis was performed at time points when there are minimal differences in cell number between the samples (see Fig. S1A) and we did not observe any significant differences in RNA levels at these time points. Therefore, manufacturer-provided microarray spike-in hybridization controls were used at a 1:1 ratio. This is pointed out in the new version of Materials & Methods.

3. *Fig. 4A - In the experiment showing specific association of Myc with FBXO28 it is somewhat disturbing that the level of input FBXO28 is probably five fold greater than the other FBXOs being tested. If all the FBXOs were input at the same level would the specificity of the interaction be the same? In addition the FBXOs tested should definitely include those that were among the top hits in the tumour inhibition screen as shown in Table S1. Is it possible that all these ligases regulate Myc?*

Unfortunately, it is difficult to obtain equal loading of the different F-box proteins, likely due to their different turnover rates in cells. In the new figure Fig. 4A, we have made equal exposures of the coimmunoprecipitation and the input blots. We have also included SKP2/FBXL1 (as a positive control) and FBXO22, one of the other F-box proteins whose depletion result in a significant loss of proliferation, as requested (see Fig. 1A). These data show that there are no signs of bands of

FBXO22 or other F-boxes in the coIPs that are visible in the input, in stark contrast to FBXO28 and SKP2. In addition, we have made reciprocal coIPs with FBXO22, FBXO28 and SKP2, showing that MYC only interacts with the latter two (Fig. S4A). However, we cannot exclude that MYC does interact with additional F-box proteins not yet analysed.

4. *Fig. 4D - the isPLA assay is not particularly well described. I may have missed it but I don't think there was a literature citation either.*

Sorry for this. We have now added in the reference "Söderberg et al" in the reference list and expanded the description of isPLA in Supporting Materials & Methods.

5. *Fig. 4E - In this experiment the WT FBXO28 ubiquitination is quite weak (compare to FBXW7) and not strikingly different from the EV control. Fig 4F is much more convincing. Better data is needed for 4E.*

Figure 4F shows that siRNA-mediated silencing of endogenous FBXO28 attenuates ubiquitylation of endogenous MYC, whereas Figure 4E demonstrates that ectopic expression of WT-FBXO28 further promotes MYC ubiquitylation (and ΔF-FBXO28 prevents MYC ubiquitylation). Despite that most tumour cell lines included in this paper have high endogenous FBXO28 protein levels (not shown), forced expression of WT-FBXO28 consistently increases MYC ubiquitylation further, although not to the same extent as FBXW7 which is the major ubiquitin ligase targeting MYC for proteasomal degradation. Overexpression of F-box proteins is complicated by the fact that critical components/post-translational modifications required for SCF activity might be limiting. Presumably, one explanation for the modest effects of WT-FBXO28 on MYC ubiquitylation might thus be lack of sufficient CDK activity and/or other cofactors required for SCF^{FBXO28} activity. Consistent with this possibility, we now provide in vivo MYC ubiquitylation data demonstrating that ectopic expression of the phosphorylation-mimicking mutant, SE-FBXO28, indeed promotes MYC ubiquitylation to a larger extent than WT-FBXO28 (new Fig 4H).

6. *Fig, 4G - the FBXO28 S344A mutant is "largely deficient in catalysing Myc ubiquitylation", but not completely dead. There is some concern here that the amounts of Cul1 and possibly FBXO28 itself are lower for the mutant than for the WT and phospho-mimetic forms. This raises the possibility that there may be other sites in Myc whose FBXO28-mediated ubiquitylation are not dependent on S344 phosphorylation. This should be mentioned.*

We acknowledge the thorough review of this result and the remark by rev#1 regarding different levels of the different FBXO28 mutants. In fact, it is correct that there are lower levels of the phosphorylation-deficient S344A-FBXO28 (compared to phospho-mimicking S344E mutant). It should be noted (now clarified in Supporting Materials and Methods) that we had to use three times more cells to pull-down similar amounts of S344A-FBXO28 as compared to S344E-FBXO28. During the submission process, we have performed additional experiments to investigate this further, and now provide data demonstrating that the S344A-mutant is intrinsically more unstable compared to the S344E-phospho-mimicking mutant (see also below, reviewer #2, point 1). Nevertheless, the data in Figure 4G demonstrate that i) S344 phosphorylation is not a requirement for SCF assembly, and that ii) the phosphorylation-deficient S344A-mutant is much less efficient in stimulating MYC ubiquitylation in vitro. However, we agree with the reviewer that its possible that the $SCF^{FBXO28\text{-}S344A}$ complex, although severely deficient in executing MYC ubiquitylation, is not completely 'dead', and at this stage we can not rule out that FBXO28 also promotes ubiquitylation of additional sites on MYC (in a S344-independent manner) (see below, reviewer #2, point 1). This is now mentioned in the paper (page 11).

7. *Fig. 5A - the effects of the ΔF-FBXO28 and FBXO28 KD on Myc reporter gene expression are consistent with a requirement for FBXO28 modification of Myc for its transcriptional*

activity. However it would also would be consistent with a role for FBXO28 more generally for transcription. The authors should include a control reporter driven by another transcription factor (e.g. E2F) to rule out (or rule in) a general effect. Also, does overexpression of WT FBXO28 increase the level of reporter gene expression?

This is a very relevant point. We have now included two additional luciferase reporter constructs, the salt-induced kinase (SIK) promoter and the Smad-responsive CAGA-promoter, the latter after induction by TGFβ. Both lack MYC-responsive E-boxes and we see no significant differences in activity of these reporters by overexpressing MYC or by depleting FBXO28. We have also expanded our analysis of mRNA expression following depletion of FBXO28, including two genes that do not contain MYC-responsive E-boxes. The expression of these genes is not significantly altered by depletion of FBXO28. These sets of data are now part of new Fig. 3C and D and Fig. S5A and B, and suggest that FBXO28 does not have major general effects on transcription, further supporting the specific link between MYC and FBXO28.

We have not observed any significant elevation of MYC-reporter activity upon overexpression of WT-FBXO28 (data not shown), possibly due to saturation or limitations in other components of the system. However, we found that induced expression of WT-FBXO28 in S-phase leads to enhanced expression of endogenous MYC target genes (now presented as new Fig. 5C).

8. *Fig 5C - the authors conclude that FBXO28 is required for efficient Myc-driven transcription during S phase. How drastically is S phase progression and onset of G2/M affected by FBXO28 KD or dominant negative form? Fig 1C indicates that there is an effect. If so then it will be difficult to assess whether there is a specific effect on Myc or on S phase transcription in general upon FBXO28 inhibition. One approach would be to compare global gene expression during S phase in FBXO28 KD and control cells.*

It is correct that KD and ΔF-expression attenuate cell cycle progression, including progression through S-phase (Fig. 1). However, it should be noted that FBXO28 KD or ΔF-FBXO28 expression do not lead to an acute cell cycle arrest and the effect on proliferation is clearly secondary (observed at 48-72 hrs, see Fig. S1A) compared to the effect on gene expression (observed already at 16-36 hrs, see Fig. 3B and S3A). As discussed above in response to the previous question, FBXO28 does not affect transcription in general but is "biased" towards MYC-regulated transcription as is also evident from Fig 3B.

The question whether the effect on S-phase (and G2/M) is a specific effect on MYC or on cell cycle phase-related transcription in general upon FBXO28 inhibition is difficult to evaluate since MYC seems to have a quite global role on regulation of the majority cell cycle regulated genes in cycling cells (as also pointed out by the reviewer). Indeed, cross-referencing our microarray data (FBXO28 KD) with publically available, cell cycle specific, gene expression and ChIP data suggest that around 3/4 of specific cell cycle-regulated genes are bound and regulated by MYC, as presented in a new figure (Supporting Information Fig. S5D). Depletion of FBXO28 significantly down regulated a subpopulation of MYC target genes (in particular in S, G2 and M-phases), while non-MYC target genes seemed less affected (2 non-MYC versus 33 MYC target genes, p=0.03). This demonstrates that FBXO28 KD preferentially affects MYC target genes but does not lead to a general down regulation of gene expression in S/G2/M phases of the cell cycle.

9. *Fig 5E, F, G- the extent of P300 binding and H4 acetylation should be determined at promoters that are not bound by Myc. This control would help determine how general the effects of FBXO28 depletion are. In addition, the authors should determine whether recruitment of GCN5, the other critical acetyltransferase bound by Myc, is also affected.*

We thank the reviewer for this suggestion and have now performed analysis of p300 and histone H4

acetylation in response to ΔF-FBXO28 expression on two genes not bound by MYC but with detectable p300 and H4Ac. The results (now presented as new Figures 5H and I) show that p300 and H4Ac association are not significantly changed under these conditions, again supporting the conclusion that the effects of FBXO28 on gene expression, p300 association and histone modification are not global but linked to a set of MYC-regulated genes.

Unfortunately we were not able to detect GCN5 association with the genes under study, due technical reasons.

10. *Fig. 5- For this and other figures the authors should provide p-values and indicate the number of biological replicates used.*

We have now added the requested information, the number of experiments and p-values, where applicable.

11. *Fig S5H - it appears that the FBXO28 inhibition results in increased ubiquitylation of the Myc K6R mutant. This needs to be explained.*

Yes, we have observed this a number of times, but have no clear-cut explanation for this. One possibility is that a competing E3 ligase(s) gets the chance to ubiquitylate the K6R mutant at other lysines when K6R-MYC is expressed together with ΔF-FBXO28. For instance, when p300 is no longer recruited to MYC as a result of K6R, MYC is expected to be deactylated at various lysines, making these sites available for ubiquitylation by another E3 ligase. However, this is highly speculative, and we find it outside the scope of the present investigation to address this question experimentally.

Reviewer #2

We are encouraged by the reviewer's view that our manuscript "*makes significant contributions to our understanding of ubiquitin ligases, the complex regulation of MYC, and suggests a role for FBXO28 in tumorigenesis……and adds an important new layer to the regulation of MYC, which should be of great interest to the readers of EMBO Molecular Medicine*". The reviewer points out two general issues. One relates to the correlation between high levels FBXO28 and poor survival in human breast cancer as well as the differentiation between effects on MYC regulated proliferation versus tumorigenicity. The other relates to the role of FBXO28 phosphorylation for the binding- and ubiquitylation of MYC.

Referring to the reviewer #2's comments in introductory part;

The role of FBXO28 in transformation and tumorigenesis is suggestive, but I believe the authors should be more cautious in interpretation of these results and need to consider alternative explanations, because: (1) It is not entirely unexpected that expression of deltaFbox-FBXO28 blocks colony formation (Fig. 8B) and reduces tumour volume (8D), since the authors have demonstrated extensively, that expression of deltaFbox-FBXO28 (or knock down of FBXO28) blocks cell proliferation of tumour cells. Any agent that blocks cell proliferation can be expected to have a similar effect. Thus, the specificity of these effects is unclear".

We agree with the reviewer that the role of FBXO28 on tumorigenesis should be more carefully discussed due to the implicit difficulties to separate the antiproliferative effects of FBXO28 inactivation (knockdown and/or ΔF-FBXO28) and the influence on MYC transformation. However, considering that our transformation assays quantify the number of MYC-induced colonies of

relatively the same size in the control compared to ΔF-FBXO28, we find that these data support a function for FBXO28 in MYC-induced transformation. We did not observe 'smaller' MYC-induced colonies in cells co-transfected with ΔF-FBXO28, indicating that the effect of ΔF-FBXO28 on proliferation per se is not a major factor in these p53 -/- MEF transformation assays. Furthermore, in parallel experiments we have analysed the effects of ΔF-FBXO28 on RAS (hrasG12V)-induced transformation, and in these experiments ΔF-FBXO28 had only minor effects on colony formation (unpublished observations), again speaking against that ΔF-FBXO28 attenuates transformation primarily due to its negative effects on proliferation. See also the response to reviewer #1, points 1, 7, 8 and 9, where the effects of FBXO28 on MYC versus general effects on transcription, proliferation, cell cycle, etc. are discussed. This is now mentioned in the result, page 16 and Supporting Materials and Methods.

The correlation between high nuclear fraction of pS344-FBXO28 and overall survival could be an indirect correlation, because pS344-FBXO28 is largely restricted to cells in S and G2/M phase (figure 2B) and a high nuclear fraction of pS344-FBXO28 may thus simply distinguish highly proliferative tumours from less proliferative ones. Although this doesn't diminish the utility of pS344-FBXO28 as a marker, by reading the manuscript I got the impression that the authors interpret these results as a causative relation. Careful rewriting of these sections and discussing these alternative interpretations could improve the manuscript.

We realize that this could have been described clearer. It was not our intention to seemingly over interpret the data, and we have now toned down that claim. However, we disagree that the data solely indicates that FBXO28 phosphorylation 'simply' reflects the proliferative status of the tumours. As shown in Fig. 8E, we do provide data demonstrating that analysis of FBXO28 phosphorylation separates patients according to survival also in the group of highly proliferative tumours (high Ki-67) and poorly differentiated (grade III) tumours.

Below are our responses to the specific points raised by the Reviewer:

(1) *The role of FBXO28 phosphorylation in its regulation should be addressed more rigorously, because it is mainly based on mutants of residue 344. The authors should immunopurify FBXO28 from synchronized cells in G1 (no phosphorylation) and S-phase or G2/M (high phosphorylation) and show MYC ubiquitylation in vitro similar to the experiment in Fig. 4G. If phosphorylation stimulates MYC ubiquitylation as suggested by the authors based on results with mutations, then the S or G2/M sample should be significantly more active.*

We acknowledge Reviewer #2 for his/her suggestion on how to address this experimentally and in fact we have tried to perform exactly the experiment proposed by the reviewer. However, our efforts to purify SCF^{FBXO28} complexes were partly prohibited due to the fact that phosphorylation also affects FBXO28 protein stability. We have now incorporated these new data in Fig. 2E, demonstrating that inhibition of CDK2 activity leads to proteasomal degradation of FBXO28 and that the phoshorylated-deficient S344A mutant is more unstable compared to the phosphorylation-mimicking S344E mutant (Fig. 2F). Our data also suggest that FBXO28 is auto-degraded by its associated SCF complex, as the F-box deletion mutant (ΔF) is significantly more stable (Fig. S2H).

Nevertheless, in an attempt to accommodate Reviewer #2's concern, we have used an alternative approach to address the role of FBXO28 activity specifically in S-phase. We now provide data demonstrating significant inhibitory effects by ΔF-FBXO28 on MYC ubiquitylation following release of cells from G1/S, whereas only a minor inhibitory effect was observed in a population of cells in G1 phase, indirectly linking the activity of FBXO28 towards MYC to progression in the cell cycle. The new data are shown in Fig S4E.

(2) *Related to point 1, I was somewhat surprised that the authors did not attempt to address whether FBXO28 phosphorylation affects recognition/binding of MYC. They show that SCF-FBXO28 formation is unaffected by phosphorylation, but to not attempt to demonstrate any effect on MYC*

binding. Cell lysates from synchronized cells as mentioned in point 1 could be used to address this question.

Referring to this comment, we have performed the suggested binding experiments by incubating extracts from ΔF-FBXO28 expressing cells (since ΔF-FBXO28 is stable in contrast to the wt protein, see Fig. S2G) prepared from different cell cycle phases together with MYC purified and bound to beads. These experiments show that ΔF-FBXO28 binds MYC throughout the cell cycle, although binding appears to be somewhat reduced in G1/early S phase (Fig S4F). In addition, by co-immunopurification using the non-phosphorylatable S344A-FBXO28 mutant, we show that this mutant binds MYC to the same extent as WT-FBXO28 (Fig S4G), supporting the conclusion that S344 phosphorylation is not required for FBXO28 to interact with MYC. These data also suggest that the binding of FBXO28 is regulated through a S344-independent mechanism, possibly involving phosphorylation or other modifications at additional sites, and that S344 phosphorylation rather plays a role in regulation of the ubiquitylation process, both with respect to auto-ubiquitylation (and self-destruction) of FBXO28 and non-proteasomal ubiquitylation of MYC and potentially other substrates. We thank the reviewer for this opportunity to bring in new important data to improve the manuscript. These new findings are discussed on pages 11 and 24 in the manuscript. Deeper understanding of how the exact binding between FBXO28 and substrates is regulated as well as how phosphorylation regulates the E3 ligase activity of the complex is the subject of an independent study.

(3) *Figure 5G: The authors demonstrate convincingly in figures 4H to J that FBXO28 does not affect MYC stability. However, in figure 5G (input panel) there seems to be a pretty significant increase in the steady state levels of MYC when ∆F-FBOXO28 is expressed. This internal inconsistency needs to be addressed somehow.*

We present multiple blots demonstrating that ΔF-FBXO28 (or FBXO28 KD) does not significantly affect MYC levels and it was unfortunate that in this particular experiment, ectopically expressed MYC appeared to be increased in the ΔF-FBXO28 expressing cells. This, however, was clearly not a reproducible result.

Minor points:

1. *Abstract and Introduction: "Here we identify a novel F-box....". "Novel" may not be correct as FBOXO28 has been identified previously, although not characterized.*

Now corrected

2. *Introduction: The introduction should mention the two recent landmark studies describing MYC as an amplifier of transcription programs (Cell, Volume 151, 2012: Nie et al. as well as Lin et al.)*

We apologize for this, these references are now added and discussed.

3. *page 9, last line: Reference for (Strohmaier et al. 2011) missing*

Now corrected

4. *page 10 top: (Fig. 2G)? I cannot find figure 2G*

Our mistake, we referred to Fig. 2E, now Fig. 2G in the new version.

5. *page 10, bottom: MYC turnover in the absence of FBXW7 (unpublished observations). This seems to be an important piece of data that may be worth showing in the supplementary figure section.*

The reason why this data was left out is because MYC protein is already stable in FBXW7-/- cells and FBXO28 does not further change MYC levels in these cells. However, as the reviewer suggested, we now provide data on MYC protein levels following FBXO28 knockdown or WT/ ΔF overexpression in FBXW7-/- cells (Fig S4I and J).

6. *Figure 4A: based on this figure the authors cannot conclude that FBXO43, FBXO5, FBXO16 do not interact with MYC. The expression levels for these F-box proteins are very low compared to FBXO28 and one would not expect to detect a signal at the exposure level shown even if they interacted with MYC. The authors should either mention that in the text, show a longer exposure, or remove these 3 F-box proteins from this figure.*

As suggested by the reviewer, we now show a longer exposure of the blot, or if requested, we can remove this figure. In addition, we present interaction data on MYC-SKP2 and have also included one additional F-box protein with a significant effect on cell proliferation (FBXO22). See also the response to reviewer #1, point 3. Hope this is satisfactory but since both reviewer's had comments on this figure we will ultimately leave this issue up to the Editor's judgement.

7. *Figure 4G should explain S/E and S/A in the figure legend*

Now corrected

8. *page 17: OS has not been defined.*

Now corrected

9. *page 24, line 4: comma before cycloheximide*

Now corrected

10. *page 25, line 1: performed*

Now corrected

11. *The authors need to be consistent with their labelling of figures. They mostly use ∆F-FBOXO28, but for example figures 5C and E use FBOXO28-dF or dF-FBOXO28.*

Now corrected

12. *Figure 7B: The figure legend should give information about what level of overexpression is considered high, etc. What was the criterion?*

We are sorry that this was not clear. This is now described in greater detail in the Figure legend.

13. *Figure 7C: The table could be moved to the supplementary information. It is not very easy to understand, and the data results are nicely summarized in the text already.*

Figure 7C has now been moved to Supporting information Table S3.

14. *Figure 8 legend: panel C states "Analysis was performed as in C." Should probably read ...as in B?*

Yes, now corrected.

We thank the reviewers for their suggestions that we feel have led to a much-improved manuscript.

2nd Editorial Decision 29 April 2013

Thank you for the submission of your revised manuscript to EMBO Molecular Medicine. We have now received the enclosed reports from the Reviewers that were asked to re-assess it. As you will see the reviewers are now supportive and I am pleased to inform you that we will be able to accept your manuscript pending the following final amendments:

1) Reviewer 2 notes that the FBXO22 lane in Fig 4a appears delimited by vertical discontinuities in the background that are consistent with a splice-in. Our in-house image expert and I agree. Could you please provide us with your explanation/interpretation and the source images for this experiment and amended figure (with lanes clearly separated where appropriate with explanation in the legend)? Could you also please comment on this Reviewer's remark on the experiment?

2) As per our Author Guidelines, the description of all reported data that includes statistical testing must state the name of the statistical test used to generate error bars and P values, the number (n) of independent experiments underlying each data point (not replicate measures of one sample), and the actual P value for each test (not merely 'significant' or 'P < 0.05').

Please submit your revised manuscript, your comments and the source data within two weeks. Needless to say, the sooner we receive it the sooner I will be able to formally accept your manuscript.

I look forward to reading a new revised version of your manuscript as soon as possible.

***** Reviewer's comments *****

Referee #1 (General Remarks):

I have read the authors' response and the revised manuscript and feel that they have adequately addressed my major initial concerns. In my opinion the paper is now acceptable for publication.

Referee #2 (General Remarks):

The authors addressed all my points and I support publication of the manuscript.
The only comment I have is regarding figure 4a. Unless this is a pdf conversion issue, the file I am looking at looks like the FBXO22 lane was spliced into that figure. If the authors chose to keep this figure they need to clearly indicate (with lines in the figure and explain in the legend) that the lane with FBXO22 was spliced in. In any case, I am not completely convinced by these results (vastly different expression levels and co-migration with IgG makes interpretation difficult, etc.), but the authors qualify the interpretation in the text.

2nd Revision - authors' response 09 May 2013

We are pleased that the reviewers are satisfied with our revised version of the manuscript and that it is now acceptable for publication in EMBO Molecular Medicine.

Below please find the requested comments regarding Reviewer #2's final observations and editorial remarks.

1) *Reviewer 2 notes that the FBXO22 lane in Fig 4a appears delimited by vertical discontinuities in the background that are consistent with a splice-in. Our in-house image expert and I agree. Could you please provide us with your explanation/interpretation and the source images for this experiment and amended figure (with lanes clearly separated where appropriate with explanation in the legend)? Could you also please comment on this Reviewer's remark on the experiment?*

   Referee #2 (General Remarks):
   The only comment I have is regarding figure 4a. Unless this is a pdf conversion issue, the file I am looking at looks like the FBXO22 lane was spliced into that figure. If the authors chose to keep this figure they need to clearly indicate (with lines in the figure and explain in the legend) that the lane with FBXO22 was spliced in. In any case, I am not completely convinced by these results (vastly different expression levels and co-migration with IgG makes interpretation difficult, etc.), but the authors qualify the interpretation in the text.

We have now modified Figure 4A, with evident separation of the cropped lanes and clarification in the figure legend. We are also attaching the source blots used to produce the images.

The reason why the lanes were cropped from the original blots was that in our interaction studies we found that another F-box protein also associates with MYC protein (as shown in the source blot). Given that work on this particular F-box protein is currently being carried out in our lab, we decided to exclude these results from this manuscript. In order to incorporate FBXO22 and SKP2/FBXL1 into the figure (as previously requested by Reviewer #1), and exclude this new F-box protein, we had to crop and join non-adjacent lanes from the original blot.

We agree with the reviewer that the uneven expression levels of the different F-box proteins studied and the IgG background are a problem, and that some lanes give inconclusive information. However, by providing equal exposures of the input and coIP blots it is evident from bands with stronger input intensity where no sign of a band is apparent in the coIP blot that not "all" F-box proteins bind MYC. The specific interaction between FBXO28 and MYC is further supported by Figures 4B-D and S4.

2) *As per our Author Guidelines, the description of all reported data that includes statistical testing must state the name of the statistical test used to generate error bars and P values, the number (n) of independent experiments underlying each data point (not replicate measures of one sample), and the actual P value for each test (not merely 'significant' or 'P < 0.05').*

Information on statistical tests used to generate p-values is now reported in the corresponding figure

legends, and actual p-values have been added to the figures, where appropriate.

Once again, we thank the reviewers and the editor for their prompt re-evaluation of the manuscript, and we hope that this final version is suitable for publication.
