# Supplementary material for: CDK-mediated activation of the SCFFBXO28 ubiquitin ligase promotes MYC-driven transcription and tumourigenesis and predicts poor survival in breast cancer
Source: EMBO Mol Med. 2013 Jun 14;5(7):999–1018. doi: 10.1002/emmm.201202341 (PMC3721474; doi:10.1002/emmm.201202341)
Supplement: Supplementary file 2 [file emmm0005-0999-SD2.pdf]

SUPPORTING INFORMATION

CDK-mediated activation of the SCF^{FBXO28} ubiquitin ligase promotes MYC-driven transcription and tumorigenesis and predicts poor survival in breast cancer

Diana Cepeda,[1,2] Hwee-Fang Ng,[1,15] Hamid Reza Sharifi,[3,15] Salah Mahmoudi,[1,15] Vanessa Soto Cerrato,[4,16] Erik Fredlund,[3,16] Kristina Magnusson,[6,16] Helén Nilsson,[3,16] Alena Malyukova,[1,2] Juha Rantala,[6] Daniel Klevebring,[7] Francesc Viñals,[8] Nimesh Bhaskaran,[1] Siti Mariam Zakaria,[3] Suryo Rahmanto,[1] Stefan Grotegut,[9] Michael Lund Nielsen,[10] Cristina Al-Khalili Szigyarto,[11] Dahui Sun,[9] Mikael Lerner,[2] Sanjay Navani,[6] Martin Widschwendter,[12] Mathias Uhlén,[11] Karin Jirström,[13] Fredrik Pontén,[6] James Wohlschlegel,[14] Dan Grandér,[2] Charles Spruck,[9] Lars-Gunnar Larsson,[3*] and Olle Sangfelt[1,2,*]

TABLE OF CONTENTS

SUPPORTING INFORMATION FIGURES **page**

Figure S1……………………………………………………………………...3

Figure S2……………………………………………………………………...5

Figure S3……………………………………………………………………...8

Figure S4……………………………………………………………………...10-11

Figure S5……………………………………………………………………...14-15

Figure S6……………………………………………………………………...18

Figure S7……………………………………………………………………...19

SUPPORTING INFORMATION TABLES

Table S1 ……………………………………………………………………. 21

Table S2 ……………………………………………………………………. 22

Table S3 ……………………………………………………………………. 23

Table S4 ……………………………………………………………………... 23

Table S5A …………………………………………………………………….24

Table S5B …………………………………………………………………….25

Table S5C …………………………………………………………………….25

Table S6A …………………………………………………………………….26

Table S6B …………………………………………………………………….27

Table S6C …………………………………………………………………….27

SUPPORTING INFORMATION MATERIALS AND METHODS ………... 28

SUPPORTING INFORMATION REFERENCES …………………………… 42

SUPPORTING INFORMATION - FIGURES

Supporting information Figure S1

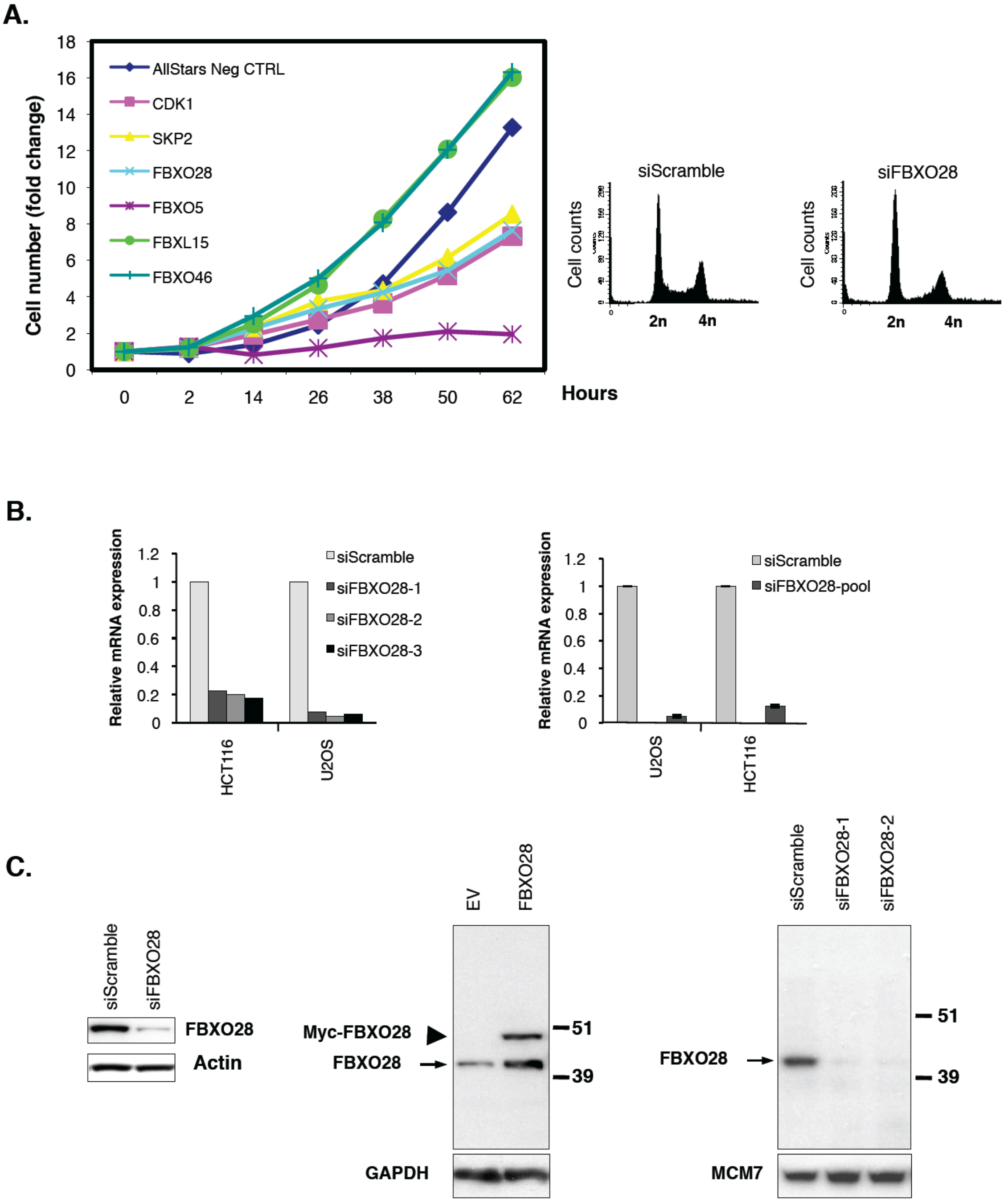

Supporting information Figure S1. (A) Inhibition of cell proliferation upon FBXO28 depletion. Left panel: Live cell microscopy analysis in HCT116 cells reverse transfected with the indicated siRNA for 62 hours. The number of cells was determined on 2 hours intervals using an automated cell growth image analysis platform (Incucyte HD v1.5). Increase in cell number was determined as fold change and compared with non-targeting and CDK1 targeting siRNA serving as negative and positive controls, respectively. Right panel: Flow cytometry analysis of HCT116 cells transfected with FBXO28 siRNAs, or scrambled control siRNA. DNA content was visualized by propidium iodide (PI) staining. **(B)** Knockdown efficiency of FBXO28 siRNAs. HCT116 and U2OS cells were transfected with either three individual siRNA oligos (left panel) or a pool of the three oligos (right panel) targeting FBXO28, or non-targeting control oligos (siScramble). Relative *FBXO28* mRNA expression was compared to siScramble and analyzed by qRT-PCR and normalized t*o β-ACTIN*. Each data are representative of three independent experiments and is shown as mean ± SEM. **(C)** Specificity of the FBXO28 antibody. Left panel: Immunoblot (IB) analysis documenting the effect of FBXO28 siRNA transfection in U2OS cells. Middle panel: U2OS cells were transfected with Myc-tagged FBXO28 expression construct, or an empty control vector (EV) and analyzed by IB analysis with anti-FBXO28 rabbit polyclonal antibodies. Ectopically expressed FBXO28 (arrowhead) and endogenous FBXO28 (arrow) proteins are indicated. GAPDH was used as loading control. Right panel: U2OS cell extracts transfected with two different *FBXO28* siRNA oligos or siScramble for 48 hours were subjected to IB analysis using FBXO28 antibody and anti-MCM7 as a loading control. The position of endogenous FBXO28 protein is indicated by an arrow.

Supporting information Figure S2

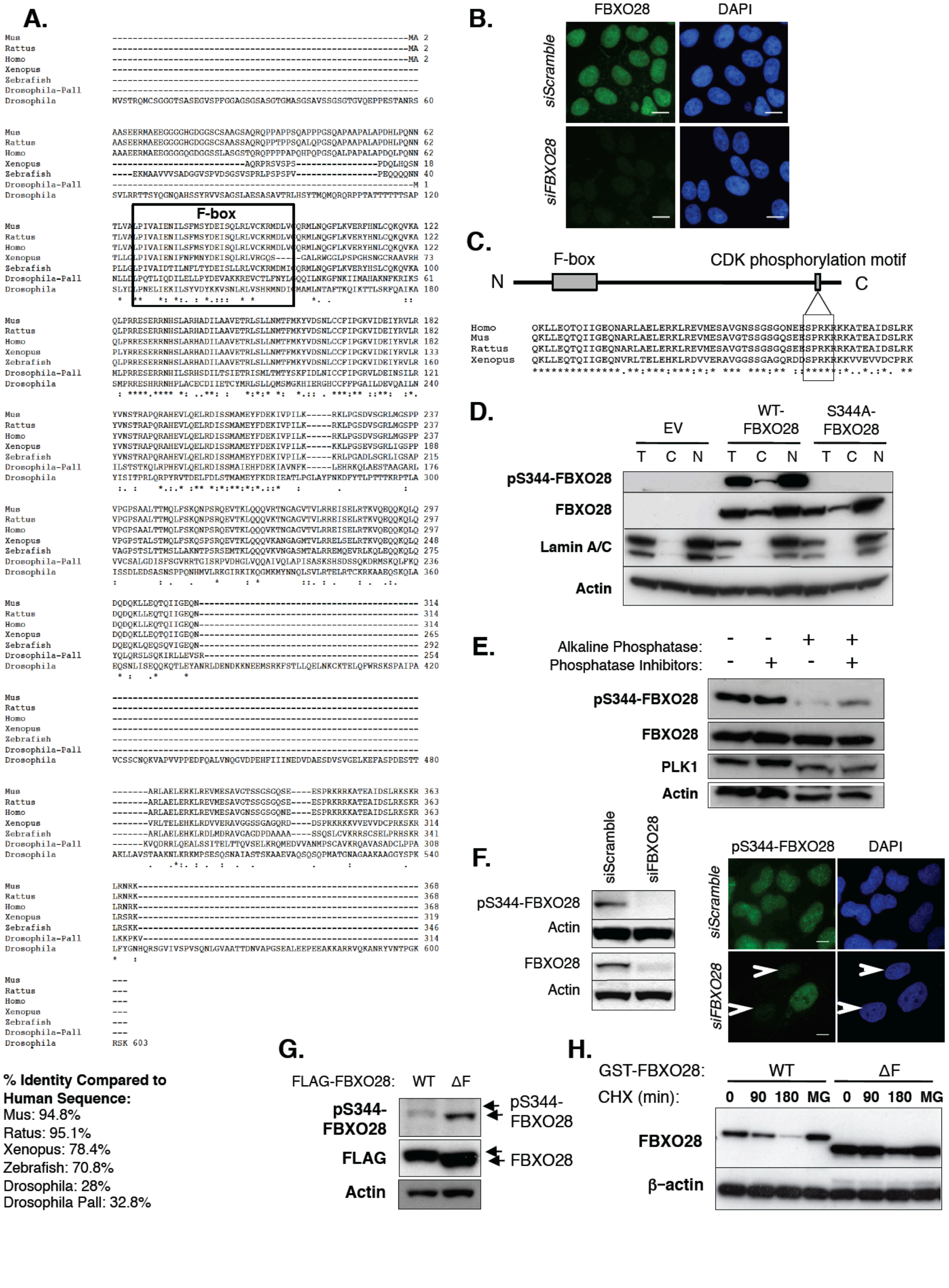

% Identity Compared to Human Sequence:
Mus: 94.8%
Ratus: 95.1%
Xenopus: 78.4%
Zebrafish: 70.8%
Drosophila: 28%
Drosophila Pall: 32.8%

Supporting information Figure S2. (A) FBXO28 protein is evolutionary conserved. Amino acid sequence alignment of human FBXO28 with Mouse, Rat, Xenopus, Zebrafish, and two Drosophila FBXO28 orthologs, using the ClustalW program (http://www.ebi.ac.uk/Tools/msa/clustalw2). F-box domain is boxed (aa 67-94). **(B)** Specificity of the FBXO28 antibody as analysed by immunoflourescence (IF) microscopy. U2OS cells were transfected with scrambled control siRNA (upper panel) or FBXO28 specific siRNA oligos (lower panel). DNA was counterstained with DAPI. Scale bar: 10 μm. **(C)** Schematic diagram of FBXO28 amino acid sequence surrounding serine-344. The amino-terminal (N) F-box domain and the position of the putative CDK motif at the carboxy-terminus (C) in FBXO28 are indicated (Upper panel). Sequence (Clustal W) alignments of the consensus CDK phosphorylation motif (boxed) in different species (Lower panel). **(D)** U2OS cells were transfected with GST-WT-FBXO28, GST-S344A-FBXO28, or GST-EV. Nuclear (N), cytosolic (C) and total (T) extracts were subjected to IB analysis with the indicated antibodies (Upper panel). b-actin was used as a control for loading. **(E)** Whole cell extracts from U2OS cells were treated with calf intestinal alkaline phosphatase with and without phosphatase inhibitors for 1 hr at 37ºC and analyzed by IB using anti-pS344-FBXO28 and anti-FBXO28 antibodies, respectively. Immunoblots were probed with anti-PLK1 antibodies as a control for the efficiency of the phosphatase treatment. b-actin was used as loading control. **(F)** Specificity of the pS344-FBXO28 antibody as confirmed by siRNA-mediated FBXO28 knockdown. Left panel: IB of whole cell extracts from U2OS cells transfected with a pool of 3 different FBXO28 specific siRNAs or a scambled control siRNA. Phosphorylation and expression of FBXO28 was assessed after probing two separate

filters using anti-pS344-FBXO28 and anti-FBXO28 antibodies, respectively. b-actin was used as a control for loading. Right panel: U2OS cells were transfected with control siRNAs or FBXO28 siRNAs and stained by indirect immunoflourescence analysis using pS344-FBXO28 antibodies. Nuclei was visualised with DAPI staining. Arrows indicate cells with loss of pS344-FBXO28 signal, demonstrating specificity of the pS344-FBXO28 antibody. **(G)** U2OS cell lysates expressing the indicated Flag-tagged FBXO28 constructs were immunoblotted with pS344-FBXO28-specific antibodies to verify phosphorylation of ΔF-FBXO28. FLAG and Actin antibodies were used as expression and loading controls, respectively. **(H)** Cycloheximide (CHX) chase analysis of WT and ΔF-FBXO28 in Cos7 cells. Where indicated, chased cells were treated with MG-132 (MG) for 4 hours.

Supporting information Figure S3

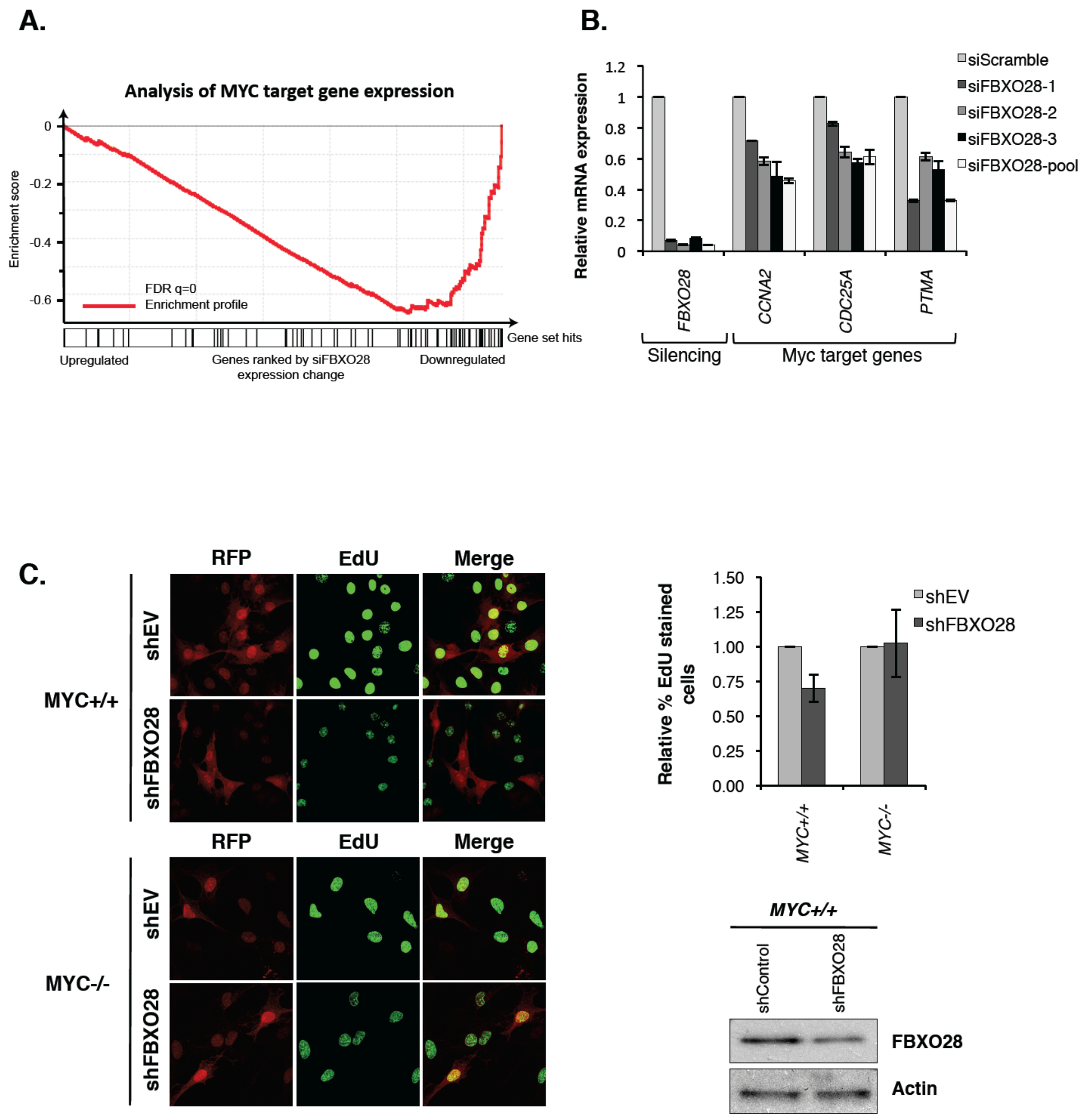

Supporting information Figure S3. (A) Downregulation of MYC target genes upon depletion of FBXO28 by siRNA transfection in HCT116 cells. GSEA analysis of gene expression changes after 36 hours of FBXO28 siRNA transfection as described in Figure 3C. p <0.005. **(B)** Downregulation of MYC target genes in response to FBXO28

depletion by siRNA. U2OS cells were transfected with scrambled control siRNA or individual FBXO28 siRNA oligos (siFBXO28-1, -2, or -3) or a combination of the three different oligos (siFBXO28-pool). 48 hours after transfection, total RNAs were extracted and used for qRT-PCR with gene-specific primers for the indicated MYC target genes. FBXO28 knockdown efficiency is shown (left). **(C)** *MYC+/+* and *MYC-/-* Rat1 cells transduced with doxycyclin-inducible TRIPZ lentiviral vectors co-expressing RFP and FBXO28 shRNA or TRIPZ empty vector control (shEV) were incubated with EdU before fixation and staining for fluorescence microscopy. Left panels: Immunofluorescence analysis of a representative cell population showing RFP, EdU staining, and a merged image. Right, upper panel: Bright RFP-positive cells were counted and scored for EdU staining. For each cell line, the shFBXO28 cell counts were normalized to the counts in shEV control cells. Error bars represent the SEM of two independent counts. Right, lower panel: Western blot analysis verifying silencing of FBXO28 in *MYC+/+* cells.

Supporting information Figure S4

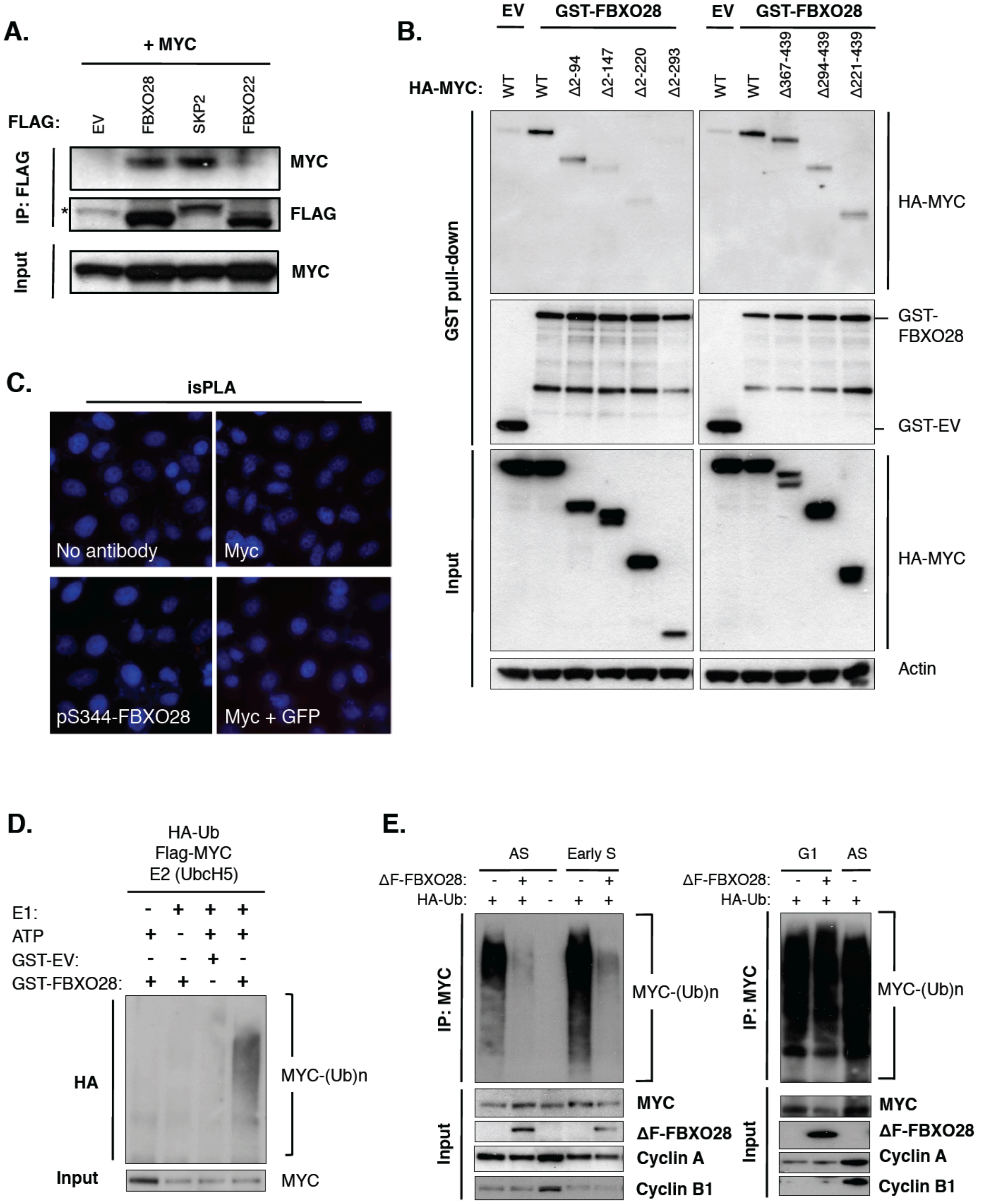

Supporting information Figure S4

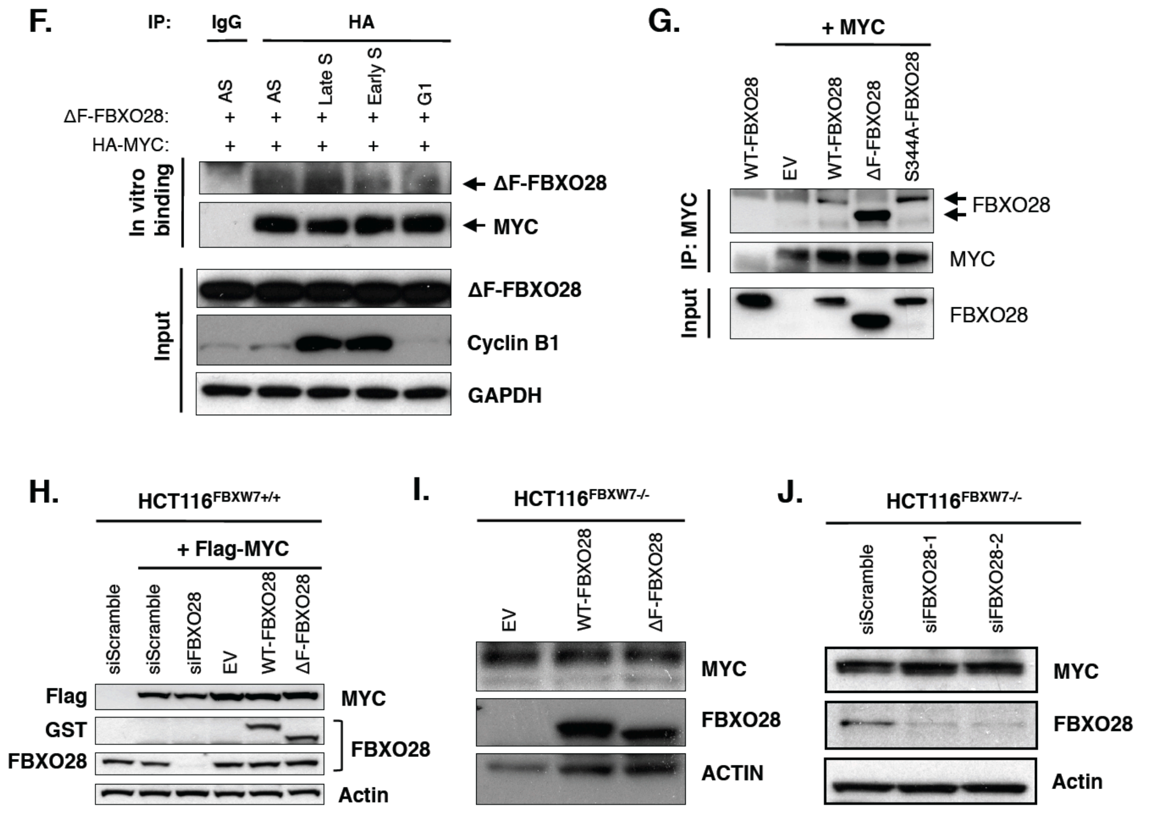

Supporting information Figure S4. **(A)** MCF-7 cell extracts cotransfected with the indicated expression constructs were immunoprecipitated with FLAG antibodies followed by immunoblotting with MYC (N262) antibody as indicated. Whole cell extracts were IB with MYC antibodies to assess input levels. **(B)** FBXO28-MYC interaction *in vivo*. Upper panel: Cos7 cells were co-transfected with GST-FBXO28, or an empty vector (EV), together with HA-MYC-WT or a panel of MYC deletion constructs as indicated. Whole cell extracts were used in GST pull-down assays, resolved by SDS-PAGE and immunoblotting as depicted. 5% of the cell extract was used as input and probed with anti-HA and anti-Actin antibodies. (**C**) Control conditions used for in

situ proximity ligation assay (isPLA) for the detection of MYC-FBXO28 interaction. The panels depict controls omitting primary antibodies, applying only one of two primary antibodies or one antibody of interest together with a negative control primary antibody (GFP), respectively. Cells were counterstained with Hoechst for visualization of nuclei. (**D**) Ubiquitylation of MYC protein *in vitro* was performed as described in Materials and Methods of Supporting information, and ubiquitylation was detected by immunoblotting using anti-HA antibody**.** *In vitro* reactions were performed without E1 enzyme, or ATP, as negative controls. **(E)** U2OS cells expressing Dox-induced ΔF-FBXO28 and cotransfected with HA-Ub, where indicated, were synchronized in different cell cycle phases; Early S: 2 hrs release from a double thymidine arrest (left panel); G1: 4 hrs nocodazole release (right panel). Lysates from synchronized or asynchronous (AS) cells were subjected to *in vivo* ubiquitylation assays using anti-MYC antibodies (N262) and poly-ubiquitylated MYC was detected by immunoblot analysis using anti-HA antibodies . Whole cell lysates were IB with MYC (C33), FBXO28 (for detection of ΔF-FBXO28), Cyclin A and cyclin B1 antibodies. **(F)** For *in vitro* interaction assays, Dox-induced U2OS cells expressing ΔF-FBXO28 were synchronized in different cell cycle phases (Early S: 2 hrs release from a double thymidine arrest; Late S: 6 hrs release from a double thymidine arrest; G1: 4 hrs nocodazole release). Synchronized cells or asynchronous cell populations (AS) were lysed and protein extracts were incubated for 2 hours with HA-MYC (previously immunopurified from HEK293 cells and bound to beads) to allow for protein complexes to form. Interactions between MYC and FBXO28 were detected by IB analysis using anti-FBXO28 and MYC antibodies. Whole cell extracts from the U2OS cells were blotted with FBXO28, Cyclin B1 and GAPDH as input controls. **(G)** Extracts

from HCT116 cells cotransfected with the indicated expression constructs were immunoprecipitated with MYC antibodies and immunoblotted with FBXO28 or MYC antibodies, as indicated. Whole cell extracts were blotted with FBXO28 antibodies as input control. **(H)** HCT116 cells were cotransfected with the indicated expression constructs or siRNAs for 48 h and MYC protein levels were analysed by IB as shown. **(I)** Lysates from HCT116 *FBXW7-/-* cells cotransfected with the indicated expression constructs or **(J)** siRNAs for 48 h were immublotted with anti-MYC antibodies, as shown. FBXO28 antibodies were used to show expression or silencing and Actin levels were used for loading.

Supporting information Figure S5

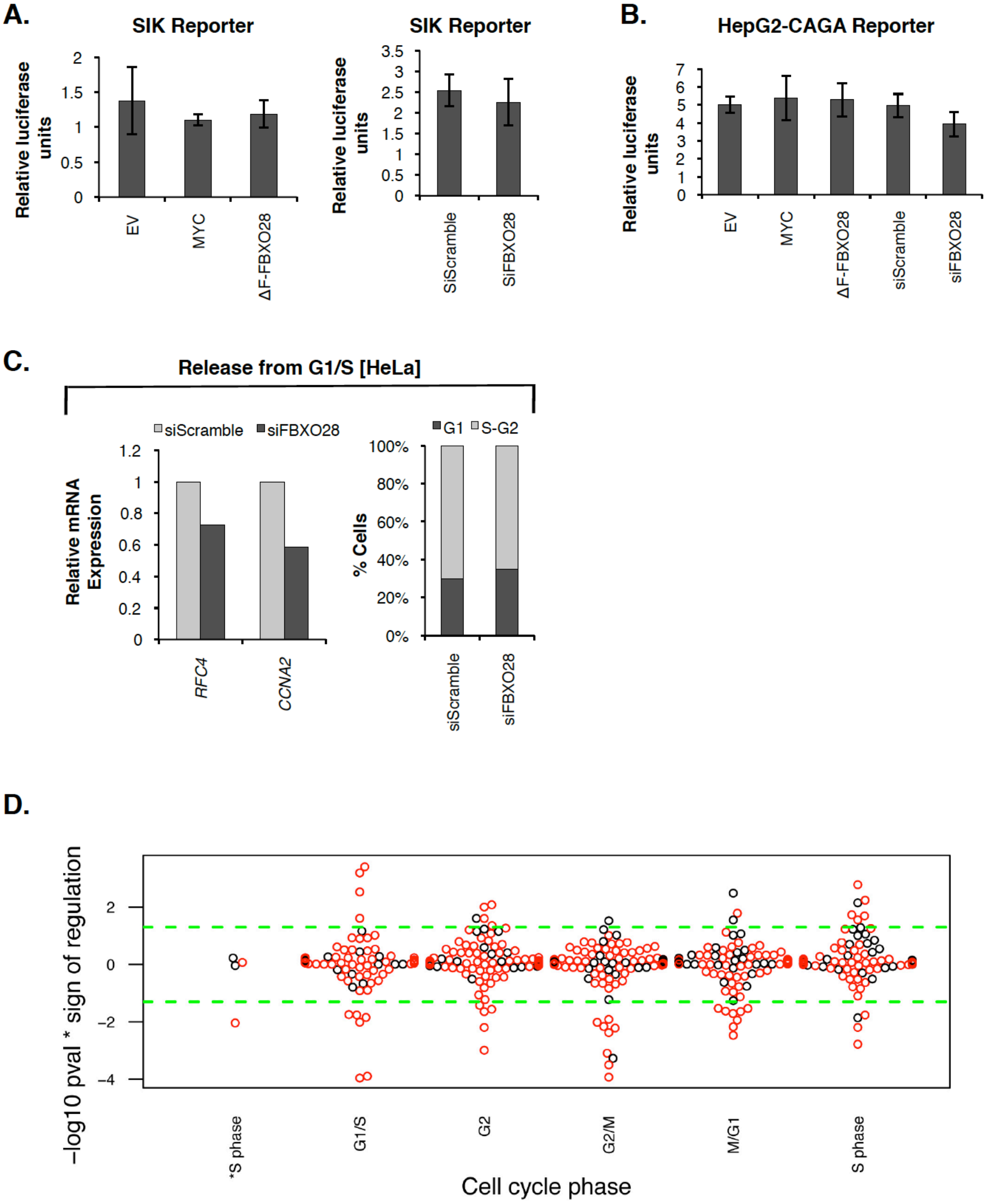

Supporting information Figure S5

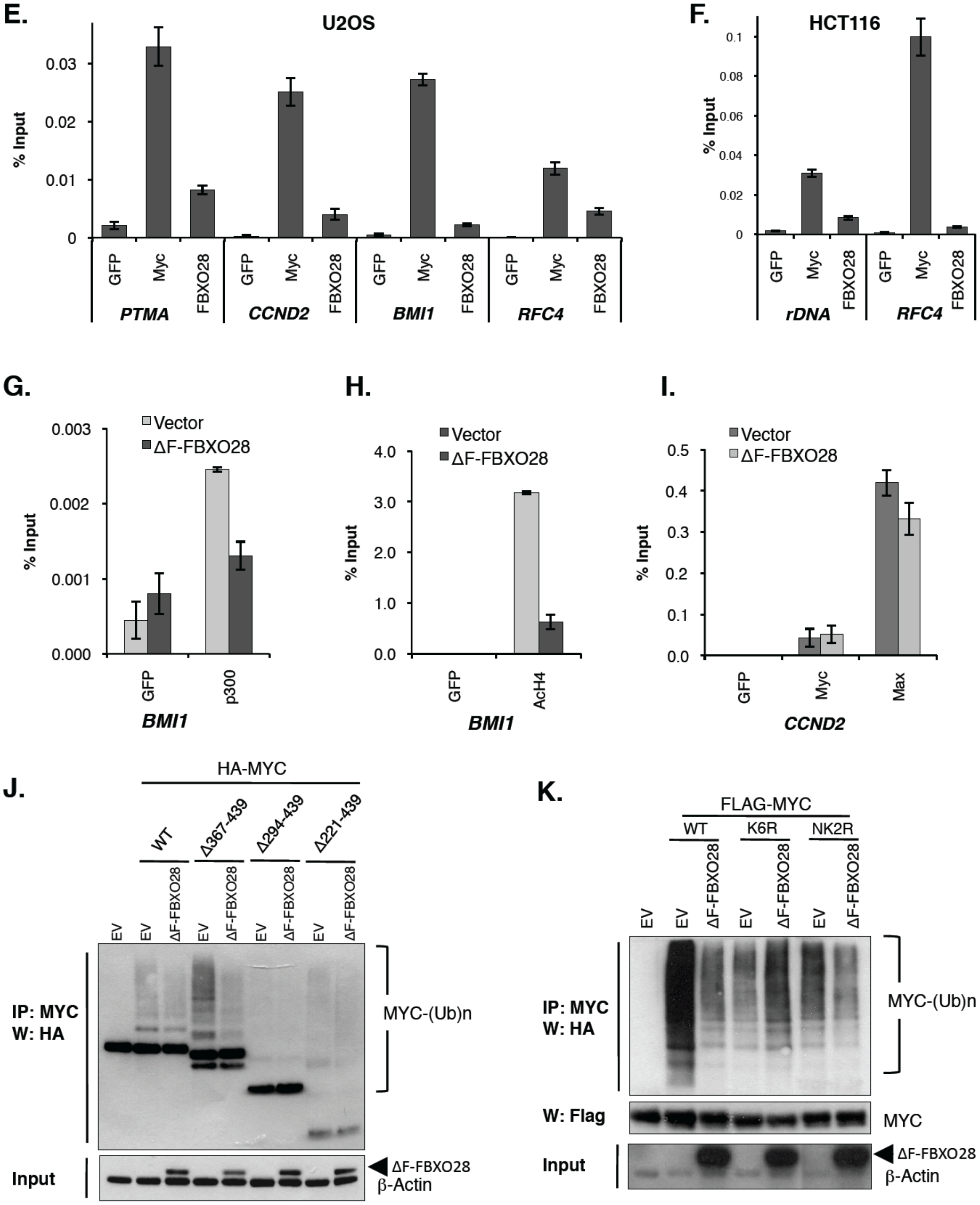

Supporting information Figure S5. (A) Luciferase assay in HeLa cells transfected with the salt-induced kinase (SIK) gene luciferase reporter for 48 hours, in combination with the indicated expression constructs or siRNAs. Cells were cotransfected with Renilla control for normalization of luciferase activity. The data represent the mean ± standard error of the mean (SEM) for three independent experiments. **(B)** Measurement of luciferase activity in HeLa cells transfected with the HepG2-CAGA reporter construct containing 4 SMAD-binding sites, for 48 hours. Cells were treated with TGFβ (0.5ng/ml) overnight prior to measurements. Analysis was performed as in (A). **(C)** Left: qRT-PCR analysis in HeLa cells documenting expression changes of the MYC target genes *RFC4* and *CCNA2* upon depletion of FBXO28 using siRNAs. HeLa cells were transfected with FBXO28 or Scrambled control siRNAs prior to synchronization at G1/S by a double thymidine block and release into S-phase for 4 h. Triplicate measurements were normalized to *β-ACTIN* expression and data is representative of two experiments. Right: Cell cycle distributions were analyzed by flow cytometry. **(D)** Plot of gene expression data relating to cell cycle phases as defined by Whitfield et al. (Whitfield et al, 2002). Differential expression between cells transfected with siFBXO28 and grown for 36 hours and cells transfected with a non-specific siRNA is shown as a negative corrected log10 p-value. The sign on the y-axis represents upregulation (positive sign) or downregulation (negative sign) as a consequence of siFBXO28 treatment. Genes known to be bound by MYC in K562 cells (http://genome.ucsc.edu/ENCODE/analyses) are shown as red rings, whereas non-MYC bound genes are in black. The green dotted lines reprent a corrected p-value of 0.05 (log10 0.05 = 1.3). **(E)** Representative Q-ChIP experiment for the association of FBXO28 protein at the *PTMA*, *CCND2*, *BMI1*, and *RFC4* gene promoters

in U2OS cells. GFP antibody was used as a negative control. (**F**) Q-ChIP for the association of FBXO28 protein at the *rDNA* and *RFC4* gene promoters in HCT116 cells. (**G**) Overexpression of ΔF-FBXO28 reduces p300 binding at MYC target promoters. Enrichment of p300 at the *BMI1* gene promoter was measured by Q-ChIP assays following doxocycline-induced expression of ΔF-FBXO28 in U2OS cells or empty-vector control cells. (**H**) Enrichment of acetylated histone H4 at the *BMI1* gene promoter was measured as in (G). **(I)** Q-ChIP assay with the indicated antibodies at the *CCND2* gene promoter upon doxycycline-induced ΔF-FBXO28 expression in U2OS cells. For all of the Q-ChIP experiments in **(F-J)**, GFP antibody was used as a negative control. Bars represent protein enrichment at the indicated gene promoters analyzed by qPCR in two independent experiments, and normalized to total DNA input. (**J**) *In vivo* ubiquitylation assays performed in U2OS cells co-transfected with the depicted HA-tagged C-terminal MYC deletion constructs together with empty vector (EV) or FLAG-ΔF-FBXO28, along with HA-Ubiquitin. MYC was immunoprecipitated with anti-MYC antibodies (N262) and poly-ubiquitylated MYC was detected by anti-HA immunoblot analysis. Whole cell lysates were immunoblotted using FBXO28 and β-Actin antibodies respectively. (**K**) *In vivo* ubiquitylation assays in U2OS cells co-transfected with FLAG-tagged WT-MYC, a construct containing point mutations at six lysines just upstream of the basic region of MYC (K6R), or having mutations at two lysines within the MBII region of MYC (NK2R), along with either EV or ΔF-FBXO28. All cells were cotransfected with HA-Ubiquitin. Poly-ubiquitylated MYC was immunoprecipitated using MYC antibodies and detected by immunoblotting using anti-HA antibody. Input lysates were immunoblotted using MYC, FBXO28 and β-Actin.

Supporting information Figure S6

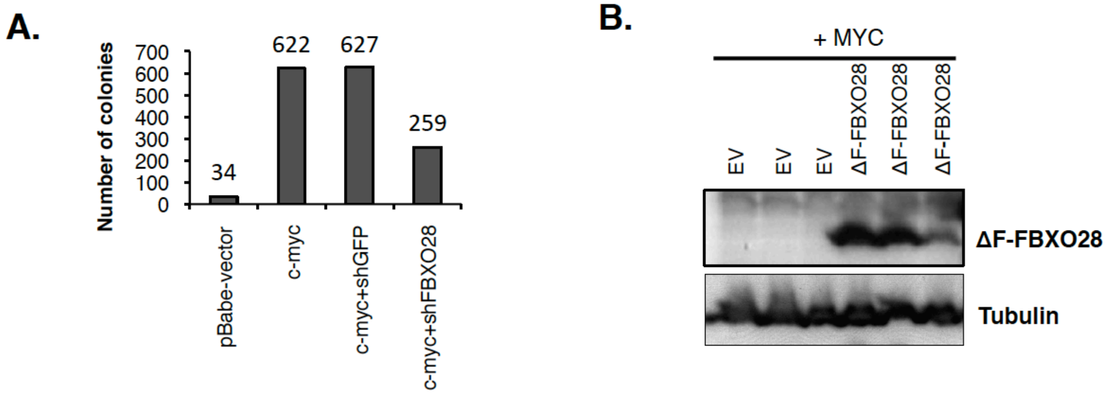

Supporting information Figure S6. (A) *P53-/-* MEF cells were embedded in soft-agar medium after co-transduction with shFBXO28 vectors or a shRNA-GFP control vector and MYC, as indicated. Tranduction with empty pBabe vector alone was used as a negative control. Colonies were scored after 21 days. The number of colonies from a representative assay is shown above each bar. **(B)** ΔF-FBXO28 expression in tumor specimens resected from the sacrificed mice at day 22 as outlined in Figure 6G. ΔF-FBXO28 expression was analyzed by IB analysis using anti-FBXO28 antibodies. Shown are representative blots of three control mice (EV) and three ΔF-FBXO28-expressing mice. Tubulin was used as a loading control.

Supporting information Figure S7

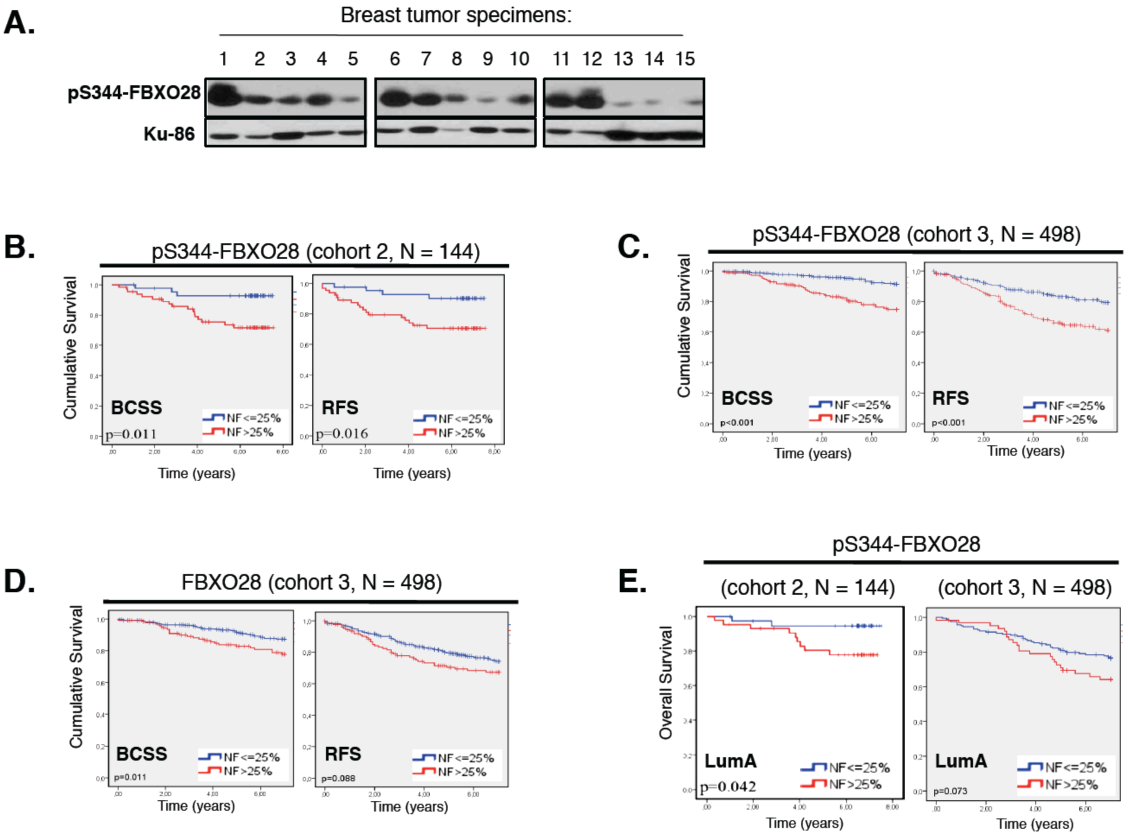

Supporting information Figure S7. (A) IB analysis was performed in a panel of primary breast tumor specimens (cohort 1). 50 µg total protein was separated by SDS-PAGE and probed with anti-pS344-FBXO28 antibody and anti-Ku86 for comparison. Note the differential FBXO28 phosphorylation levels (upper bands) in specific tumor samples. (**B**) Kaplan-Meier plots of breast cancer specific survival (BCSS) (left panel) and relapse free survival (RFS) (right panel) of breast cancer patients (n=144, cohort 2) stratified according to the nuclear fraction (NF) of phosphorylated FBXO28. Immunohistochemistry was performed using the anti-S344-FBXO28 antibody. (**C**) Kaplan-Meier plots of BCSS (left panel) and RFS (right panel) in breast cancer patients

(n=498, cohort 3) stratified according to the nuclear fraction (NF) of phosphorylated FBXO28. Immunohistochemistry was performed using the anti-S344-FBXO28 antibody. (**D**) Kaplan-Meier plot of BCSS (left panel) and RFS (right panel) in breast cancer patients (n=498, cohort 3) stratified according to the nuclear intensity (NI) of FBXO28 protein. A log-rank test was used to show differences between groups with low versus high FBXO28 nuclear staining intensity. Immunohistochemistry was performed using the anti-FBXO28 pan antibody detecting the total pool of FBXO28 protein. (**E**) Kaplan-Meier plot of overall survival (OS) in breast cancer patients classified as luminal A molecular subtype and stratified according to the nuclear fraction (NF) of phosphorylated FBXO28 in cohort 2 (left panel) and cohort 3 (right panel), respectively. For all analyses in **(B-E)**, a log-rank test was used to show differences between groups using 25% NF as cut-off. $p < 0.05$.

SUPPORTING INFORMATION - TABLES

Supporting information Table S1. Related to Figure 1.

Top ranked F-box genes that reduced cell proliferation in KPL4 cells upon RNAi in the functional screen shown in Figure 1B.

RANK	[a] GENE (N = 6,135)
17	FBXO28
29	FBXL4
36	FBXO4
41	FBXO2
53	FBXO46
61	FBXL5
62	FBXO3
106	FBXL7
163	FBXO40

[a] druggable genome siRNA library v1.0 (Qiagen Ambion, Inc)

Reduced cell proliferation was monitored by automatic fluorescence microscopic analysis of Ki-67 expression

Supporting information Table S2.

FBXO28 is Overexpressed in Primary Breast Cancer.

Study	Tumor Type	Reference
1	Ductal breast carcinoma	Bittner Breast, *Not Published, 2005*
2	Ductal breast carcinoma - High grade	Bittner Breast, *Not Published, 2005*
3	Ductal breast carcinoma epithelia - Dead at 1 year	Boersma Breast, *Int J Cancer, 2008*
4	Breast carcinoma - High grade	Desmedt Breast, *Clin Cancer Res, 2007*
5	Ductal breast carcinoma Invasive - Recurrence at 3 years	Desmedt Breast, *Clin Cancer Res, 2007*
6	Ductal breast carcinoma Invasive - High grade	Ginestier Breast, *Clin Cancer Research 2006*
7	Breast carcinoma - Advanced N stage	Ivshina Breast, *Cancer Res, 2006*
8	Breast carcinoma - High Elston Grade	Ivshina Breast, *Cancer Res, 2006*
9	Breast carcinoma - Recurrence at 3 years	Loi Breast, *J Clin Oncol, 2007*
10	Breast carcinoma - Recurrence at 5 years	Loi Breast, *J Clin Oncol, 2007*
11	Ductal breast carcinoma	Lu Breast, *Breast Cancer Res Treat, 2008*
12	Ductal breast carcinoma - High Bloom-Richardson grade	Lu Breast, *Breast Cancer Res Treat, 2008*
13	Lobular breast carcinoma - Advanced N stage	Lu Breast, *Breast Cancer Res Treat, 2008*
14	Ductal breast carcinoma epithelia - High grade	Ma Breast 2, *Cancer Cell, 2004*
15	Ductal breast carcinoma - High grade	Ma Breast 3, *Cancer Cell, 2004*
16	Breast carcinoma - Dead at 5 years	Pawitan Breast, *Breast Cancer Res, 2005*
17	Ductal breast carcinoma vs. normal	Richardson Breast 2, *Cancer Cell, 2006*
18	Invasive breast carcinoma - Metastatic event at 3 years	Schmidt Breast, *Cancer Res, 2008*
19	Breast carcinoma - High grade	Sotiriou Breast 3, *J Natl Cancer Inst, 2006*
20	Breast carcinoma – Recurrence at 1 year	Sotiriou Breast 3, *J Natl Cancer Inst, 2006*
21	Breast carcinoma – Recurrence at 3 years	Sotiriou Breast 3, *J Natl Cancer Inst, 2006*
22	Breast carcinoma – Recurrence at 5 years	Sotiriou Breast 3, *J Natl Cancer Inst, 2006*
23	Invasive ductal breast carcinoma vs. normal	Turashvili Breast, *BMC Cancer, 2007*
24	Invasive lobular breast carcinoma vs. normal	Turashvili Breast, *BMC Cancer, 2007*
25	Breast carcinoma – Advanced M stage	vantVeer breast, *Nature, 2002*
26	Breast carcinoma – High grade	vantVeer breast, *Nature, 2002*
27	Breast carcinoma – Metastatic event at 5 years	vantVeer breast, *Nature, 2002*
28	Breast carcinoma – Advanced M stage	Wang Breast, *Lancet, 2005*
29	Breast carcinoma – Recurrence at 3 years	Wang Breast, *Lancet, 2005*
30	Breast carcinoma – Recurrence at 5 years	Wang Breast, *Lancet, 2005*
31	Invasive ductal breast carcinoma – High grade	Zhao Breast, *Mol Biol Cell, 2004*

Summary of 31 different microarray-based breast cancer studies retrieved from the Oncomine database (OncomineTM, Compendia Bioscience, Ann Arbor, MI, https://www.oncomine.org/).

Supporting information Table S3.

FBXO28 Expression in Human Breast Cancer Correlates with a gene Set Associated with MYC and p300 Activity as Determined by ChIP-seq

	MYC binding MCF7			MYC binding K562			p300/MYC binding K562		
FBXO28 Correlation	Yes	No	p value	Yes	No	p value	Yes	No	p value
- Positive	30	5		29	6		19	10	
- Negative	16	51	**2.9e-09**	8	59	**3.8e-15**	2	6	**0.055**

A gene expression network containing FBXO28 was extracted using data representing 327 clinical breast cancer specimens (Loi S, 2007). 102 genes were found to be highly correlated to FBXO28 expression (Spearman correlations above 0.5 or below -0.5). This gene list was subsequently compared to a database containing known Myc-upregulated targets (myccancergene.org) and checked for MYC binding at promoter sequences in MCF7 and K562 cells, and for MYC/p300 binding in K562 cells, using data from the ENCODE project (http://genome.ucsc.edu/ENCODE/analyses).

Supporting information Table S4. Excel spread sheet (see separate file)

Supporting information Table S5A.

Correlation Between Clinicopathological Characteristics of Breast Cancer Patients with Low (<25%) and High (>25%) Nuclear Fraction (NF) of Phosphorylated FBXO28 (Cohort 2, N = 144)

Characteristic	Low Level (NF≤25%), N = 43	High Level (NF≥25%), N = 65	[a]p Value (chi-square)	[b]p Value (spearman rho)
Age (years)				
— ≤ 50	5	10		
— > 50	38	55	0.581	0.094 (r = -0.162)
Tumor size (mm)				
—≤ 20	24	25		
—> 20	19	40	0.076	**0.039** (r = 0.199)
ER status				
— Negative	2	12		
— Positive	41	53	**0.036**	**< 0.001** (r = - 0.337)
PR status				
— Negative	11	26		
— Positive	32	39	0.122	0.135 (r = - 0.145)
Grade (NHG)				
— I	8	3		
— II	29	23		
— III	6	39	**< 0.001**	**< 0.001** (r = 0.462)
Nodal Status				
— Negative	21	31		
— Positive	19	28	0.997	0.922 (r = - 0.010)
— Unknown	3	6		
HER2 Status				
— Negative	28	39		
— 1+	10	12		
— 2+	3	8		
— 3+	1	6	0.387	0.503 (r = 0.065)
— Unknown	1			

[a]p value derived with pearson chi-square test
[b]p value derived with spearman rho correlation, r= spearman's coefficient
ER = oestrogen receptor, PR = progesterone receptor, NHG = Nottingham histological grade, HER2 = human epidermal growth factor receptor 2, IHC = immunohistochemistry. Significant correlations (p < 0.05) are indicated by bold numbers.

Supporting information Table S5B.

Cox Univariate Analysis for Overall Survival (OS) and Breast-Cancer Specific Survival (BCSS) According to the Nuclear Fraction (NF) of Phosphorylated FBXO28 and Other Established Clinocpathological Parameters.

Prognostic factor	HR (OS)	95% CI	p Value (OS)	HR (BCSS)	95% CI	p Value (BCSS)
[a] FBXO28 (NF)	3.27	1,43 – 7,47	**0.005**	4.30	1,26 – 14,70	**0.020**
Age (years)	1.07	1,04 – 1,09	**< 0.001**	1.05	1,02 – 1,09	**0.003**
Tumor size (mm)	1.02	1,01 – 1,03	**< 0.001**	1.02	1,00 – 1,03	**0.014**
NHG (I-III)	2.11	1,28 – 3,49	**0.004**	5.14	2,03 – 12,99	**0.001**
ER status	0.39	0,19 – 0,79	**0.009**	0.23	0,10 – 0,55	**0.001**
Nodal status	1.09	1,02 – 1,17	**0.013**	1.15	1,07 – 1,24	**< 0.001**
HER2	1.02	0,88 – 1,19	0.777	1.07	0,89 – 1,29	0.454

[a] 25% NF cut-off was used to estimate the impact of phosphorylated FBXO28 on OS and BCSS.
NF = Nucelar Fraction, HR = Hazard Ratio, OS = Overall Survival, BCSS = Breast-Cancer Specific Survival,
CI = Confidence Interval
ER = oestrogen receptor, NHG = Nottingham histological grade, HER2 = human epidermal growth factor receptor 2
Significant correlations ($p < 0.05$) are indicated by bold numbers

Supporting information Table S5C.

Table S4C. Cox Multivariate Analysis for Overall Survival (OS) and Breast Cancer Specific Survival (BCSS) According to the Nuclear Fraction (NF) of Phosphorylated FBXO28 (Cohort 2, N = 144)

Survival	HR	95% CI	p value
OS	3,19	1,22 – 8,33	**0.018**
BCSS	3,85	0,80 – 18,63	0.094

HR = Hazard Ratio, CI= confidence interval. Significant correlations ($p < 0.05$) are indicated by bold numbers.
Multivariate analysis included adjustment for patient age, tumor size, ER status, nodal status, HER2 and grade. A cut-off of 25% nuclear fraction (NF) was used for determination of FBXO28 phosphorylation status.

Supporting information Table S6A.

**Correlation Between Clinicopathological Characteristics and FBXO28 Protein Phosphorylation and Expression in the Nucleus (Cohort 3, N = 498)**		
Characteristic	FBXO28 Phosphorylation (NF)	FBXO28 Expression (NI)
Age (years)		
— Correlation Coefficient (r)	-0.143	-0.056
— Significance	**0.003**	0.225
— N	442	469
Tumor size (mm)		
— Correlation Coefficient (r)	0.185	0.046
— Significance	**< 0.001**	0.321
— N	441	468
Oestrogen Receptor (ER) status		
— Correlation Coefficient (r)	-0.315	-0.098
— Significance	**< 0.001**	**0.035**
— N	442	
Progesterone Receptor (PR) status		
— Correlation Coefficient (r)	-0.199	-0.130
— Significance	**< 0.001**	**0.005**
— N	442	457
Grade (NHG)		
— Correlation Coefficient (r)	0.366	0.170
— Significance	**< 0.001**	**< 0.001**
— N	441	468
Nodal Status		
— Correlation Coefficient (r)	0.064	0.105
— Significance	0.207	**0.032**
— N	395	419
HER2 Status		
— Correlation Coefficient (r)	0.046	ND
— Significance	0.342	ND
— N	420	ND

p value derived with spearman rho correlation, r= spearman's coefficient
Significant correlations ($p < 0.05$, 2-tailed) are indicated by bold numbers
NHG = Nottingham histological grade, HER2 = human epidermal growth factor receptor 2
FBXO28 phosphorylation was assessed as the nuclear fraction (NF) of positive tumor cells and FBXO28 expression as the nuclear staining intensity (NI) using anti-pS344-FBXO28 and anti-FBXO28 antibodies, respectively.

Supporting information Table S6B.

Cox Multivariate Analysis for Overall Survival (OS) and Breast Cancer Specific Survival (BCSS) According to FBXO28 Phosphorylation and Expression Level (Cohort 3, N = 498)

	[a]FBXO28 Phosphorylation (NF)			[b]FBXO28 Expression (NI)		
Survival	HR	95% CI	p value	HR	95% CI	p value
OS	1,67	1,09 – 2,56	**0.018**	1,59	1,05 – 2,38	**0.026**
BCSS	2,69	1,40 – 5,16	**0.003**	2,00	1,13 – 3,50	**0.017**

HR = Hazard Ratio, CI= confidence interval. Significant correlations ($p < 0.05$) are indicated by bold numbers.
Multivariate analysis included adjustment for patient age, tumor size, ER status, nodal status and grade. For survival analysis, dichotomised variables defined as low or high levels of FBXO28 phosphorylation (cut-off 25% nuclear fraction (NF)), and low or high levels of FBXO28 expression (categorized as nuclear staining intensity (NI)), were used.

Supporting information Table S6C.

Cox Univariate Analysis for Overall Survival (OS) and Breast-Cancer Specific Survival (BCSS) According to FBXO28 Phosphorylation and Expression (Cohort 3, N = 498)

	[a]FBXO28 Phosphorylation (NF)			[b]FBXO28 Expression (NI)		
Survival	HR	95% CI	p value	HR	95% CI	p value
OS	1,65	1,18 – 2,30	**0.003**	1,60	1,14 – 2,24	**0.007**
BCSS	3,29	1,91 – 5,65	**< 0.001**	1,91	1,15 – 3,17	**0.013**

HR = Hazard Ratio, CI= confidence interval. Significant correlations ($p < 0.05$) are indicated by bold numbers.
For survival analysis , [a]25% NF cut-off was used to estimate the impact of phosphorylated FBXO28, [b]FBXO28 expression was categorized as dichotomised nuclear staining intensity (low vs high level). NF= Nucelar Fraction, NI = Nuclear Intensity, HR = Hazard Ratio, OS = Overall Survival, BCSS = Breast-Cancer Specific Survival, CI = Confidence Interval.

SUPPORTING INFORMATION - MATERIALS AND METHODS

Cell Lines

Cell lines were grown in media according to the guidelines at ATCC, or as previously described (Sangfelt et al, 2008). The following cell lines were used in this study; HEK293, HCT116, U-937, MCF7, U2OS, A549, KPL4, HeLa, Cos-7, KPL4, A549, IMR90, Human diploid fibroblasts (HDF), immortalized breast epithelial cells (IME), *p53-/-* mouse embryonic fibroblasts (MEFs), and parental TGR1 (*MYC+/+*) and *MYC*-null HO1579 (*MYC-/-*) Rat1 fibroblasts. Tet-On U2OS cells were cultured according to the protocol (Clontech). HCT116 *FBXW7+/+* and *FBXW7-/-* cells was a gift from B. Vogelstein and were grown in McCoy's medium. Media was supplemented with 10% (v/v) fetal bovine serum, 2 mM L-glutamine, 100 U/ml penicillin, and 100 g/ml streptomycin. Tet-On U2OS FBXO28 WT and ΔF-box stable cells were maintained in tetracycline-approved FBS (Clontech) supplemented with 0.2 mg/ml G418 and 0.1 μg/ml of puromycin (Sigma). All cells were kept at 37˚C in a humidified incubator with 5% CO_2.

Plasmids and siRNAs

Details of the construction of expression plasmids are available from the authors upon request. The following constructs were used in this study: pcDNA-3Myc-, pCMV2-Flag-FBXO28, pEBG-GST-FBXO28 (WT, ΔF, S344A and S344E), pBabe-puro-FBXO28 (WT and ΔF), , pcDNA-HA-Ubiquitin, pcDNA-HA-CDK1/2 (WT and DN) (purchased

from Addgene, Inc), pcDNA-HA-Cul1, pcDNA-Skp1, pcDNA-3Myc-Roc1, TRIPZ inducible lentiviral vector expressing FBXO28 shRNA or TRIPZ empty vector control (Thermo Fisher Scientific, MA, USA). All MYC expression constructs and the M4mintk-Luc and RSV-β-Galactosidase reporters have been previously described (von der Lehr et al, 2003). SIK and the TGFβ-responsive HepG2-CAGA luciferase constructs were kindly provided by Dr. Aris Moustakas.

siRNA transfections were performed using either single or pooled siRNA oligonucleotides (Dharmacon): *siFBXO28-1*: AGCUCCGCCUGGUUUGUAA, *siFBXO28-2*: 5UCAACGAGCUCAUGAAGUAUU, *siFBXO28-3*: GUGGAGAGGUACCAUAAUC). Knockdown efficiency was determined by qRT-PCR and/or immunoblot analysis compared to scrambled control siRNAs (Dharmacon).

Treatments, Kinase Assays and Antibodies

For synchronization experiments, cells were arrested at G2/M by sequential culture in 2mM thymidine for 14 h and 125ng/ml nocodazole for 12 h, rounded cells were collected (mitotic shake-off) and, where indicated, released in fresh culture medium. For S-phase arrest, cells were incubated with 2mM thymidine for 16 h, released in fresh media for 9 h and treated again with thymidine for 16 h. Phosphatase treatment was carried out in a volume of 100 µl total cell lysate in LSLD buffer (50 mM NaCl, 0.1% Tween-20, 10% glycerol, 50 mM HEPES) with 10 mM $MgCl_2$, 1 mM DTT and protease inhibitor cocktail, using 10 U calf intestinal alkaline phosphatase (New England Biolabs), either in the absence or in the presence of phosphatase inhibitors (1 mM sodium orthovanadate, 5

mM sodium fluoride and 5mM β-glycerophosphate), for 1 hr at 37˚C. For kinase assays, pCDNA-3xMyc-FBXO28-WT and FBXO28-S344A constructs were *in vitro* transcribed by T7 RNA polymerase and translated using the TNT Coupled Reticulocyte Lysate Systems, according to the manufacturer's instructions (Promega). 2 μl of the *in vitro* translated proteins was incubated with or without recombinant cyclin-CDK proteins GST-CDK2/CycE, CDK1/CycB (both from Cell Signalling), or GST-CDK2/CycA (Calbiochem) in kinase buffer (50 mM HEPES pH 7.5, 10 mM $MgCl_2$, 1 μM DTT, 20 mM NaF, 10 mM β-glycerophosphate, and 0.1 mM ATP) at 30°C for 30 min. The kinase reaction was stopped by the addition of SDS-PAGE sample buffer containing 0.1M DTT. The products were boiled for 5 min before being resolved by SDS-PAGE gel analysis immunoblotted with the indicated antibodies.

The following primary antibodies were purchased from Santa Cruz Biotechnology: anti-HA (Y11), anti-MAX (C-17), anti-MYC (N-262, C-33), anti-p300 (N-15), anti-cyclin A (H432), anti-CDK2 (M2), anti-MYC tag (9E10), anti-GST (B-14), anti-cyclin E (HE12), anti-GFP (B-2), anti-tubulin (B-7), anti-lamin A/C (H-110), anti PLK (F-8); from Sigma: anti-β-Actin, anti-MCM7 (DCS-141) and anti-Flag (M2). Antibodies were also purchased from Abcam: anti-GAPDH, and anti-Ki67; Abnova: anti-FBXO28 (BO1P); Upstate: anti-acetyl histone H4 (#06-866); Cell Signaling: anti-Skp1, anti-Cul1 and anti-cyclin B1 (V152). To generate a mono-specific antibody recognizing FBXO28, a protein fragment spanning amino acids 68 to 190 was identified due to its low sequence similarity to other human proteins and a gene encoding this fragment was cloned using the appropriate primers (Agaton et al, 2003). Antibody production, purification and characterization of the protein fragment were performed as previously

described (Agaton et al, 2003; Nilsson et al, 2005). Phospho-specific, pS344-FBXO28, rabbit polyclonal antibodies were generated by Innovagen (Lund, Sweden) using the peptide sequence -CSGSGQNEE(pS)PRKRK-. All other primary antibodies used in this study was created by the Human Proteome Project (HPP, http://www.hupo.org/research/hpp/) and each antibody was tested according to Human Protein Atlas (HPA)-standard quality assurance procedures (http://www.proteinatlas.org/). The following secondary antibodies were purchased from Rockland: HRP-conjugated anti-Mouse IgG, Cell Signalling: anti-rabbit IgG, DAKO: anti-goat IgG. Antibodies conjugated to fluorescein isothiocyanate (FITC) (DAKO) or Texas Red (Vector Laboratories) were used for indirect immunofluorescence microscopy analysis.

Cell Proliferation Analyses and siRNA functional library screens

The indicated cell lines were transfected with siRNA oligos for 48 hours and incubated with EdU (Invitrogen) at a final concentration of 10 μM for the indicated times, at 37˚C. Cells were dissociated into a single-cell suspension, fixed with formaldehyde before permeabilization, staining, and analysis of DNA content by FACS analysis according to the manufacturer (Molecular Probes). Alternatively, doxycyclin-inducible TRIPZ lentiviral vector co-expressing RFP and FBXO28 shRNA or TRIPZ empty vector control (shEV), were transfected into 293T cells along with the vectors of pVSV-G and pPack (System Biosciences, CA, USA), to generate shFBXO28 lentiviruses. Parental TGR1 (*MYC*+/+) and myc null HO1579 (*MYC*+/+) Rat1 fibroblasts were transduced with

shFBXO28 or shEV lentiviruses with a multiplicity of infection (MOI) of 50, and incubated in DMEM media containing polybrene 8µg/ml. Transduced cells were grown on coverslips, induced with doxycyclin (5ug/ml) and cells were allowed to incorporated EdU (10µM) for 15 min. (*MYC*+/+) or 18 hours (*MYC*-/-) before fixation and staining using EdU azide. Transfection of the shRNA was demonstrated by RFP fluorescence. Down-regulation of FBXO28 was confirmed with immunostainings with primary anti-FBXO28 antibodies and incorporation of EdU was demonstrated with the Click IT EdU Alexa Fluor 488 imaging kit (Molecular Probes, Life Technologies). Cell nuclei were visualized by Hoechst-staining. Images were captured at 40x magnification (Zeiss Plan-NeoFluar 40x /NA 1.3 oil DIC) using a ZEISS LSM510 META laser scanning confocal microscope. The confocal images were automatically analyzed with the image analysis software Volocity (ver. 6.2.1, Perkin Elmer). Based on the Hoechst staining, nuclear regions were identified and the intensity values for the RFP and EdU fluorescence determined. An arbitrary threshold for the EdU fluorescence signal was used as the cut-off value to identify cells at the proliferative stage. Fields for analysis were chosen at random, and at least 100 bright RFP cells were scored for EdU staining.

Cell proliferation was also analysed following siRNA-mediated knockdown in accordance with the long-term silencing protocol from the manufacturer (Qiagen). Briefly, U2OS cells were transfected with siRNA oligonucleotides for two consecutive days. 48 hours after the second transfection, cells were trypsinized, split into triplicate dishes and re-transfected with siRNAs. Cells were collected daily and counted. Knockdown efficiency was assessed by immunoblotting for FBXO28. To measure proliferation rates upon induction of FBXO28 expression (WT and ΔF compared to

empty vector cells), Tet-On U2OS stable cells were plated at equal numbers and induced with doxycycline for 48 hours. Cells were then detached with trypsin, counted and re-plated at equal numbers in triplicates every day in complete medium supplemented with doxycycline. Cells were counted every day for 4 days and cell numbers were plotted. For colony formation assays on plastic, equal numbers of U2OS cells were transfected with the indicated expression plasmids and selected in G418 (500 μg/ml) for two weeks. Cell colonies were stained with Giemsa (Sigma).

The cell spot microarray (CSMA) technology was used to screen for loss of proliferation upon siRNA-mediated gene silencing (Rantala et al, 2011). Briefly, in the primary analysis, MCF7, KPL4, A549, and HCT116 cells were reverse-transfected on CSMAs composed of custom siRNA oligonucleotides targeting F-box genes (Thermo Scientific Dharmacon). After 72 hours, cells were allowed to incorporate EdU and then analyzed by fluorescence microscopy using EdU and DAPI stains. In the secondary analysis, KPL4 cells were transfected with the human druggable genome siRNA library v1.0 (QiagenAmbion, Inc.) containing two individually printed siRNA constructs for 6,135 human genes. Replicates of two different negative control siRNA constructs (Qiagen and Ambion Inc.) were used as negative controls in both analyses. After 72 hours knockdown, cells were fixed and stained for fluorescence microscopic analysis of cell proliferation with EdU and DAPI stains or anti-Ki-67 antibodies and SYTO60 stain (Invitrogen). The arrays were imaged and analyzed with an Olympus scanR (Olympus, Munich, Germany) automated high content imager. Z-score values calculated in comparison to the mean signal ratios and standard deviation across all samples was used to identify siRNAs inducing a significant change in EdU or Ki-67 signal.

Microarray Experiments

For microarray experiments, HCT116 cells were transfected with siRNA oligonucleotides targeting *FBXO28* (sequence #2) or a scrambled control sequence. Briefly, cells were harvested 48 hours post-transfection and 20 µg of total RNA was used for random-primed cDNA synthesis and labelling along with Total Human Reference RNA (Stratagene). For each microarray, labelled reference and sample cDNA were mixed with balanced amounts of dye and hybridized for 24 hours. Image analysis was carried out using the Genepix Pro 5.1 software. Low-level analysis including filtering and normalization was carried out using the KTH package for microarray analysis (Wirta et al, 2007). The differentially expressed genes from the array (Operon Biotechnologies) was analyzed using the Gene Ontology Tree Machine (http://bioinfo.vanderbilt.edu/webgestalt) and patterns of gene expression were analyzed using the GSEA software (Subramanian et al, 2005).

Immunofluorescence Microscopy and In Situ Proximity Ligation Analysis

Cells on coverslips were fixed in 4 % PFA (wt/vol) in PBS (pH 7.6) for 10 minutes at room temperature, permeabilized for 5 minutes at 4˚C with 0.1% Triton X-100 in PBS and blocked in blocking buffer (2% BSA, 0.2% Tween-20, 5% glycerol in PBS). The cells were then incubated with the indicated primary antibodies in blocking buffer for 1 hour at room temperature and washed three times in TBS with 0.05% Tween-20 (TBS-T), followed by incubation with appropriate secondary antibodies conjugated to

fluorescein isothiocyanate (FITC; 1:200) or Texas Red (1:200), and then counterstained with Hoechst or DAPI for DNA labeling.

In situ proximity ligation assay (isPLA) is a sensitive technique that allows the visualization of endogenous protein-protein interactions in fixed cells in the subcellular compartment where the interaction occurs (Soderberg et al, 2006). Briefly, primary antibodies bound to the target proteins are detected using a pair of secondary antibodies, to which oligonucleotides have been attached. These are known as proximity probes If the proteins interact, these attached oligonucleotides will be ligated with the help of DNA ligase, and they can be used as a template for rolling circle amplification. The product is hybridized to a fluorescently labeled probed and visualized by fluorescence microscopy. For isPLA, cells grown on chambered slides (BD Biosciences) were fixed and permeabilized as for immunofluorescence, and blocked in 250 μg/ml BSA, 2.5 mM cysteine, 2.5 ng/μl sonicated salmon sperm DNA and 0.05% Tween 20 in TBS with 5 mM EDTA for 2 h at 37 ˚C before overnight incubation at 4 ˚C with primary antibodies (MYC (C-33), MAX (C-17), and FBXO28-specific antibodies described above) in blocking solution. After washing three times for one minute with TBST (1× TBS with 0.05% Tween-20), proximity probes (donkey anti-mouse, 1:5, and donkey anti-rabbit, 1:15, Olink Bioscience, Uppsala, Sweden) in blocking solution were added to the slides and incubated for 1 h at 37 ˚C. The slides were then washed three times for five minutes with TBST to remove unbound probes. Subsequent isPLA reaction was performed as described previously (Soderberg et al, 2006) using connector probes P-CTATTAGCGTCCAGTGAATGCGAGTCCGTCTAAGAGAGT-AGTACAGCAGCCGTCAAGAGTGTCTA and P-GTTCTGTCATATTTAAGCGTCTT

AA and counterstained with 1 mM Hoechst 33342 (Sigma). Fluorescent images were taken with ×63 objective using a camera (C4880, Hamamatsu) on a fluorescent microscope (Leica HC) using the HiPic software. Images were colored and merged using Photoshop.

Mass Spectrometry

Mass-spectrometry analysis was performed essentially as previously described (Lerner et al., 2007; Nielsen et al., 2005). Briefly, 1 mg total protein extract was used for affinity purification of endogenous FBXO28 associated protein complexes using 2 μg of FBXO28 antibody for 2 hrs at 4°C. Protein complexes were collected on Gammabind sepharose beads for 1 hour, extensively was washed before boiling and separation on SDS-PAGE gels. Entire lanes were excised into 10 kDa pieces and each sample was in-gel reduced, alkylated and digested with modified sequence-grade trypsin (Promega, Madison, WI) and FBXO28-assocaited proteins were identified by nanoflow liquid chromatography tandem mass spectrometry (Lerner et al, 2007) using standard protocols and putative FBXO28-associated proteins identified by nanoflow liquid chromatography tandem mass spectrometry (Nielsen et al, 2005). All experiments were performed on a 7-tesla LTQ-FT mass spectrometer (Thermo Electron, Bremen, Germany), modified with a nanoelectrospray ion source (Proxeon Biosystems, Odense, Denmark). Nanoflow liquid chromatography Tandem mass spectrometry (LC-MS/MS) and analysis were carried out as previously described (Lerner et al, 2007; Nielsen et al, 2005; Savitski et al, 2005; Zubarev et al, 2000).

Ubiquitylation and Protein Turnover Assays

In vivo and *in vitro* ubiquitination assays were performed essentially as previously described (Lerner et al, 2007). Briefly, for *in vivo* ubiquitylation experiments, cells (HCT116-FBXW7-/-, HCT116-FBXW7+/+, and U2OS) were co-transfected with the indicated expression constructs or siRNAs. Prior to harvesting, cells were treated with MG132 for 3-5 hours followed by lysis in 1% SDS and 10 mM *N*-ethylmaleimide to disrupt noncovalent interactions. Total protein extracts were boiled for 10 minutes, sonicated, and diluted in M-RIPA buffer (without SDS). High-molecular-weight MYC poly-ubiquitin conjugates were detected by anti-HA or anti-MYC immunoblot analysis. For *in vitro* ubiquitylation experiments, equal number of cells expressing GST-tagged WT-FBXO28 and S344E-FBXO28, and three times as many cells expressing S344A-FBXO28 were used to obtain comparable expression. GST-tagged proteins were purified on glutathione sepharose beads (GE healthcare), eluted with reduced glutathione (10mM) and reconstituted in ubiquitination buffer (25mM Tris-HCl pH 7.6, 5mM MgCl2, 100mM NaCl) including protease and phosphatase inhibitors. FLAG-MYC was purified from pre B-cells stably expressing FLAG-tagged MYC using M2 beads (Sigma). MYC protein bound to beads were subsequently incubated for 90 minutes at 31°C in ubiquitination buffer containing 100ng of E1, 150ng E2 enzymes (a panel of 10 different E2 enzymes were tested), 600ng eluted GST-FBXO28, 5µg of HA-Ub, 2mM ATP and 2mM DTT. The reactions were terminated by boiling in SDS samples buffer and ubiquitylated proteins resolved by SDS-PAGE followed by immunoblot analysis with anti-HA or anti-FLAG antibodies.

For protein turnover assays, $6x10^5$ cells were seeded in 6-well plates, incubated overnight and transfected for 24-48 hours using the indicated expression constructs or siRNA oligos. Cycloheximide (100 µg/ml) was added directly to the media to block *de novo* protein synthesis and cells were harvested at different time-points in M-RIPA buffer. Protein turnover was analyzed by immunoblotting with the indicated antibodies.

Chromatin Immunoprecipitation

Cells at approximately 80% confluency were harvested followed by crosslinking with formaldehyde at a final concentration of 1.1%. Glycine (0.125M) was added to neutralize the formaldehyde and cells were centrifuged at 1500rpm for 5 minutes at 4°C. Cells were resuspended in a buffer containing 0.25% Triton-X100, 10mM Tris (pH 8.0), 10 mM EDTA, 0.5mM EGTA and incubated with rotation at room temperature (RT) for 15 minutes. Cells were then centrifuged and washed with washing buffer containing 200mM NaCl, 10mM Tris (pH 8.0), 10 mM EDTA, 0.5mM EGTA with rotation at room temperature for 15 min. Cells were centrifuged and lysed in ice-cold RIPA buffer containing 150mM NaCl, 10mM Tris (pH 7.5), 1% NP40, 1% DOC, 0.1% SDS, 1mM ETDA. Chromatin DNA was sonicated to an average size of 300–600 bp using a Soniprep 150 sonifier. Immunoprecipitation was carried out in RIPA buffer overnight at 4ºC with rotation, using 2µg of the indicated antibodies. Immunoprecipitates were incubated with protein A/G-sepharose beads (Millipore) for 2 hours and washed two times in RIPA buffer and TE buffer. Immunoprecipitates were incubated with RNase A for 30 min at 37ºC, followed by 20 mg/ml Proteinase K for 6 hours at 37ºC, and 6 hours at 66ºC to reverse the crosslinking. DNA was recovered by phenol extraction and ethanol

precipitation and resuspended in TE buffer. Samples were analyzed by qPCR using gene-specific primers (provided by authors upon request) and normalized to input DNA.

Primary Breast Tumor Specimens and Tumor Analysis

Breast cancer specimens obtained from the Department of Obstetrics and Gynecology, Innsbruck Medical University, Austria, were processed for Western blot analyses as previously described (Spruck et al, 2006). Clinicopathologic features for this collection of samples have been previously reported (Spruck et al, 2006). Two independent cohorts of breast cancer specimens obtained from patients diagnosed with breast cancer at Malmö University Hospital, Sweden, was used for TMA analysis. Patients in Cohort 2 (Elkabets et al, 2011; Svensson et al, 2011) were diagnosed between 2001 and 2002 and the median age at diagnosis was 65 years (range 34-97) with a median follow-up time for overall survival (OS) of 52 months. Complete treatment data was available for 143 patients, of whom 67 (47%) had received adjuvant tamoxifen, 3 (2%) an aromatase inhibitor and 25 (17%) were treated with a combination of tamoxifen and aromatase inhibitor. Patients included in Cohort 3 (n=498) were diagnosed between 1988 and 1992 and characteristics are described in detail elsewhere (Svensson et al, 2011). Cohort 3 is a part of an original cohort of 512 patients (Borgquist et al, 2008). Median age at diagnosis was 65 years (range 27-96) with a median follow-up time for first breast cancer event of 128 months (range 0-207 months). Complete treatment information was obtained for 379 (76%) patients, of whom 160 (42%) had been treated with adjuvant tamoxifen. Adjuvant

systemic chemotherapy data was available for 382 patients, of whom 23 (6%) had received treatment.

All human tissue samples were histopathologically re-evaluated on slides stained with haematoxylin & eosin prior to TMA construction. The TMAs were essentially made as described previously (Kampf C, 2004; Kononen et al, 1998). For each patient, duplicate 1 mm cores were taken from areas representative of invasive cancer and inserted in a recipient block. Automated IHC (Lab Vision Autostainer 480, Thermo Fisher Scientific, MA, USA) was done as previously described (Paavilainen et al, 2008) using a primary antibody detecting total FBXO28 protein (HPA003289, Atlas Antibodies, Stockholm, Sweden) or an antibody detecting phosphorylated FBXO28 protein (anti-pS344-FBXO28). The Aperio ScanScope XT system (Aperio Technologies, CA, USA) was used to scan IHC stained slides with a 20X objective. The digitalized images of IHC stainings were manually annotated using the Aperio ImageScope Viewer v.10.2.1.2314 (Aperio Technologies). Nuclear staining of phosphorylated FBXO28 protein was assessed as the fraction of positive nuclei (0-1%, 2-10%, 11-25%, 26-50%, 51-75% and 76-100%,). Total nuclear FBXO28 protein levels were assessed as nuclear staining intensity (N1<N2<N3, weak to strong intensity).

Data mining

Oncomine™ (Compendia Bioscience, Ann Arbor, MI) was used for analysis and visualization of FBXO28 gene expression data (http://www.oncomine.org). The data in Figure S3A were collected from the *in silico* transcriptomics database of the GeneSapiens

system (www.genesapiens.org). Proteins identified as putative FBXO28-interacting proteins were subjected to Ingenuity analysis (www.ingenuity.com).

In Vitro and In Vivo Tumorigenicity Analysis

p53-null MEFs were transduced with indicated retroviruses. Cells were selected for 48 hours with 1.5 µg/ml puromycin and 50,000 cells were mixed with 0.35% (w/v) top agar and plated onto 0.5% (w/v) basal agar. Twenty-one days after plating, the number of colonies of similar size was scored, and no significant changes in colony size were observed between conditions. For focus formation assays, transduced cells were allowed to grow to confluency in non-selective complete medium for approximately 15 days. Plates (in triplicates) were then stained with 0.01% (w/v) crystal violet and the number of foci was counted.

For analysis of tumor growth in vivo, five week-old athymic male nu/nu Swiss mice (Charles River Laboratory, Sta Perpetua, Spain) were subcutaneously (s.c.) injected at both flanks with MYC-transformed MEFs (10^6 cells resuspended in 100 µl of PBS) stably transduced with ΔF-FBXO28 viruses. In vivo growth was analyzed using two individual MYC-transformed clones. Tumors were measured periodically, and the volume was calculated as (length)x($width^2/2$). Mice were sacrificed by CO_2 inhalation and tumors were excised and analyzed as indicated.

SUPPORTING INFORMATION - REFERENCES

Agaton C, Galli J, Hoiden Guthenberg I, Janzon L, Hansson M, Asplund A, Brundell E, Lindberg S, Ruthberg I, Wester K, Wurtz D, Hoog C, Lundeberg J, Stahl S, Ponten F, Uhlen M (2003) Affinity proteomics for systematic protein profiling of chromosome 21 gene products in human tissues. *Mol Cell Proteomics* **2:** 405-414

Borgquist S, Holm C, Stendahl M, Anagnostaki L, Landberg G, Jirstrom K (2008) Oestrogen receptors alpha and beta show different associations to clinicopathological parameters and their co-expression might predict a better response to endocrine treatment in breast cancer. *J Clin Pathol* **61:** 197-203

Elkabets M, Gifford AM, Scheel C, Nilsson B, Reinhardt F, Bray MA, Carpenter AE, Jirstrom K, Magnusson K, Ebert BL, Ponten F, Weinberg RA, McAllister SS (2011) Human tumors instigate granulin-expressing hematopoietic cells that promote malignancy by activating stromal fibroblasts in mice. *The Journal of clinical investigation* **121:** 784-799

Kampf C AA, Wester K. (2004) Antibody-based tissue profiling as a tool for clinical proteomics. *Clinical Proteomics***:** 285-285

Kononen J, Bubendorf L, Kallioniemi A, Barlund M, Schraml P, Leighton S, Torhorst J, Mihatsch MJ, Sauter G, Kallioniemi OP (1998) Tissue microarrays for high-throughput molecular profiling of tumor specimens. *Nat Med* **4:** 844-847

Lerner M, Corcoran M, Cepeda D, Nielsen ML, Zubarev R, Ponten F, Uhlen M, Hober S, Grander D, Sangfelt O (2007) The RBCC gene RFP2 (Leu5) encodes a novel transmembrane E3 ubiquitin ligase involved in ERAD. *Mol Biol Cell* **18:** 1670-1682

Loi S H-KB, Desmedt C, Lallemand F, Tutt AM, Gillet C, Ellis P, Harris A, Bergh J, Foekens JA, Klijn JG, Larsimont D, Buyse M, Bontempi G, Delorenzi M, Piccart MJ, Sotiriou C. (2007) Definition of clinically distinct molecular subtypes in estrogen receptor-positive breast carcinomas through genomic grade. *J Clin Oncol* **25:** 1239-1246

Nielsen ML, Savitski MM, Zubarev RA (2005) Improving protein identification using complementary fragmentation techniques in fourier transform mass spectrometry. *Mol Cell Proteomics* **4:** 835-845

Nilsson P, Paavilainen L, Larsson K, Odling J, Sundberg M, Andersson AC, Kampf C, Persson A, Al-Khalili Szigyarto C, Ottosson J, Bjorling E, Hober S, Wernerus H, Wester

K, Ponten F, Uhlen M (2005) Towards a human proteome atlas: high-throughput generation of mono-specific antibodies for tissue profiling. *Proteomics* **5:** 4327-4337

Paavilainen L, Wernerus H, Nilsson P, Uhlen M, Hober S, Wester K, Ponten F (2008) Evaluation of monospecific antibodies: a comparison study with commercial analogs using immunohistochemistry on tissue microarrays. *Appl Immunohistochem Mol Morphol* **16:** 493-502

Rantala JK, Makela R, Aaltola AR, Laasola P, Mpindi JP, Nees M, Saviranta P, Kallioniemi O (2011) A cell spot microarray method for production of high density siRNA transfection microarrays. *BMC Genomics* **12:** 162

Sangfelt O, Cepeda D, Malyukova A, van Drogen F, Reed SI (2008) Both SCF(Cdc4alpha) and SCF(Cdc4gamma) are required for cyclin E turnover in cell lines that do not overexpress cyclin E. *Cell Cycle* **7:** 1075-1082

Savitski MM, Nielsen ML, Zubarev RA (2005) New data base-independent, sequence tag-based scoring of peptide MS/MS data validates Mowse scores, recovers below threshold data, singles out modified peptides, and assesses the quality of MS/MS techniques. *Mol Cell Proteomics* **4:** 1180-1188

Soderberg O, Gullberg M, Jarvius M, Ridderstrale K, Leuchowius KJ, Jarvius J, Wester K, Hydbring P, Bahram F, Larsson LG, Landegren U (2006) Direct observation of individual endogenous protein complexes in situ by proximity ligation. *Nat Methods* **3:** 995-1000

Spruck C, Sun D, Fiegl H, Marth C, Mueller-Holzner E, Goebel G, Widschwendter M, Reed SI (2006) Detection of low molecular weight derivatives of cyclin E1 is a function of cyclin E1 protein levels in breast cancer. *Cancer Res* **66:** 7355-7360

Subramanian A, Tamayo P, Mootha VK, Mukherjee S, Ebert BL, Gillette MA, Paulovich A, Pomeroy SL, Golub TR, Lander ES, Mesirov JP (2005) Gene set enrichment analysis: a knowledge-based approach for interpreting genome-wide expression profiles. *Proc Natl Acad Sci U S A* **102:** 15545-15550

Svensson KJ, Christianson HC, Kucharzewska P, Fagerstrom V, Lundstedt L, Borgquist S, Jirstrom K, Belting M (2011) Chondroitin sulfate expression predicts poor outcome in breast cancer. *International journal of oncology* **39:** 1421-1428

von der Lehr N, Johansson S, Wu SQ, Bahram F, Castell A, Cetinkaya C, Hydbring P, Weidung I, Nakayama K, Nakayama KI, Soderberg O, Kerppola TK, Larsson LG (2003) The F-Box protein Skp2 participates in c-Myc protelosornal degradation and acts as a cofactor for c-Myc-regulated transcription. *Mol Cell* **11:** 1189-1200

Whitfield ML, Sherlock G, Saldanha AJ, Murray JI, Ball CA, Alexander KE, Matese JC, Perou CM, Hurt MM, Brown PO, Botstein D (2002) Identification of genes periodically expressed in the human cell cycle and their expression in tumors. *Mol Biol Cell* **13:** 1977-2000

Wirta V, Gry M, Lindberg J, Klevebring D. (2007) kth: an R package for analysis of microarray data.

Zubarev RA, Horn DM, Fridriksson EK, Kelleher NL, Kruger NA, Lewis MA, Carpenter BK, McLafferty FW (2000) Electron capture dissociation for structural characterization of multiply charged protein cations. *Anal Chem* **72:** 563-573
